# A nanodomain-anchored scaffolding complex is required for the function and localization of phosphatidylinositol 4-kinase alpha in plants

**DOI:** 10.1093/plcell/koab135

**Published:** 2021-05-19

**Authors:** Lise C Noack, Vincent Bayle, Laia Armengot, Frédérique Rozier, Adiilah Mamode-Cassim, Floris D Stevens, Marie-Cécile Caillaud, Teun Munnik, Sébastien Mongrand, Roman Pleskot, Yvon Jaillais

**Affiliations:** Laboratoire Reproduction et Développement des Plantes, Univ Lyon, ENS de Lyon, Université Claude Bernard Lyon 1, CNRS, INRAE, F-69342, Lyon, France; Laboratoire Reproduction et Développement des Plantes, Univ Lyon, ENS de Lyon, Université Claude Bernard Lyon 1, CNRS, INRAE, F-69342, Lyon, France; Laboratoire Reproduction et Développement des Plantes, Univ Lyon, ENS de Lyon, Université Claude Bernard Lyon 1, CNRS, INRAE, F-69342, Lyon, France; Laboratoire Reproduction et Développement des Plantes, Univ Lyon, ENS de Lyon, Université Claude Bernard Lyon 1, CNRS, INRAE, F-69342, Lyon, France; Laboratoire de Biogenèse Membranaire, UMR5200, Université de Bordeaux, CNRS, 33140 Villenave d’Ornon, France; Agroécologie, AgroSup Dijon, CNRS, INRA, University Bourgogne Franche-Comté, F-21000 Dijon, France; Research Cluster Green Life Sciences, Section Plant Cell Biology, Swammerdam Institute for Life Sciences, University of Amsterdam, Amsterdam, 1090 GE, The Netherlands; Laboratoire Reproduction et Développement des Plantes, Univ Lyon, ENS de Lyon, Université Claude Bernard Lyon 1, CNRS, INRAE, F-69342, Lyon, France; Research Cluster Green Life Sciences, Section Plant Cell Biology, Swammerdam Institute for Life Sciences, University of Amsterdam, Amsterdam, 1090 GE, The Netherlands; Laboratoire de Biogenèse Membranaire, UMR5200, Université de Bordeaux, CNRS, 33140 Villenave d’Ornon, France; Institute of Experimental Botany, Academy of Sciences of the Czech Republic, 16502 Prague 6, Czech Republic; Laboratoire Reproduction et Développement des Plantes, Univ Lyon, ENS de Lyon, Université Claude Bernard Lyon 1, CNRS, INRAE, F-69342, Lyon, France

## Abstract

Phosphoinositides are low-abundant lipids that participate in the acquisition of membrane identity through their spatiotemporal enrichment in specific compartments. Phosphatidylinositol 4-phosphate (PI4P) accumulates at the plant plasma membrane driving its high electrostatic potential, and thereby facilitating interactions with polybasic regions of proteins. PI4Kα1 has been suggested to produce PI4P at the plasma membrane, but how it is recruited to this compartment is unknown. Here, we pin-point the mechanism that tethers *Arabidopsis thaliana* phosphatidylinositol 4-kinase alpha1 (PI4Kα1) to the plasma membrane via a nanodomain-anchored scaffolding complex. We established that PI4Kα1 is part of a complex composed of proteins from the NO-POLLEN-GERMINATION, EFR3-OF-PLANTS, and HYCCIN-CONTAINING families. Comprehensive knockout and knockdown strategies revealed that subunits of the PI4Kα1 complex are essential for pollen, embryonic, and post-embryonic development. We further found that the PI4Kα1 complex is immobilized in plasma membrane nanodomains. Using synthetic mis-targeting strategies, we demonstrate that a combination of lipid anchoring and scaffolding localizes PI4Kα1 to the plasma membrane, which is essential for its function. Together, this work opens perspectives on the mechanisms and function of plasma membrane nanopatterning by lipid kinases.

## Teaser

PI4Kα1 is targeted to plasma membrane nanodomains by a lipid-anchored heterotetrameric complex essential for plant cell survival, including gametophytic, embryonic, and post-embryonic development.

##  

**Figure:**
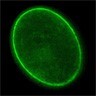


## Introduction

Eukaryotic cells are composed of several membrane-surrounded compartments. Each compartment has a unique physicochemical environment delimited by a membrane with a specific biochemical and biophysical identity (Bigay and Antonny, 2012). The membrane identity includes the nature of the lipids, the curvature, the electrostaticity, and the density of lipids at the membrane. The identity of each membrane allows the proper localization of membrane-associated proteins.

Phosphoinositides are rare anionic lipids present in membranes. Five types of phosphoinositides exist in plants—PI3P, PI4P, PI5P, PI(4,5)P_2_, and PI(3,5)P_2_—depending on the number and position of phosphates around the inositol ring (Munnik and Vermeer, 2010; Munnik and Nielsen, 2011). They accumulate differently at the plasma membrane and intracellular compartments and interact with proteins through stereo-specific or electrostatic interactions (Lemmon, 2008; Barbosa et al., 2016; Simon et al., 2016; Hirano et al., 2017). Recent work uncovered that PI4P concentrates according to an inverted gradient by comparison to their yeast and animal counterpart (Levine and Munro, 1998, 2002; Roy and Levine, 2004; Hammond et al., 2009, 2014; Simon et al., 2016; Noack and Jaillais, 2017, 2020). Indeed in budding yeast, the major PI4P pool is at the Golgi/trans-Golgi Network (TGN) compartments, while a minor pool is present at the plasma membrane (Roy and Levine, 2004). The plasma membrane pool of PI4P is produced by the PI4-kinases (PI4K) Stt4p, while Pik1p produces the PI4P pool at the TGN (Audhya et al., 2000; Audhya and Emr, 2002; Roy and Levine, 2004; Balla et al., 2005; Nakatsu et al., 2012). These two PI4P pools are essential for yeast survival and at least partially independent (Roy and Levine, 2004). In animal, three PI4K isoforms, PI4KIIIβ/PI4KIIα/PI4KIIβ, are responsible for synthetizing PI4P at the Golgi/TGN and in endosomes (Balla et al., 2002; Wei et al., 2002; Wang et al., 2003). Similar to Δ*stt4* in yeast, a PI4KIIIα loss-of-function mutation is lethal in mammals (Nakatsu et al., 2012). In PI4KIIIα conditional mutants, the pool of PI4P disappears from the plasma membrane, while the TGN structures seem to remain untouched, suggesting that the two pools could be independent (Nakatsu et al., 2012).

In plants, PI4P massively accumulates at the plasma membrane and is less abundant at the TGN (Vermeer et al., 2009; Simon et al., 2014, 2016). This PI4P accumulation at the cell surface drives the plasma membrane electrostatic field, which in turn recruits a host of signaling proteins to this compartment (Barbosa et al., 2016; Simon et al., 2016; Platre et al., 2018). Moreover, the plant TGN is the site of vesicular secretion but is also involved in endocytic sorting and recycling, which might imply regulatory mechanisms of lipid exchanges or maintenance of membrane identity between the plasma membrane and the TGN (Noack and Jaillais, 2017).

The *Arabidopsis thaliana* genome codes four PI4-kinases: PI4Kα1, PI4Kα2, PI4Kβ1, and PI4Kβ2 (Szumlanski and Nielsen, 2010). Because of the absence of expressed sequence tags of *PI4Kα2*, it is considered as a pseudogene (Mueller-Roeber and Pical, 2002). The *pi4kβ1 pi4kβ2* double mutant displays mild growth defects including tip growth phenotype with bulged root hairs and cell plate defects that suggest a defective secretory pathway (Preuss et al., 2006; Kang et al., 2011; Delage et al., 2012; Šašek et al., 2014; Antignani et al., 2015; Lin et al., 2019). In addition, *pi4kβ1 pi4kβ2* presents fewer and misshaped secretory vesicles at the TGN (Kang et al., 2011). PI4Kβ1 and PI4Kβ2 were first described as being localized to the TGN/early endosomes (EE) in root hairs (Preuss et al., 2006). This localization, as well as its accumulation at the cell plate, was later validated by electron tomography and confocal microscopy in root meristematic cells (Kang et al., 2011; Lin et al., 2019). The targeting mechanism of PI4Kβ1 at the TGN involves RabA4b, a small GTPase (Preuss et al., 2006). In addition, PI4Kβ1 recognizes, and interacts with, the curved electronegative membrane of the TGN/EE via an amphipathic lipid packing sensor motif preceded by cationic amino acids (Platre et al., 2018).

In contrast, PI4Kα1 localizes at the plasma membrane (Okazaki et al., 2015) and its catalytic activity was confirmed in vitro (Stevenson-Paulik et al., 2003). Thus, it is a prime candidate for producing PI4P at the plasma membrane. However, PI4Kα1 is a soluble protein with no protein–lipid interaction domains or anchoring mechanism characterized in planta. How PI4Kα1 is recruited and contributes to the architecture of the plasma membrane are open questions.

Here, we established that PI4Kα1 belongs to a 4-subunit complex composed of proteins from the NO POLLEN GERMINATION (NPG), HYCCIN-CONTAINING (HYC), and EFR3 OF PLANTS (EFOP) protein families. Using fluorescent protein tagging, immunolocalization, and subcellular fractionation, we confirmed the presence of the PI4Kα1 complex at the plasma membrane. Furthermore, we show that *pi4kα1* loss-of-function leads to full male sterility. The mutant pollen grains collapse and display abnormal cell walls. Knockout of any subunits of the PI4Kα1 complex mimics *pi4kα1* pollen lethality. Moreover, we established that the four subunits of the complex are essential for *PI4Kα1* function. Using mutant variants and chimeric constructs, we showed that the function of this complex is to target PI4Kα1 to the plasma membrane via lipid anchors. Finally, we observed that this heterotetrameric complex is not homogenously present on the plasma membrane but enriched in nanodomains. Although all the subunits of the complex are peripheral proteins and lack a transmembrane domain, they show very little lateral mobility at the plasma membrane. These results suggest that PI4Kα1 is not localized homogeneously at the plasma membrane but rather accumulates in distinct hotspots at the inner leaflet of the plasma membrane. Consequently, the targeting of this lipid kinase by a multiprotein scaffold might allow its precise spatiotemporal recruitment in order to maintain the proper electrostatic landscape of plant cell membranes.

## Results

### PI4Kα1 is a soluble protein with a potential lipid-binding domain

To determine how PI4Kα1 is recruited to the plasma membrane, we first analyzed its protein sequence in silico. Using TMHMM Server v. 2.0, no transmembrane helices could be predicted in the PI4Kα1 protein sequence suggesting that PI4Kα1 is a soluble cytosolic protein. We then looked for lipid binding domains. Indeed, a pleckstrin homology (PH) domain was previously reported in its C-terminal, upstream from the catalytic domain (Stevenson et al., 1998; Xue et al., 1999; Stevenson-Paulik et al., 2003). This PH domain was first thought to localize PI4Kα1 at the plasma membrane through interaction with anionic phospholipids but its role is debated (de Jong and Munnik, 2021). Fat blot experiments showed affinity of the putative PH domain for PI4P and to a lesser extent for PI(4,5)P_2_ (Stevenson et al., 1998; Stevenson-Paulik et al., 2003). However, no experiment in planta validates this result and using the Simple Modular Architecture Research Tool (SMART) software, we were not able to retrieve the PH domain. Because of the lack of a predicted domain (except for the kinase domain), we decided to consider other targeting mechanisms involving possible protein partners. Indeed, protein targeting to a membrane can be multifactorial and may require coincidence binding of lipids and protein partners.

### PI4Kα1 interacts with NO POLLEN GERMINATION proteins

**Figure 1:**
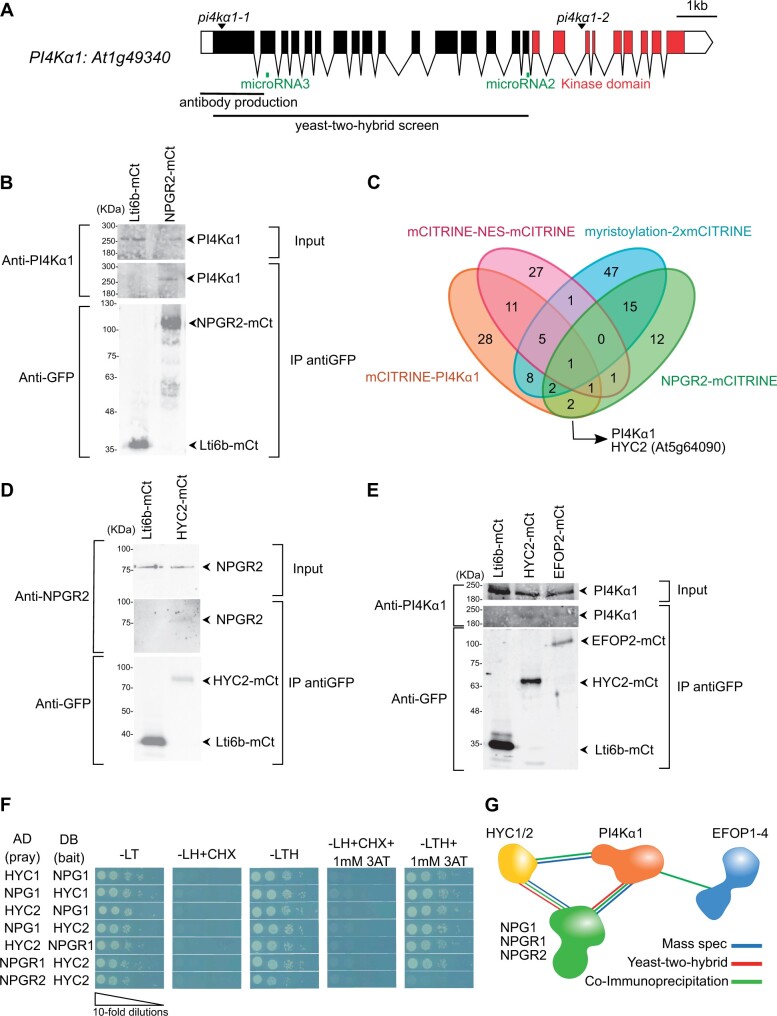
PI4Kα1 interacts with proteins from the NPG, HYC, and EFOP families. A, Schematic representation of *P*I4Kα*1* (*At1g49340*) gene. Boxes and lines represent exons and introns, respectively. The kinase domain is shown in red. The T-DNA positions of the *pi4k*α*1-1* and *pi4k*α*1-2* alleles are indicated. Parts of the protein used for the yeast-two-hybrid screen and the antibody production are also shown. The regions targeted by microRNA#2 and #3 are indicated in green. B, Co-IP of PI4Kα1 with NPGR2. Arabidopsis transgenic plants overexpressing NPGR2–mCITRINE (NPGR2–mCt) or Lti6b–mCITRINE (Lti6b–mCt) were used for IP using anti-GFP beads. Immunoblots used anti-PI4Kα1 (upper) and anti-GFP (lower). C, Venn diagram of proteins identified by mass spectrometry from IP of mCITRINE–PI4Kα1, NPGR2–mCITRINE, mCITRINE–NES–mCITRINE, and myristoylation–2x-mCITRINE. D, Co-IP of NPGR2 with HYC2. Arabidopsis transgenic plants overexpressing HYC2–mCITRINE (HYC2-mCt) or Lti6b–mCITRINE were used for IP using anti-GFP beads. Immunoblots used anti-GFP (lower) and anti-NPGR2 (upper). E, Co-IP of PI4Kα1 with EFOP2 and HYC2. Arabidopsis transgenic plants overexpressing EFOP2–mCITRINE (EFOP2–mCt), HYC2–mCITRINE, or Lti6b–mCITRINE were used for IP using anti-GFP beads. Immunoblots used anti-PI4Kα1 (upper) or anti-GFP (lower). F, Yeast-two hybrid assay of HYC1 with NPG1, HYC2 with NPG1 or NPGR1 and NPGR2. Indicated combinations of interactions between HYCCIN-CONTAINING and NPG proteins were assessed by growth on plates with yeast growth media lacking Leu, Trp, and His (-LTH). Yeast growth on plates lacking Leu and Trp (-LT) shows the presence of the bait and prey vectors. The absence of growth when cycloheximide was added (+CHX) shows the absence of auto-activation of the DB vectors. The addition of 3-amino-1,2,4-triazol (+3AT) shows the strength of the interaction. G, Summary of experiments showing interactions among PI4Kα1, NPG, HYC, and EFOP2 proteins.

To investigate this last hypothesis, we screened for PI4Kα1–protein partners. We performed a yeast-two-hybrid screen with the large N-terminal part of PI4Kα1 (1–1468 aa; Figure 1A). We recovered 267 in frame clones, which corresponded to 48 different proteins. Among them, the screen revealed interactions between PI4Kα1 and the three members of a protein family called NPG: NPG1 (*At2g43040*), NPG-Related 1 (NPGR1–*At1g27460*), and NPGR2 (*At4g28600*; Golovkin and Reddy, 2003). In the screen, we retrieved 39 clones (7 independent clones) for NPG1, 32 clones (6 independent clones) for NPGR1 and 2 clones (1 independent clone) for NPGR2. The clones from the NPG family corresponded to about 30% of the total clones obtained from the screen, suggesting that they were over-represented.

NPG proteins contain tetratricopeptide repeat (TPR) motifs that are protein–protein interaction motifs. In the yeast-two-hybrid screen, the selected interaction domain identified for NPG1, NPGR1, and NPGR2 correspond to the C-terminal part of the proteins (aa 444–704 for NPG1; 501–694 for NPGR1; and 414–739 for NPGR2). This is also the part of the sequence that contains the highest density of predicted TPR motifs suggesting that the interaction between PI4Kα1 and NPGs could be mediated by the C-terminal TPR motifs.

Because all three members of the NPG family interacted with PI4Kα1 in yeast-two hybrids and given the high degree of identity and similar architecture of the three proteins, we decided to focus on one member of the family to confirm the interaction in planta. We guided this choice based on the RNAseq expression data compiled in the eFP browser (https://bar.utoronto.ca/efp/cgi-bin/efpWeb.cgi). We chose *NPGR2*, as it has a stronger and wider expression level than the other members of the NPG family. Indeed, *NPG1* was predicted to be specifically expressed in the pollen, while *NPGR1* expression matched that of *NPGR2* but was predicted to be much weaker.

To confirm the interaction between NPGR2 and PI4Kα1, we produced stable transgenic lines expressing *UBQ10_pro_:NPGR2-mCITRINE*. We raised antibodies against the native PI4Kα1 (residues 1–344 of PI4Kα1). In immnoblot, the antibody recognized PI4Kα1 around the expected size (225 kDa) and the tagged version of PI4Kα1 with mCITRINE and 2xmCHERRY slightly higher (Supplemental Figure 1A and Supplemental Table S1). We immunoprecipitated NPGR2-mCITRINE or the plasma membrane protein Lti6b–mCITRINE as control using anti-GFP antibodies and probed whether they could co-immunoprecipitate PI4Kα1, using our native antibody. We efficiently immunoprecipitated NPGR2–mCITRINE or Lti6b–mCITRINE, but PI4Kα1 was only co-immunoprecipitated with NPGR2–mCITRINE (Figure 1B). Together, these experiments suggest that PI4Kα1 can interact in yeast with the C-terminus of all three members of the NPG family and is at least found in complex with NPGR2 in planta.

### NPG proteins interact with HYCCIN-CONTAINING proteins

Next, we asked whether the PI4Kα1 and NPG proteins could interact with additional protein partners. To this end, we used the lines expressing mCITRINE–PI4Kα1 and NPGR2–mCITRINE to perform immunoprecipitation (IP) followed by mass spectrometry analyses. Two lines expressing membrane-associated (myristoylation–2xmCITRINE) and nuclear-excluded (mCITRINE–NES–mCITRINE) proteins were also used as generic controls for plasma membrane and cytosolic proteins, respectively. In the NPGR2 IP, we found PI4Kα1, further confirming that these two proteins are present in the same complex in plants. Only one common protein was found in both NPGR2 and PI4Kα1 IPs but excluded from the two controls (Figure 1C). This protein was coded by the *At5g64090* locus and contains a HYCCIN domain. The Arabidopsis genome encodes only two proteins with a HYCCIN domain subsequently called HYCCIN-CONTAINING1 (HYC1, *At5g21050*) and HYCCIN-CONTAINING2 (HYC2, *At5g64090*).

*HYC2* is broadly expressed, while the *HYC1* expression is restricted to pollen according to the eFP browser data set (https://bar.utoronto.ca/efp/cgi-bin/efpWeb.cgi). Hence, we chose to test whether HYC2 interacted with PI4Kα1 and NPGR2 in sporophytic tissues. To this end, we raised *UBQ10::HYC2-mCITRINE* expressing lines and successfully isolated antibodies raised against NPGR2 (residues 1–273 of NPGR2). The expected size of NPGR2 is 82 kDa. The antibody recognized a band at ∼80 kDa that is not present in *npgr2-1* or *npgr2-3* knockout mutants or *npgr1 npgr2-1* double mutant (Supplemental Figure 1B and Supplemental Table S1). Moreover, the antibody recognized NPGR2–mCITRINE around 110 kDa, but did not recognize NPGR1-mCITRINE, indicating that the antibody specifically recognized NPGR2 (Supplemental Figure 1B). We found that NPGR2 co-immunoprecipitated with HYC2–mCITRINE but not Lti6b–mCITRINE (Figure 1D). Similarly, PI4Kα1 also co-immunoprecipitated with HYC2–mCITRINE but not with Lti6b–mCITRINE (Figure 1E). Next, we used yeast-two hybrid to check whether the two HYC family members may directly interact with PI4Kα1/NPGs (Figure 1F). We found that the two isoforms that are pollen specific, HYC1 and NPG1, interacted in yeast. Likewise, HYC2 interacted with NPG1, NPGR1, and NPGR2 in yeast. Together, our results indicate that HYC family members directly interact with NPG proteins. These data suggest that HYC2 is present in complex with PI4Kα1 and with NPGR2 in the Arabidopsis sporophyte, while HYC1/NPG1/PI4Kα1 could form a similar complex in the male gametophyte.

### A structural modeling approach suggests that PI4Kα1, NPG, and HYC form a heterotrimeric complex in plants

In human, Family With Sequence Similarity 126 (FAM126) has a HYCCIN domain and is part of a complex containing PI4KIIIα (Baskin et al., 2016; Dornan et al., 2018). In this complex, TETRATRICOPEPTIDE REPEAT PROTEIN 7 (TTC7) bridges together PI4KIIIα and FAM126 (Wu et al., 2014; Baskin et al., 2016; Lees et al., 2017; Dornan et al., 2018). While no HYCCIN containing proteins are found in yeast, the plasma membrane PI4-kinase, Stt4p also forms a complex containing Ypp1p, which, like NPG and TTC7 proteins, contains TPR motives (Baird et al., 2008; Nakatsu et al., 2012; Wu et al., 2014a). Based on these information, we hypothesized that NPGs and HYCs in plants could be functional homologs of TTC7/Ypp1p and FAM126, respectively.

**Figure 2.**
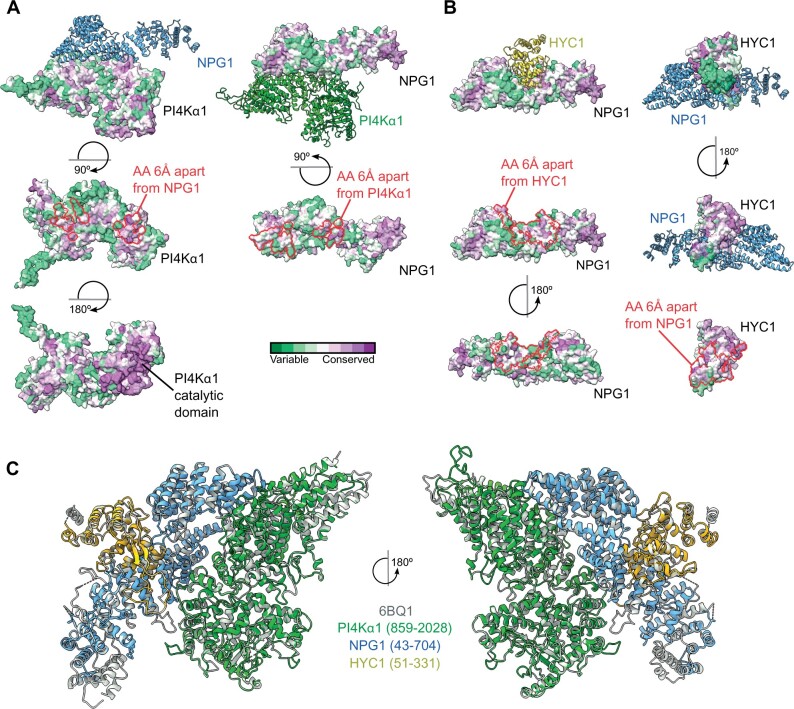
Template-based modeling and protein–protein docking suggest that the plant PI4Kα1 forms a stable heterotrimeric complex. A, Heterodimer formed by PI4Kα1 (green) and NPG1 (blue) as calculated by a hybrid docking approach using the HDOCK algorithm. Analysis of conserved amino acid residues was performed utilizing the Consurf server and mapped on the solvent-excluded surface of each protein. Calculated protein–protein interfaces indicated by red lines are formed by highly conserved amino acid residues. B, Heterodimer formed by NPG1 (blue) and HYC1 (yellow) as calculated by a hybrid docking approach using the HDOCK algorithm. Analysis of conserved amino acid residues was performed utilizing the Consurf server and mapped on the solvent-excluded surface of each protein. Calculated protein–protein interfaces indicated by red lines are formed by highly conserved amino acid residues. C, Comparison of the experimental structure of the human PI4KIIIα complex (gray, PDB code 6BQ1) with the heterotrimeric Arabidopsis PI4Kα1 complex obtained using template-based modeling and protein–protein docking.

The structure of the human trimeric complex formed by PI4KIIIα–TTC7–FAM126 has been determined by cryo-electron microscopy (Baskin et al., 2016; Lees et al., 2017; Dornan et al., 2018). We thus decided to use a templated-based modeling approach together with protein–protein docking to analyze the conservation of the overall structure of individual subunits and of their respective binding interface in particular. Using the structure of human PI4KIIIα, TTC7, and FAM126 as templates, we modeled the structure of PI4Kα1 (aa 859–2,028), NPG1 (aa 43–704), and HYC1 (aa 51–331) from Arabidopsis. Using this approach, we obtained a highly confident structural model for each Arabidopsis subunit (Supplemental Figure 2, A–C). We next predicted binding interfaces of the heterodimer composed of PI4Kα1–NPG1 and NPG1–HYC1 by a hybrid docking method using the HDOCK algorithm (Figure 2, A and B). We found that these interfaces are formed by highly conserved amino acids, suggesting that they are structurally important and supporting their potential key roles in the formation of the PI4Kα1–NPG1 and NPG1–HYC1 dimers. Of note, a majority of amino acid residues forming the PI4Kα1-binding interface of NPG1 (aa 585–655) corresponded to the selected interaction domain identified experimentally in the yeast two hybrid screen (aa 444–704; Figure 2A). Finally, we compared the overall experimental structure of the human PI4KIIIα complex with the model of the Arabidopsis PI4Kα1 complex obtained using the template-based modeling and protein–protein docking approach (Figure 2C). The structural comparison between the two structures revealed a highly similar arrangement of both complexes, again supporting the notion that Arabidopsis PI4Kα1–NPG1–HYC1 are structural homologs of human PI4KIIIα–TTC7–FAM126.

The modeling approach together with interaction data suggest that PI4Kα1, HYC, and NPG proteins form a stable heterotrimeric complex in planta, which is likely equivalent to its metazoan counterpart.

### EFOPs proteins are part of the PI4Kα1–NPG–HYC complex

In human cells and yeast, the PI4-kinase complex contains an additional subunit called EFR3/Efr3p (Baird et al., 2008; Nakatsu et al., 2012; Wu et al., 2014; Baskin et al., 2016; Lees et al., 2017; Dornan et al., 2018). We thus searched whether EFR3 homologs could exist in Arabidopsis using blast and sequence alignments. We found four potential candidates that we named EFOPs: EFOP1 (*At5g21080*), EFOP2 (*At2g41830*), EFOP3 (*At1g05960*), and EFOP4 (*At5g26850*).

Yeast Efr3p is a rod-shaped protein made of ARMADILLO-(ARM) and HEAT-like repeats. ARM and HEAT repeats are difficult to be distinguished bioinformatically, but all four EFOP proteins belong to the ARM-repeat super-family, which includes both ARM and HEAT repeat containing proteins (Wu et al., 2014a). In *Marchantia polymorpha*, mutants for *MpPI4K*α*1* (the homolog of *PI4K*α1*)* and *MpSRI4*, the homolog of *EFOP2*, display short rhizoids, suggesting that they could act in the same pathway and/or protein complex (Honkanen et al., 2016). In addition, based on RNAseq data, *EFOP2* have a rather large pattern of expression. Thus, we decided to concentrate on EFOP2 to test whether it is indeed present in the sporophytic PI4Kα1/NPGR2/HYC2 complex. To this end, we raised *UBQ10_pro_:EFOP2-mCITRINE* transgenic lines and immunoprecipitated EFOP2–mCITRINE and Lti6b–mCITRINE using an anti-GFP antibody. We found that PI4Kα1 co-immunoprecipitated with EFOP2–mCITRINE while it did not with Lti6b-mCITRINE (Figure 1E), suggesting that EFOP2 may belong to the PI4Kα1/NPGR2/HYC2 complex.

The summary of these interactions suggests that PI4Kα1 is part of an heterotetrameric complex in which NPG proteins may act as a scaffold that bridges EFOP, HYC, and PI4Kα1 proteins together (Figure 1G).

### *pi4kα1* mutants produce nonviable shriveled pollen grains with a thick cell wall

Next, we took a genetic approach to confirm whether NPG, HYC, and EFOP family members indeed may function together with PI4Kα1 in planta. To this end, we isolated single mutants for all the genes encoding for a subunit of the PI4Kα1 complex (Supplemental Table S2). We started our analysis with *PI4Kα1* because it is the catalytic subunit and it is present as a single-copy gene in the Arabidopsis genome for which we isolated two T-DNA insertion alleles. The first allele (*pi4k*α*1-1; GK_*502D11) had an insertion in the first exon, while the second insertion (*pi4k*α*1-2;* FLAG_275H12) was in the 20th intron (Figure 1A). T-DNAs of the first and second allele possess a sulfadiazine and glufosinate resistance gene, respectively. We failed to obtain homozygous mutant plants for both alleles. The segregations obtained after self-fertilization of heterozygous plants were 38% of sulfadiazine-resistant seedlings for *pi4kα1-1* and 9% of glufosinate-resistant seedlings for *pi4k*α*1-2* (Table 1). Because <50% of the progeny of self-fertilized *pi4kα1* alleles were resistant, these segregations indicated a likely gametophyte phenotype, which might explain the absence of homozygous *pi4kα1* mutant. To address whether the *pi4kα1* phenotype could be caused by the female and/or male gametophyte, we performed reciprocal crosses, using *pi4kα1^+/−^* and the wild-type as either male or female. For both alleles, we recovered 0% resistant plants when *pi4kα1^+/−^* was used as the male, indicating no transmission of the mutation via the pollen and thus complete male sterility (Table 2). When *pi4kα1*^+/^^*−*^ was used as female, we obtained 39% and 9% of resistant plants for each allele (Table 2). This result shows that the *pi4kα1* mutation did not fully impair the transmission through the female gametophyte but led to a partial distortion of the segregation.

**Table 1.** Segregation analyses of the indicated self-fertilized heterozygous mutants

Segregation						
Gene	Allele	Cross		Percentage of resistant plants	Percent expected	*n*
		Female	Male			
PI4Kα1	*pi4kα1-1*	*pi4kα1-1 ^+/−^*	*pi4kα1-1^+/−^*	38.2	75	418
	*pi4kα1-2*	*pi4kα1-2^+/−^*	*pi4kα1-2^+/−^*	9.4	75	359
NPG1	*npg1-1*	*npg1-1^+/−^*	*npg1-1^+/−^*	50.3	75	296
	*npg1-2*	*npg1-2^+/−^*	*npg1-2^+/−^*	32	75	504
HYC1	*hyc1*	*hyc1^+/−^*	*hyc1^+/−^*	38.2	75	128
HYC2	*hyc2-2*	*hyc2-2^+/−^*	*hyc2-2^+/−^*	59.9	75	133
	*hyc2-3*	*NO RESISTANCE*				

*n* represents the number of seedlings analyzed.

**Table 2.** Segregation analyses of reciprocal crosses between the wild-type and the indicated mutants

Reciprocal crosses						
Gene	Allele	Cross		Percentage of resistant plants	Percent expected	*n*
		Female	Male			
*PI4Kα1*	*pi4kα1-1*	*pi4kα1-1^+/−^*	Col0	39	50	424
		Col0	*pi4kα1-1^+/−^*	0	50	268
	*pi4kα1-2*	*pi4kα1-2^+/−^*	Col0	9.3	50	106
		Col0	*pi4kα1-2^+/−^*	0	50	293
*NPG1*	*npg1-1*	*npg1-1^+/−^*	Col0	40.6	50	214
		Col0	*npg1-1^+/−^*	0	50	163
	*npg1-2*	*npg1-2^+/−^*	Col0	30.8	50	26
		Col0	*npg1-2^+/−^*	0	50	111
*HYC1*	*hyc1*	*hyc1^+/−^*	Col0	47	50	185
		Col0	*hyc1^+/−^*	0	50	58
*HYC2*	*hyc2-2*	*hyc2-2^+/−^*	Col0	29.6	50	54
		Col0	*hyc2-2^+/−^*	29.8	50	47
	*hyc2-3*	*NO RESISTANCE*				
*ELP3 ELP4*	*elp3-1 elp4-2*	*elp3-1^+/−^ elp4-2^−/−^*	Col0	55.7	50	97
		Col0	*elp3-1^+/−^ elp4-2^−/−^*	2.2	50	45
	*elp3-2 elp4-2*	*elp3-2^+/−^ elp4-2^−/−^*	Col0	32.4	50	68
		Col0	*elp3-2^+/−^ elp4-2^−/−^*	0	50	31
	*elp3-1 elp4-4*	*elp3-1^+/−^ elp4-4^−/−^*	Col0	48.1	50	27
		Col0	*elp3-1^+/−^ elp4-4^−/−^*	ND	50	ND
	*elp3-2 elp4-4*	*elp3-2^+/−^ elp4-4^−/−^*	Col0	50	50	12
		Col0	*elp3-2^+/−^ elp4-4^−/−^*	0	50	6

*n* represent the number of seedlings analyzed.

**Figure 3:**
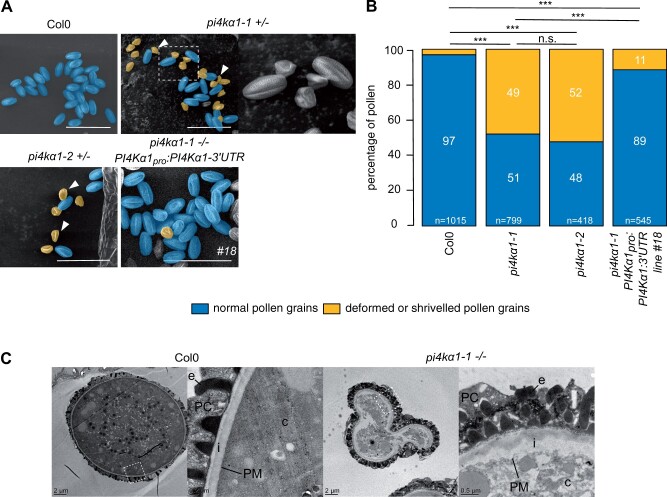
*PI4Kα1* loss-of-function leads to pollen lethality. A, Scanning electron microscope micrograph of pollen grains from Col-0, self-fertilized *pi4kα1-1* heterozygous plants, self-fertilized *pi4kα1-2* heterozygous plants, and self-fertilized *pi4kα1-1* homozygous plants expressing *PI4Kα1_pro_:PI4Kα1-3′-UTR* (insertion no. 18). Shriveled pollen grains are pseudocolored in yellow and normal pollen grains are pseudocolored in blue. Close-up is shown for *pi4kα1-1* pollen on the right. Scale bar: 50 µm. B, Quantification of the percentage of normal (blue) versus deformed/shriveled (yellow) pollen grains from Col-0, self-fertilized *pi4kα1-1* heterozygous plants, self-fertilized *pi4kα1-2* heterozygous plants, and self-fertilized *pi4kα1-1* homozygous plants expressing *PI4Kα1_pro_:PI4Kα1-3′UTR* (insertion no. 18). *n* indicates the number of pollens counted for each genotype. Statistics used chi-square test. n.s, non-significant; ***P < 0.001. C, Observation of Col-0 and *pi4kα1-1* shriveled pollen grains by transmission electron microscopy. Right parts show close-up of the region indicated on the left part. c, cytosol; PM, plasma membrane; i, intine; e, exine; PC, pollen coat.

Next, we observed *pi4kα1* pollen grains using scanning electron microscopy (SEM) to test whether they showed morphological defects (Figure 3A). For both alleles, half of the pollen grains were shriveled and likely not able to germinate explaining the absence of transmission of the mutation through the pollen (Figure 3, A and B). However, using Alexander staining, we observed that the *pi4kα1-1* pollens were still alive (Supplemental Figure 3B). 4′,6-diamidino-2-phénylindole (DAPI) staining also revealed the presence of the vegetative nucleus and the two sperm cell nuclei indicating that meiosis likely occurred normally (Supplemental Figure 3C). Further analysis by transmission electron microscopy showed that the *pi4kα1-1* pollen grains displayed an abnormally thick intine layer (Figure 3C).

The reintroduction of a wild-type copy of *PI4Kα1* under the control of its own promoter in the *pi4kα1-1* background fully complemented the *pi4kα1-1* lethality as shown by the possibility to obtain homozygous mutant plants (three independent complemented lines, see Supplemental Figure 3A**)**. In addition, self-fertilized *pi4kα1-1^−/−^; PI4Kα1_pro_:PI4Kα1* plants showed a low number of shriveled pollen grains, comparable to control plants, indicating that a wild-type copy of *PI4Kα1* is required for transmission through the male gametophyte and normal pollen morphology (Figure 3, A and B). Together, these results show that *PI4Kα1* is an essential regulator of pollen development, and in particular its cell wall deposition.

### Disturbing subunits of the PI4Kα1 complex mimics *pi4kα1* pollen phenotypes

Next, we isolated single mutants for all the genes encoding NPG, HYC, and EFOP subunits to examine whether they would recapitulate the *pi4kα1* loss-of-function phenotype (Supplemental Table S2).

**Figure 4:**
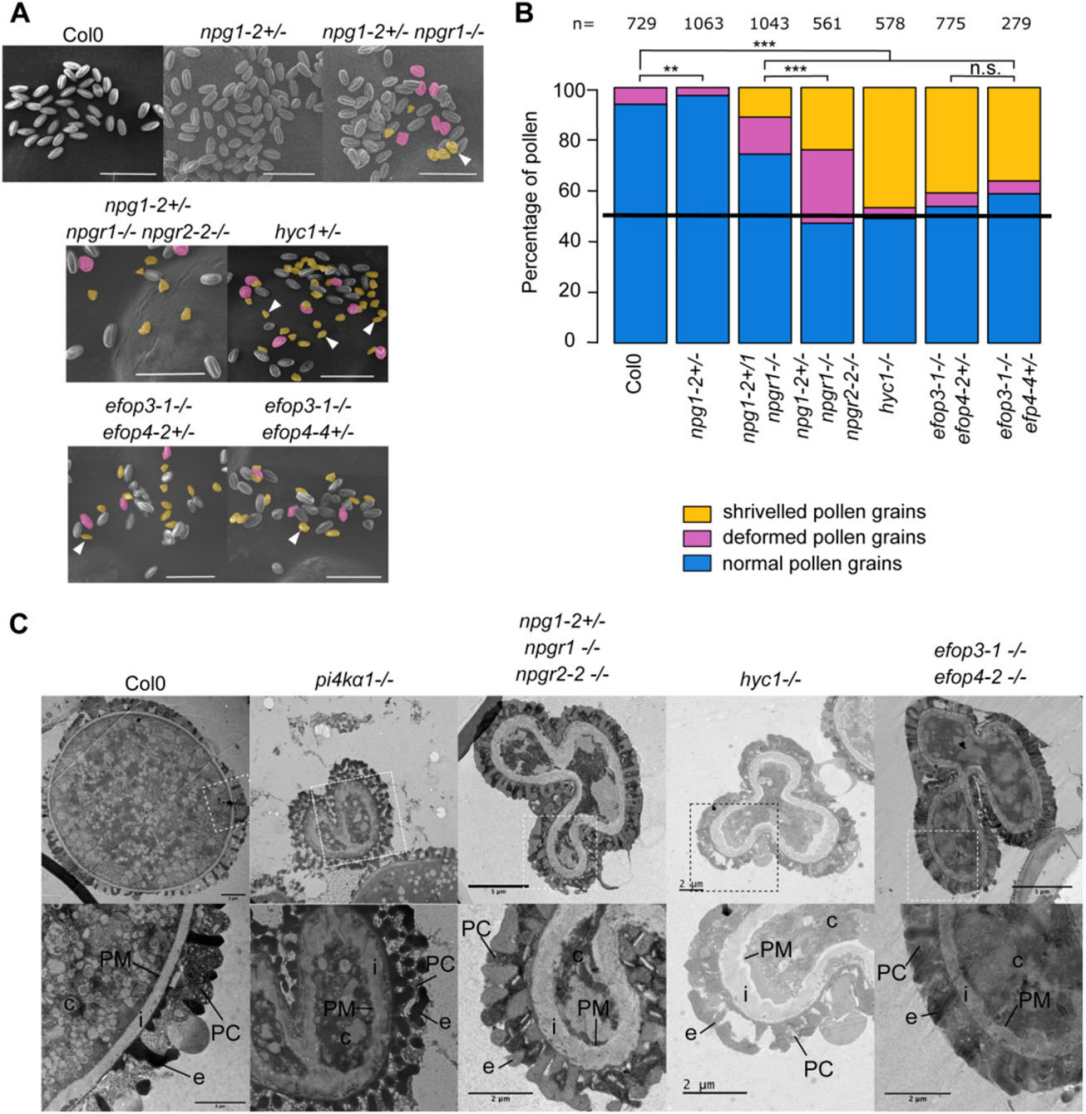
*NPG*, *HYC*, and *EFOP* mutations recapitulate the *pi4kα1* gametophytic phenotype including no male transmission and shriveled pollen grain with a thick cell wall. A, Pollen grains observed by scanning electron microscopy of self-fertilized Col-0, *npg1-2^+/−^*, *npg1-2^+/−^ npgr1^−/−^*, *npg1-2^+/−^ npgr1^−/−^ npgr2-2^−/−^*, *hyc1^+/−^*, *efop3-1^−/−^ efop4-2^+/−^*, and *efop3-1^−/−^ efop4-4^+/−^* plants. Deformed pollens and shriveled pollens are pseudocolored in yellow and magenta, respectively. Scale bar: 50 µm. B, Percentage of normal (blue), deformed (magenta) and shriveled (yellow) pollen grains from self-fertilized Col-0, *npg1-2^+/−^, npg1-2^+/−^ npgr1^−/−^, npg1-2^+/−^ npgr1^−/−^ npgr2-2^−/−^*, *hyc1^+/−^*, *efop3-1^−/−^ efop4-2^+/−^*, and *efop3-1^−/−^ efop4-2^+/−^* plants. *n* indicates number of pollens counted for each genotype. Statistics used chi-square test. n.s, nonsignificant; ***P < 0.001. C, Observation of pollen grains from self-fertilized Col-0, *pi4kα1-1, npg1-2^+/−^ npgr1^−/−^ npgr2-2^−/−^*, *hyc1*, and *efop3-1^−/−^ efop4-2^−/−^* by transmission electron microscopy. Lower part shows close-up of region indicated on upper part. c, cytosol; PM, plasma membrane; i, intine; e, exine; PC, pollen coat.

The *npg1* mutant was previously published as not being able to germinate giving its name NO POLLEN GERMINATION to the family (Golovkin and Reddy, 2003). We reproduced this result by characterizing two new T-DNA mutant alleles of *NPG1*. The self-progeny of *npg1-1^+/−^* and *npg1-2^+/−^* had a segregation rate of 50.3% and 32% resistant seedlings, respectively, indicating gamete lethality (Table 1). Reciprocal crosses confirmed their male sterility phenotype, with 0% of transmission of the mutation through the pollen, while the female gametophyte might be affected only for the second allele with a weak distortion of the segregation rate (Table 2). However, the observation of *npg1-1* and *npg1-2* pollen grains by SEM did not show any morphological defect, unlike *pi4kα1* pollens (Figure 4, A and B; Supplemental Figure 4, A and B). The reintroduction of *NPG1* fused with mCITRINE under the control of its own promoter complemented the male sterility in the *npg1-2* background, leading to *npg1-2* homozygous plants (Supplemental Figure 4C). Similarly, the expression of *NPGR2* fused to the mCITRINE under the control of the *NPG1* promoter also complemented *npg1-2* male sterility. These experiments indicate that NPGR2 can substitute for NPG1 function in pollen and that both NPG1–mCITRINE and NPGR2–mCITRINE fusion are fully functional (Supplemental Figure 4C).

Because, NPGR2 can substitute for NPG1 in pollen, we speculated that a certain degree of functional redundancy between NPG1, NPGR1, and NPGR2 or compensatory effects during pollen development could lead to the weaker phenotype of *npg1* pollen compared to *pi4kα1* pollen and thus explain why *npg1* pollen did not present a morphological defect by SEM. To test this hypothesis, we generated higher order mutant combinations within the *NPG* family (Supplemental Table S2). The *npg1-2^+/−^ npgr1^−/−^* mutant combination presented about 10% of *pi4kα1*-like shriveled pollen grains while *npgr1 npgr2-1* and *npgr1 npgr2-2* double homozygous mutants displayed about 25% of deformed (but not shriveled) pollen grains (Figure 4, A and B; Supplemental Figure 4, A and B). Finally, the *npg1-2^+/−^ npgr1^−/−^ npgr2-1^−/−^* and *npg1-2^+/−^ npgr1^−/−^ npgr2-2^−/−^* plants displayed about 35% and 50% of deformed and shriveled pollen grains, respectively. Furthermore, images in transmission electron microscopy showed similar thickening of the cell wall for *pi4kα1* and *npg1-2 npgr1 npgr2-2* shriveled pollen grains (Figure 4C). These data indicate that *npg* single/multiple mutants partially or fully mimic the *pi4kα1* pollen phenotype depending on the allelic combinations.

Next, we addressed the loss-of-function phenotypes of the *HYCCIN-CONTAINING* family members. The self-progeny of a *hyc1^+/−^* single mutant presented a segregation rate of around 50%, indicating gametophytic lethality (Table 1). As *HYC1* expression is restricted to pollen, we were expecting that the segregation bias was caused by defects of the male gametophyte. As anticipated, reciprocal crosses showed complete male sterility while the T-DNA transmission through the female gametophyte was not affected (Table 2). Observation of *hyc1^+/−^* pollen grains by SEM revealed that half of the pollen grains were shriveled (Figure 4, A and B). In addition, transmission electron microscopy also showed a thickening of the cell wall of the *hyc1* mutant pollen grains, which was similar to the phenotype observed for *pi4kα1-1* (Figure 4C). Finally, the male sterility, as well as the pollen morphological defects, was complemented by the reintroduction of *HYC1_pro_:HYC1-mCITRINE* (Supplemental Figure 4, A, B, and D). All together, these data show that the *hyc1* knockout phenotype fully mimics *pi4kα1* knockout regarding pollen development.

None of the *efop* single mutants presented any pollen morphological defects or distortion of segregation, likely because of redundancy between the four members of this family (Supplemental Figure 4B). We thus generated all the possible combinations of double mutants (Supplemental Figure 4B; Table 3). We were able to obtain *efop2 efop3* double homozygous mutants, suggesting no strong synthetic lethality. However, these mutants presented from 19% to 25% of shriveled pollen grains, resembling those of the *pi4kα1* and *hyc1* mutants, and from 43% to 65% of deformed pollens (resembling those of *npgr1 npgr2* double mutants). Thus, depending on the alleles, these double mutant combinations presented from 70% to 90% of abnormal pollens (Supplemental Figure 4, A and B). In addition, it was not possible to generate a *efop3 efop4* double homozygous mutant no matter the alleles used. Indeed, reciprocal crosses indicated 0% of transmission of the *efop3* mutant allele when *efop3^+/−^ efop4^−/−^* plants were used as male, revealing that *efop3 efop4* pollens were lethal (Table 2). SEM showed that about 45% of the pollen grains present an abnormal morphology (Figure 4, A and B; Supplemental Figure 4, A and B). Finally, the observation of *efop3-1 efop4-2* shriveled pollen grains by electron transmission microscopy revealed a thick cell wall similar to the phenotype observed for *pi4kα1-1* and *hyc1*. *efop2 efop3* and *efop3 efop4* double mutants mimic partially and fully *pi4kα1* and *hyc1* pollen phenotypes, respectively. Altogether, our genetic analyses indicate that all the protein classes in the putative PI4Kα1 complex are essential for the male gametophyte in Arabidopsis and that certain mutant combinations in the *NPG*, *HYC*, or *EFOP* families either fully (*hyc1^+/−^*, *efop3^+/−^ efop4^−/−^*) or partially (*npg1^+/−^*, *npg1^+/−^ npgr1^−/−^*, *npgr1^−/−^ npgr2^−/−^*, *npg1-2^+/−^ npgr1^−/−^ npgr2-2^−/−^*, *efop2^−/−^ efop3^−/−^*) mimic the *pi4kα1* phenotype. Thus, these proteins likely act together in plants, potentially as a single protein complex.

### Disturbing the PI4Kα1 complex results in various sporophytic phenotypes

**Figure 5:**
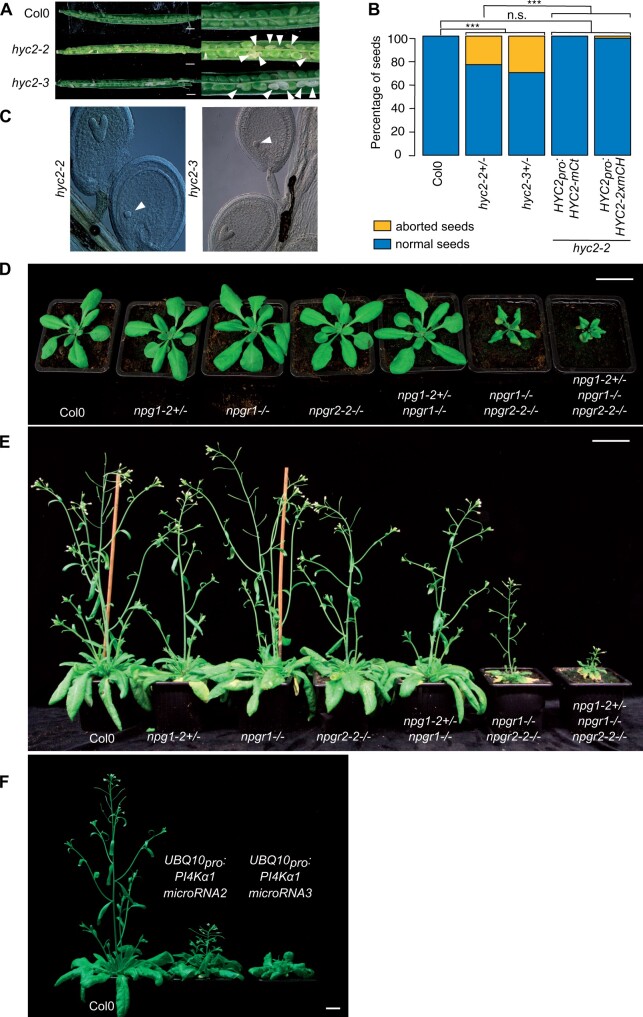
Mutations of the PI4Kα1 complex induce sporophytic phenotypes. A, Opened siliques of self-fertilized Col-0, *hyc2-2* and *hyc2-3* heterozygous mutant plants. White arrowheads indicate aborted seeds. B, Percentage of aborted seeds in Col-0, *hyc2-2^+/−^*, *hyc2-3^+/−^*, *hyc2-2^−/−^ HYC2_pro_:HYC2-mCITRINE* (insertion no. 10), and *hyc2-2^−/−^ HYC2_pro_:HYC2-2xmCHERRY* (insertion no. 11) siliques. The number of seeds counted is superior to 250 for each genotype. Statistics used chi-square test. n.s., nonsignificant; ***P < 0.001. C, Cleared seeds from *hyc2-2* and *hyc2-3* heterozygous mutant plants. White arrowheads indicate globular embryos that have stopped development. D, Twenty-seven-day-old Col-0, *npg1-2^+/−^*, *npgr1^−/−^*, *npgr2-2^−/−^*, *npg1-2^+/−^ npgr1^−/−^*, *npgr1^−/−^ npgr2-2^−/−^*, and *npg1-2^+/−^ npgr1^−/−^ npgr2-2^−/−^* plants. Scale bar: 2 cm. E, Forty-one-day-old Col-0, *npg1-2^+/−^*, *npgr1^−/−^*, *npgr2-2^−/−^*, *npg1-2^+/−^ npgr1^−/−^*, *npgr1^−/−^ npgr2-2^−/−^*, and *npg1-2^+/−^ npgr1^−/−^ npgr2-2^−/−^* plants. Scale bar: 2 cm. F, Thirty-eight-day-old Col-0 and plants expressing *microRNA2* and *microRNA3* against *PI4Kα1*. Scale bar: 2 cm.

While *HYC1* is specifically expressed in pollen and is male sterile, *HYC2* is predicted to be expressed in the sporophyte, which suggests that *HYC2* loss-of-function could lead to sporophytic phenotypes. We characterized two T-DNA alleles corresponding to two putative *hyc2* loss-of-function mutants. The segregation rate of *hyc2-2* heterozygous plants was 60% (Table 1). Moreover, it was not possible to retrieve homozygous plants in the self-progeny of both *hyc2-2* and *hyc2-3*. Reciprocal crosses indicated a transmission of the allele through the male and the female gametophytes even if a weak distortion could be observed in both cases (Table 2). Siliques from *hyc2-2* and *hyc2-3* heterozygous plants presented around 25% to 30% of aborted seeds (Figure 5, A and B; Supplemental Figure 5A). Observations of the embryos after clearing showed that in those siliques, some embryos stopped their development at the globular stage before degenerating (likely corresponding to homozygous *hyc2* mutant embryos) while the rest of the embryos pursued their development normally (likely corresponding to the wild-type and *hyc2^+/−^* embryos; Figure 5C). This phenotype was lost and homozygous mutant plants were obtained when HYC2-mCITRINE or HYC2-2xmCHERRY were reintroduced under the control of the *HYC2* promoter (Figure 5B;  Supplemental Figure 5, A and B). Thus, the loss of HYC2 leads to embryo lethality at the globular stage, suggesting that HYC1 is essential for the male gametophyte, while HYC2 is essential for embryogenesis. These results are consistent with the idea that the four-subunit PI4Kα1 complex is essential in plants beyond the development of the male gametophyte.

**Figure 6:**
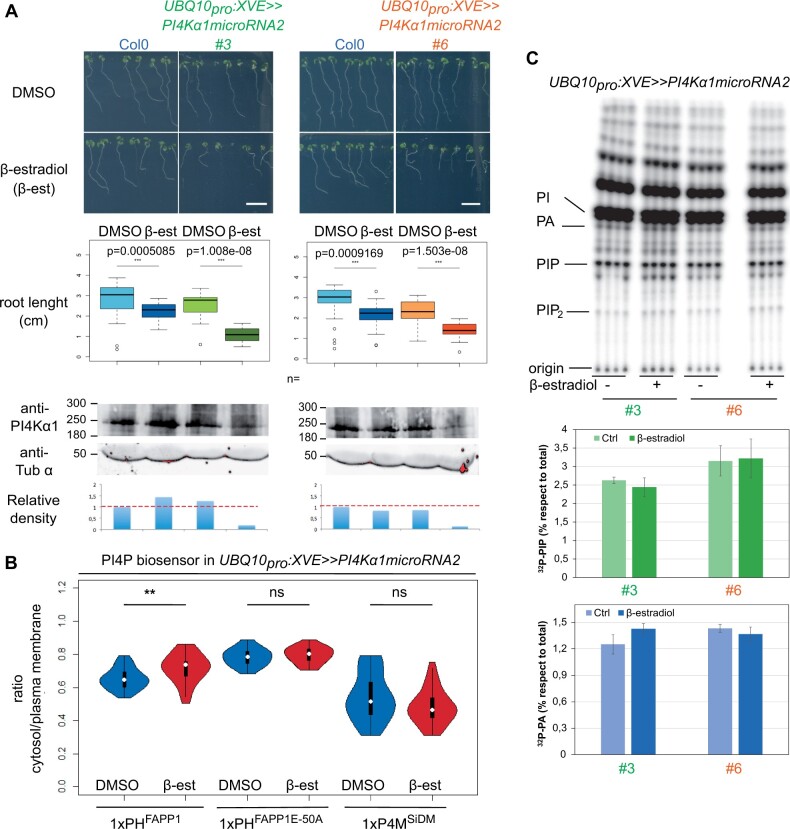
Inducible *PI4Kα1* knockdown impacts sporophytic development and has subtle effects on PI4P accumulation at the plasma membrane. A, Pictures and measures of the primary root of 9-day-old seedlings of Col-0 and seedlings expressing the inducible *microRNA2* against *PI4Kα1* on noninducible (DMSO) and inducible medium (5 μM β-estradiol, β-est) supplemented with sucrose for two independent insertions (#3 in green and #6 in orange). Scale bar: 1 cm. *n* indicates the number of seedlings measured. Whiskers correspond to the lower and upper quartile, the median and the minimum and maximum (excluding the outliers), respectively. Statistics were done using Wilcoxon test. Bottom, PI4Kα1 protein levels of 9-day-old Col-0 seedlings and seedlings expressing the inducible *microRNA2* against *PI4Kα1* on noninducible (DMSO) and inducible medium (5-μM β-estradiol) for two independent insertions (#3 and #6). Immunoblot used an anti-PI4Kα1 antibody and an anti-tubuline α (anti-Tub α) as control. The relative density of signal adjusted to the Col-0 DMSO condition is indicated. B, Cytosol/plasma membrane signal intensity ratio of PI4P biosensors mCITRINE-1xPH^FAPP1^ (P5Y), mCITRINE-1xPH^FAPP1-E50A^, and mCITRINE-P4M^SidM^ on epidermal root cells of seedlings expressing the inducible *microRNA2* against *PI4Kα1* on noninducible (DMSO) and inducible medium (5-μM β-estradiol). *n* indicates the number of seedlings measured. Three cells per seedling were measured. Statistics were done using Wilcoxon test. C, TLC analysis of lipid extract from 9-day-old ^32^P_i_-prelabeled seedlings expressing the inducible *microRNA2* against *PI4Kα1* on noninducible (DMSO) and inducible medium (5-μM β-estradiol) supplemented with sucrose for two independent insertions (3 and 6). ^32^P_i_ labeling was performed overnight (16–20 h), and quantification of ^32^P-levels in PIP and PA (as control) by phosphoimaging and calculated as percentage of total ^32^P-lipids. Each sample represents the extract of three seedlings and each treatment was performed in quadruplicate of which averages ± sd are shown.

The lethality of *pi4kα1* or *hyc* knockouts did not allow us to further study the role of PI4Kα1 at the cellular and developmental levels in plants. However, some combinations of *npg* mutants presented growth defect phenotypes. Indeed, *npgr1 npgr2-2* double mutant and *npg1^+/−^ npgr1^−/−^ npgr2-1^−/−^* presented a mild growth phenotype while *npg1^+/−^ npgr1^−/−^ npgr2-2^−/−^* was dwarf (Figure 5, D and E; Supplemental Figure 5, C and D). Reintroduction of *NPGR1_pro_:NPGR1-mCITRINE* was able to rescue the growth phenotype of the *npgr1 npgr2-2* double mutant (Supplemental Figure 5, E and F). This suggests that the PI4Kα1 complex is essential not only for pollen and embryo development but also for later developmental and growth processes. To confirm this hypothesis, we developed a knockdown strategy using a total of four independent artificial microRNAs targeted against *PI4Kα1*. Primary transformants expressing ubiquitously the artificial microRNAs numbers 2 and 3 showed strong growth defects (Figure 5F). The ubiquitous expression of the *PI4Kα1* microRNA lines 2 and 3 lead to phenotypes with variable strength. However, most of the plants died or were sterile, which confirmed the essential role of the PI4Kα1 complex not only in gametophytes but also for sporophytic development. Because plants constitutively expressing the microRNAs against *PI4Kα1* died, we next put the *artificial microRNA2*, which had the strongest phenotype when constitutively expressed, under the control of a β-estradiol-inducible ubiquitous promoter (*UBQ10_pro_:XVE*; Siligato et al., 2016). Induction of this *artificial microRNA2* led to a decrease in the amount of the PI4Kα1 protein and the corresponding seedlings exhibited shorter primary roots (Figure 6A).

With this tool in hand, we next evaluated the effect of *PI4Kα1* knockdown on the PI4P pool using several approaches. First, we studied the impact of PI4K*α1* knockdown on the localization of PI4P biosensors by calculating the ratio of fluorescence intensity between the cytosol and plasma membrane (Figure 6B). Upon induction, the cytosol/plasma membrane ratio of the PI4P sensor 1xPH^FAPP1^ (also known as P5Y, Simon et al., 2014) increased, although a significant amount of the sensor was still localized at the plasma membrane (Figure 6B). This result suggests that the amount of PI4P at the plasma membrane was reduced upon inducible *PI4Kα1* knockdown (Figure 6B). However, the cytosol/plasma membrane ratio remained unchanged for two additional PI4P biosensors, 1xPH^FAPP1-E50A^ and P4M (Simon et al., 2016). These two biosensors have a high affinity for the plasma membrane pool of PI4P, while 1xPH^FAPP1^ interacts with PI4P as well as with the TGN/EE-localized protein ARF1 (Simon et al., 2014, 2016). These results thus suggest that the decrease of PI4P at the plasma membrane is relatively modest. Indeed, it is not sufficient to perturb the subcellular targeting of pure PI4P-binding proteins to the plasma membrane (such as 1xPH^FAPP1-E50A^ and P4M), but it can impact the localization of coincident detectors such as 1xPH^FAPP1^ (which interacts both with PI4P and ARF1). Consistently, the amount of Phosphorus-32 labelled phosphatidylinositol monophosphate (^32^P-PIP) in lipid extracts of ^32^P_i_-prelabeled seedlings grown on β-estradiol was unchanged compared to the nontreated control (Figure 6C).

Together, these data confirm that PI4Kα1 acts at the plasma membrane in the production of PI4P. They also suggest that our *PI4Kα1* knockdown strategy is not sufficient to strongly impact the pool of PI4P at the plasma membrane. We reason that the remaining PI4Kα1 may sustain PI4P production in this compartment, although we cannot exclude the possibility that other enzymes participate in PI4P production upon *PI4Kα1* knockdown. Importantly, relatively subtle changes in PI4P amount at the plasma membrane (Figure 6B) already lead to visible developmental phenotypes (Figure 6A). This suggests that stronger depletion of PI4P at the plasma membrane would likely lead to lethality, as observed in the pollen grains and embryos of various PI4Kα1 complex mutants. Together, our results indicate that the PI4Kα1-dependent pool of PI4P at the plasma membrane is likely required for cellular life in plants, including during gametophyte, embryonic and post-embryonic development.

### The PI4Kα1 complex is associated with the plasma membrane

**Figure 7:**
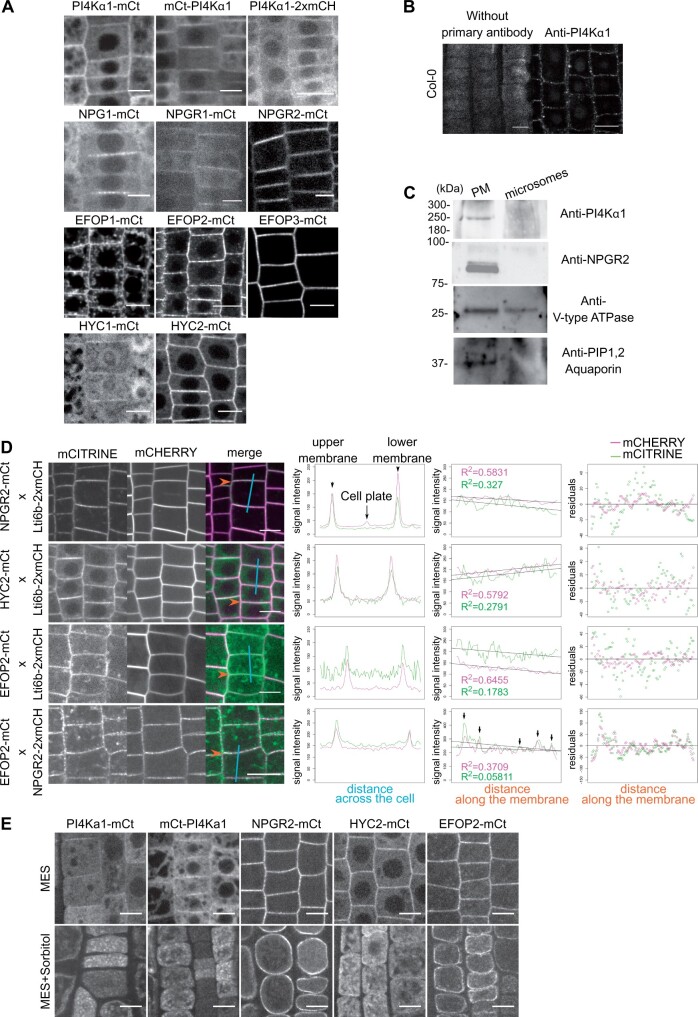
The PI4Kα1 complex localizes at the plasma membrane. A, Confocal images of PI4Kα1, NPG1, NPGR1, NPGR2, HYC1, HYC2, EFOP1, EFOP2, and EFOP3 fused to mCITRINE (mCt) or 2xmCHERRY under the control of the *UBQ10* promoter in root epidermal cells. Scale bar: 10 µm. B, Confocal images of PI4Kα1 using an anti-PI4Kα1 antibody in epidermal root cells on WT seedlings. Control background without primary antibody is shown. Scale bar: 10 µm. C, Immunoblot using anti-PI4Kα1, anti-NPGR2, anti-V-type ATPase, and anti-PIP1,2 aquaporin antibodies on plasma membrane and microsomal fractions from WT seedlings. D, Confocal images of seedlings co-expressing Lti6b-2×mCHERRY (under the control of the *2*×*35S* promoter), NPGR2–mCITRINE, HYC2–mCITRINE, or EFOP2–mCITRINE (under the control of the *UBQ10* promoter). Graphics on the left represent intensity of each signal across the cell along the cyan line. Graphics in the middle represent intensity of each signal along the membrane indicated by the orange arrow. Matching pic intensity is indicated by black arrows. Linear regression and adjusted R square for each signal are indicated. Graphics on the right represent residuals in y between signal along the membrane and linear regression. E, Confocal images of PI4Kα1-mCt, mCt-PI4Kα1, NPGR2-mCt, HYC2-mCt, EFOP2-mCt under the control of the *UBQ10* promoter in root epidermal cells in control condition (MES) and during plasmolysis (MES+Sorbitol). Scale bar: 10 µm.

To confirm the localization of PI4Kα1 at the plasma membrane, we first raised stable transgenic lines expressing PI4Kα1 tagged with mCITRINE either at its N-terminal or C-terminal ends and the red fluorescent protein 2xmCHERRY at the C-terminal end under the control of either its own promoter or the *UBQ10* promoter. Consistent with the hypothesis that PI4Kα1 acts at the plasma membrane, the three constructs mCITRINE–PI4Kα1, PI4Kα1–mCITRINE, and PI4Kα1–2xmCHERRY localized at the plasma membrane and in the cytosol in root epidermal cells and in pollen grains (Figure 7A;  Supplemental Figure 6). However, the introduction of *PI4Kα1_pro_:PI4Kα1-mCITRINE*, *PI4Kα1_pro_:mCITRINE-PI4Kα1*, or *PI4Kα1_pro_:PI4Kα1-2xmCHERRY* constructs in the *pi4kα1-1^+/−^* mutant background failed to complement the pollen lethality and we never recovered *pi4kα1-1^−/−^* plants (Supplemental Table S3). We used the same *PI4Kα1* promoter used for the rescue experiment with the untagged *PI4Kα1* (Figure 3, B and C; Supplemental Figure 3A), suggesting that PI4Kα1 fused with a fluorescent protein is nonfunctional. Expression of *PI4Kα1* fused to smaller tags (i.e. PI4Kα1-6xHA or Flag-PI4Kα1) also failed to complement *pi4kα1-1* (Supplemental Table S3).

To confirm the localization obtained with mCITRINE fusion, we used the antibodies against the native PI4Kα1 and performed whole mount immunolocalization in roots. Similar to the mCITRINE–PI4Kα1 and PI4Kα1–mCITRINE fusions, we observed again a signal at the plasma membrane (Figure 7B). To further confirm the preferential association of PI4Kα1 with the plasma membrane, we used cellular fractionation and PEG/Dextran phase partition of whole seedlings and compared the signal obtained on a purified plasma membrane or whole microsomal fractions. We confirmed the presence of proteins in the two fractions using an antibody against V-type ATPase. The purity of the plasma membrane fraction was evaluated with antibodies against the PIP1,2 aquaporin, a known plasma membrane resident protein (Figure 7C). When loading the same amount of protein in each fraction, this experiment revealed the presence of a band around 225 kDa, corresponding to PI4Kα1 in the plasma membrane fraction and only a very faint signal in the total microsomal fraction, showing that PI4Kα1 is enriched in the plasma membrane fraction (Figure 7C). Together, fluorescent fusion, immunolocalization, and cellular fractionation showed that PI4Kα1 is associated with the plasma membrane.

We next addressed the subcellular localization of NPG, HYC, and EFOP proteins at the plasma membrane. NPG1 was previously found to be an extracellular protein in pollen grains (Shin et al., 2014). In our hand, NPG1-mCITRINE, NPGR1-mCITRINE, and NPGR2-mCITRINE localized at the periphery of the cell in root meristem (Figure 7A). In addition, NPGR1–mCITRINE and NPGR2–mCITRINE were found at the plasma membrane in pollen grains (Supplemental Figure 6). To confirm this localization and make sure to distinguish between the plasma membrane and cell wall, we co-expressed NPGR2–mCITRINE with Lti6b–2xmCHERRY. We observed that the two signals perfectly colocalized, indicating that NPGR2 is present at the plasma membrane (Figure 7D). Furthermore, we performed plasmolysis of the epidermal root cell by addition of sorbitol. In this context, the plasma membrane detaches from the cell wall. We observed that the signal of NPGR2-mCITRINE remains at the plasma membrane and is not present in the cell wall (Figure 7E). Moreover, in immunoblot using an anti-NPGR2 antibody, NPGR2 was found to be enriched in the plasma membrane fraction compared to the microsomal fractions (Figure 7C).

Similarly, HYC1–mCITRINE, HYC2–mCITRINE, EFOP1–mCITRINE, EFOP2–mCITRINE, and EFOP3–mCITRINE expressed under the control of the *UBQ10* promoter were found at the plasma membrane in root epidermal cells (Figure 7A) and in pollen grains, with the exception of HYC2 and EFOP2, which were highly cytosolic in pollen grains (Supplemental Figure 6). In addition to the plasma membrane localization, we noticed that EFOP1 and EFOP2 were associated with intracellular compartments in epidermal root cells, which were more prominently labeled for EFOP1 than EFOP2.

Upon plasmolysis, PI4Kα1–mCITRINE, mCITRINE–PI4Kα1, HYC2–mCITRINE, and EFOP2–mCITRINE signals are found inside the cell and absent from the cell wall. EFOP2 remained associated with the plasma membrane while PI4Kα1 and HYC2 were delocalized to internal compartments (Figure 7E). In any case, all the protein classes in the putative PI4Kα1 complex are extrinsic proteins associated to some extent with the cytoplasmic leaflet of the plasma membrane in both the root meristem and mature pollen grain.

### The PI4Kα1 complex is present in plasma membrane nanodomains

**Figure 8:**
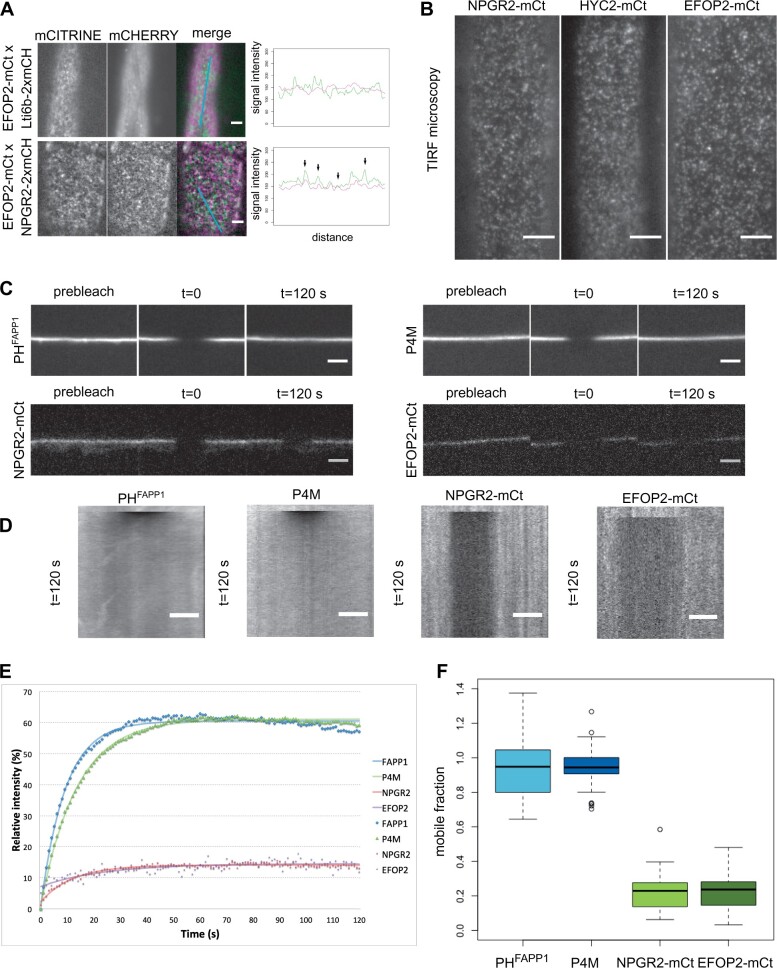
The PI4Kα1 complex localizes in highly static nanodomains at the plasma membrane. A, Confocal images of seedlings co-expressing Lti6b–2×mCHERRY (under the control of the *2x35S* promoter), NPGR2–mCITRINE, or EFOP2–mCITRINE (under the control of the *UBQ10* promoter). Graphics represent intensity of each signal across the cell along the cyan line. Black arrows indicate matching signals. Scale bar: 5 µm. B, Confocal images of TIRF microscopy of NPGR2-mCt, HYC2-mCt, and EFOP2-mCt. Scale bar: 5 µm. C, Confocal images of P4M, PH^FAPP1^, NPGR2-mCt, and EFOP2-mCt before photobleaching (prebleach) and 120 s after photobleaching. Scale bar: 5 µm. D, Kymographs along the membrane for the time lapse in (C). Scale bar: 5 µm. E, Graphic presenting the recovery of the signal intensity over time after bleaching. The number of zones measured is 37, 32, 30, and 13 for P4M, PH^FAPP1^, NPGR2-mCt, and EFOP2-mCt, respectively. The fitting curves are represented. F, Graphic presenting the mobile fraction of P4M, PH^FAPP1^, NPGR2-mCt, and EFOP2-mCt after 2 min post-bleaching taking in account the inherent bleaching due to imaging. Whiskers correspond to the lower and upper quartile, the median and the minimum and maximum (excluding the outliers), respectively.

Using confocal microscopy, we noticed that for several of the translational reporters of the PI4Kα1 complex, the signal at the plasma membrane was not continuous, raising the question of a possible subcompartmentalization of the proteins. This is notably the case for PI4Kα1–mCITRINE, NPG1–mCITRINE, NPGR2–mCITRINE, HYC2–mCITRINE, EFOP2–mCITRINE, and EFOP3–mCITRINE (Figure 7, A and D and Figure 8A). A similar discontinuous pattern at the plasma membrane was also evident from PI4Kα1 immunolocalization (Figure 7A). Using plants co-expressing Lti6b–2xmCHERRY and NPGR2–mCITRINE, HYC2–mCITRINE or EFOP2–mCITRINE, we observed that NPGR2–mCITRINE, HYC2–mCITRINE, and EFOP2–mCITRINE signals along the plasma membrane are less homogeneous than Lti6b, and accumulated in patches of high intensity (Figure 7D). We calculated the linear regression of each signal along the membrane and observed that the R square of NPGR2–mCITRINE, HYC2–mCITRINE, and EFOP2–mCITRINE is always smaller than the one of Lti6b–2cmCHERRY, indicating a higher dispersion of the intensity. In addition, plants co-expressing NPGR2–2xmCHERRY and EFOP2–mCITRINE show similar intensity pattern with signals partially localized along the plasma membrane (Figure 7D). Similarly, when we observed the plasma membrane in tangential sections, NPGR2–2xmCHERRY and EFOP2–mCITRINE subdomains were partially colocalized (Figure 8A). As control, EFOP2–mCITRINE containing plasma membrane domains did not colocalize with the mostly uniformed localization of Lti6b–2xmCHERRY (Figures 7D and 8A). In order to get a better axial resolution, we used total internal reflection fluorescence (TIRF) microscopy and confirmed that NPGR2–mCITRINE, HYC2–mCITRINE, and EFOP2–mCITRINE were present in nanodomains of the plasma membrane (Figure 8B).

### PI4Kα1 complex-containing nanodomains are static at the plasma membrane

Next, we investigated the lateral dynamics of the PI4Kα1 complex at the plasma membrane. To do so, we used fluorescence recovery after photobleaching (FRAP). After bleaching, the signal of NPGR2–mCITRINE, HYC2–mCITRINE, and EFOP2–mCITRINE did not recover after 2 min of acquisition (Figure 8, C–E; Supplemental Figure 7). In comparison, PI4P sensors (P4M and PH^FAPP1^) fluorescence recovered in less than a minute after bleaching. Accordingly, the mobile fraction calculated of NPGR2–mCITRINE and EFOP2–mCITRINE was low (around 20%), while the mobile fraction of the sensor reached 100% (Figure 8F). This indicates that if PI4P sensors are rapidly diffusing at the membrane, the PI4Kα1 complex is relatively static. Furthermore, the identical dynamics of NPGR2–mCITRINE, HYC2–mCITRINE, and EFOP2–mCITRINE further reinforce the notion that these subunits are part of a single protein complex in vivo.

### EFOPs localize at the plasma membrane via S-acylation lipid anchoring

**Figure 9:**
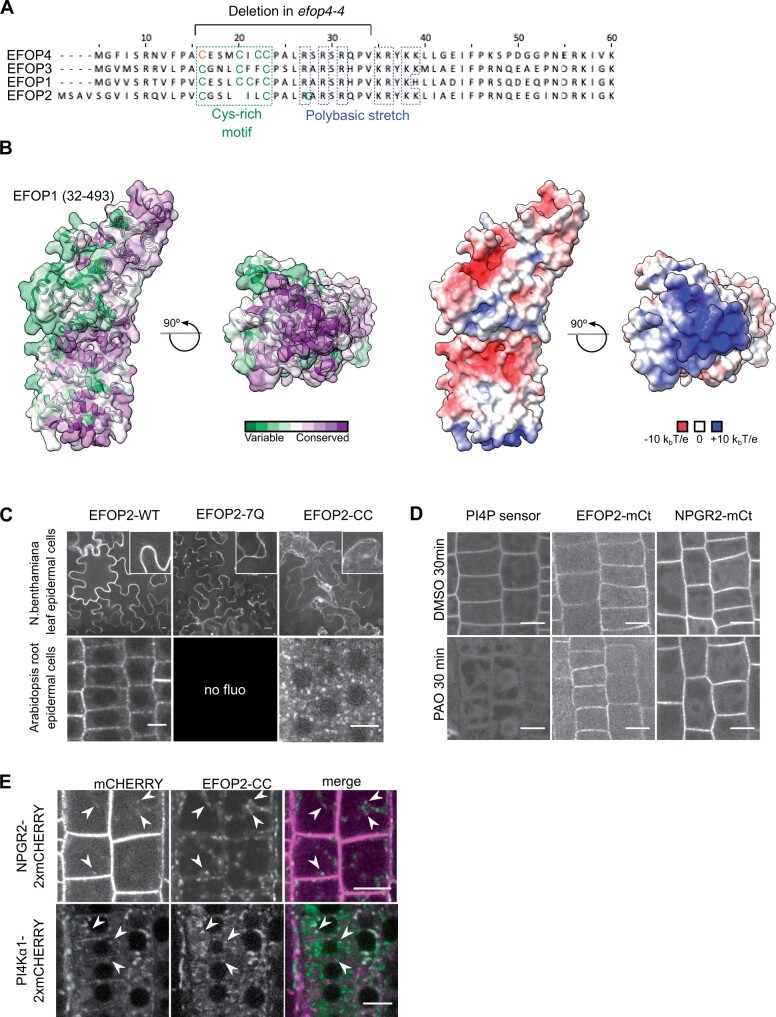
The EFOP subunit determines PI4Kα1 localization by lipid anchoring. A, N-terminal sequence alignment of EFOPs proteins. Conserved cys-rich motif (green) and polybasic patch (blue) are indicated, as well as, the deletion in *efop4-4* CrisPr allele. Bold cysteines are predicted as S-acetylated on the SwissPalm database with high (green) and medium (orange) confidence level. B, Template-based model of the EFOP1 structure (amino acid range 32–493). The left side shows an analysis of conserved amino acid residues mapped on the solvent-excluded surface of EFOP1. The right side shows electrostatic potential mapped on the solvent-excluded surface of EFOP1. The figure shows that the N-terminally located basic patch is highly conserved. C, Confocal images of EFOP2–mCITRINE (wild-type), EFOP2-7Q–mCITRINE, and EFOP2-CC–mCITRINE in *N. benthamiana* leaf epidermal cells and Arabidopsis root epidermal cells. Scale bar: 10 μm. D, Confocal images of PI4P sensor (PH domain of FAPP1 carrying the mutation E50A), EFOP2–mCITRINE (EFOP2-mCt), and NPGR2–mCITRINE (NPGR2–mCt) treated for 30 min with 30 μM PAO or the equivalent volume of DMSO. Scale bar: 10 μm. E, Confocal images of Arabidopsis root epidermal cells co-expressing *EFOP2-CC-mCITRINE* and *NPGR2-2xmCHERRY* or *PI4Kα1-2xmCHERRY*. White arrows indicate internal structures where the two signals colocalize. Scale bar: 10 μm.

We next decided to investigate the mechanism by which the PI4Kα1 complex is targeted at the plasma membrane. The four subunits of the PI4Kα1 complex are soluble proteins, without known lipid binding domains. The *efop3-1 efop4-4* and *efop3-2 efop4-4* mutants showed the same pollen lethality phenotype as the *efop3-1 efop4-2* and *efop3-2 efop4-2* mutant (Figure 4, A and B, Supplemental Figure 4, A and B). While *efop4-2* led to a very small-truncated protein (42 aa), the *efop4-4* allele led to a near full-length protein with only a small in frame deletion of 19 residues close to the EFOP4 N-terminus. This suggested that this N-terminal region is crucial for EFOP4 function (Figure 9A). Accordingly, the structural model of EFOP1 reveals that the N-terminal part of the protein forms a surface that is highly conserved among plants (Figure 9B). Moreover, this surface is enriched in positively charged amino acids (i.e. high electrostatic potential), suggesting that it could interact with the highly anionic plasma membrane.

The residues corresponding to the *efop4-4* deletion stand astride a nonstructured region (1–31 aa) and the positively charged surface visualized in the modeled EFOP structure (Figure 9, A and B). These residues are well conserved among the four EFOPs and include both a cysteine-rich motif, which could be S-acylated, and a polybasic region, which could contact anionic lipids at the plasma membrane (Figure 9A). We thus tested the potential role of those two elements in the regulation of EFOP localization and potentially the recruitment of the PI4Kα1 complex at the plasma membrane.

First, we evaluated the role of the polybasic patch in the N-terminus of EFOP proteins. Indeed, this region could be involved in targeting the entire PI4Kα1 complex to the plasma membrane through electrostatic interactions with anionic lipids, notably PI4P. In EFOP2, this region goes from aa 27 to aa 39 and contains seven positively charged residues (Figure 9A). We mutated all lysines/arginines into neutral glutamines and generated a *UBQ10_pro_:EFOP2-7Q–mCITRINE* construct. We observed that EFOP2-7Q–mCITRINE was soluble when transiently expressed in *Nicotiana benthamiana* leaf cells while the wild-type EFOP2–mCITRINE was localized to the plasma membrane, indicating that the polybasic patch in EFOP2 could be essential for plasma membrane targeting (Figure 9C). We next introduced the *UBQ10_pro_:EFOP2-7Q–mCITRINE* construct in Arabidopsis epidermal root cells. However, we did not retrieve any lines with a detectable fluorescent signal. It is likely that EFOP2-7Q–mCITRINE is unstable either because of misfolding or because EFOP2 needs to be associated with membrane to remain stable when expressed in Arabidopsis. Finally, we directly investigated the role of PI4P in the recruitment of the PI4Kα1 complex, by using phenylarsine oxide (PAO), a PI4K inhibitor (Balla et al., 2005; Simon et al., 2016). In this condition, the PI4P sensor is detached from the plasma membrane and relocalized in the cytosol (Figure 9D). However, neither NPGR2–mCITRINE nor EFOP2–mCITRINE was mislocalized upon PAO treatment. Thus, PI4P might not be involved in the targeting of the PI4Kα1 complex at the plasma membrane or the depletion of PI4P is not sufficient to delocalize the PI4Kα1 complex. In any case, this indicates that the presence of the PI4Kα1 complex at the plasma membrane relies, at least in part, on another mechanism.

We then investigated the role of the Cys-rich motif, which was deleted in the *efop4-4* allele. Such a motif could be a site of S-Acylation; a lipid posttranslational modification that can anchor protein to the plasma membrane (Zaballa and Goot, 2018). Indeed, according to the SwissPalm prediction software, this motif is predicted as S-acetylated with a high (in green) or medium level of confidence (in orange; Figure 9A). Confirming this hypothesis, all four Arabidopsis EFOP proteins were found to be S-acylated in a recent proteomic study (Kumar et al., 2020). Notably, all Cys-residues (boxed in Figure 9A) within the Cys-rich region of EFOP3 and EFOP4 were found to be S-acylated with high confidence in planta (Kumar et al., 2020). To experimentally test the importance of such lipid modification in EFOP localization, we mutated the two conserved cysteines (C20 and C23) into serine and generated a *UBQ10_pro_:EFOP2-CC-mCITRINE* construct. Similar to EFOP2-7Q–mCITRINE, we observed that EFOP2-CC–mCITRINE was soluble when transiently expressed in *N. benthamiana* leaf cells (Figure 9C). Next, we transformed the *UBQ10_pro_:EFOP2-CC-mCITRINE* construct into Arabidopsis and found that EFOP2-CC–mCITRINE was not localized at the plasma membrane of root meristematic cells and instead accumulated in intracellular structures (Figure 9C). All together, these data suggest that EFOP2 is likely targeted to the plant plasma membrane using lipid acylation anchoring.

### The EFOP/NPG/HYC complex targets PI4Kα1 to the plasma membrane

We then asked if EFOP proteins were sufficient to determine the localization of PI4Kα1 in the cell. Taking advantage of the EFOP2-CC construct localized in intracellular structures, we introgressed NPGR2–2xmCHERRY or PI4Kα1–2xmCHERRY in Arabidopsis expressing EFOP2-CC–mCITRINE and analyzed if EFOP2-CC was able to recruit NPGR2 or PI4Kα1 in those intracellular structures. We observed a weak signal of NPGR2 labeling intracellular compartments that partially colocalized with EFOP2-CC-containing structures. Similarly, PI4Kα1 was not only at the plasma membrane and soluble in the cytosol but also associated with the EFOP2-CC-containing structures (Figure 9E). This showed that EFOP is able to recruit NPGR2 and PI4Kα1 in different compartments of the cell.

**Figure 10:**
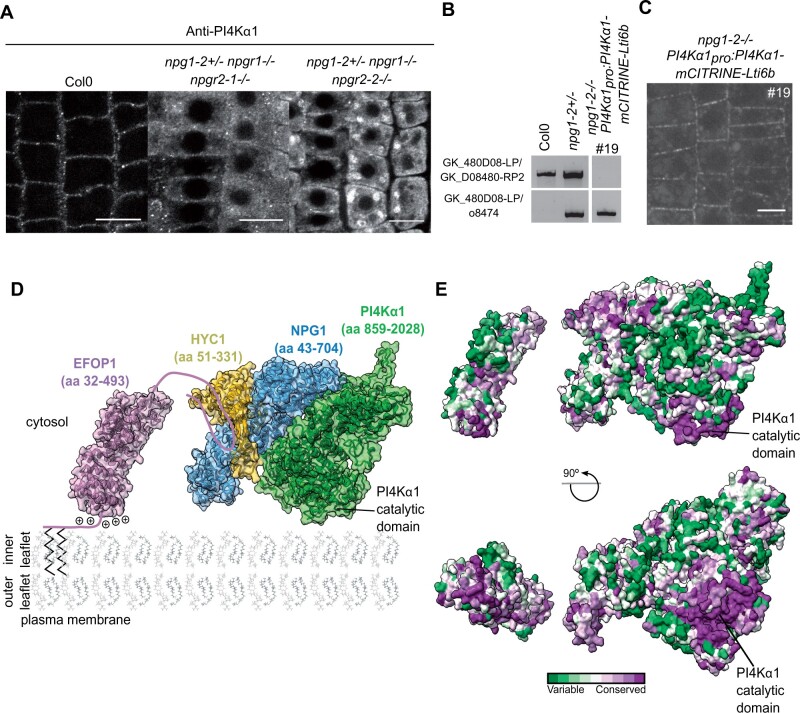
The NPG subunit acts as a scaffold around which HYC and PI4Kα1 structure themselves. A, Confocal images of PI4Kα1 using an anti-PI4Kα1 antibody on epidermal root cells of WT, *npg1-2^+/−^ npgr1^−/−^ npgr2-1^−/−^* and *npg1-2^+/−^ npgr1^−/−^ npgr2-2^−/−^* seedlings. Scale bar: 10 µm. B, Genotyping of Col0, *npg1-2* heterozygous plants and *npg1-2* homozygous plants complemented with *PI4Kα1_pro_:PI4Kα1-mCITRINE-Lti6b* (insertion no. 19). Upper shows amplification of the gene sequence. Lower shows amplification of the T-DNA border. C, Confocal images of *PI4Kα1_pro_:PI4Kα1-mCITRINE-Lti6b* in *npg1-2^−/−^* background (insertion no. 19). D, Schematic depiction of the plasma membrane targeting of the plant PI4Kα1 complex. The structure of the heterotrimeric complex composed of PI4Kα1 (green, amino acid region 859–2028), NPG1 (blue, amino acid region 43–704) and HYC1 (yellow, amino acid region 51–331), was obtained using template-based modeling and protein–protein docking. The EFOP1 N-terminal part (purple, amino acid region 32–493) was prepared by template-based modeling. Parts of EFOP1 with no homologous structure or predicted as intrinsically disordered are depicted as continuous lines. The S-acylated N-terminus of EFOP1 is shown together with plus signs depicting the polybasic patch of EFOP1. E, Analysis of conserved amino acid residues mapped on the solvent-excluded surface of the heterotrimeric complex (PI4Kα1–NPG1–HYC1) and EFOP1.

Because NPG proteins likely bridge PI4Kα1 to the membrane-binding EFOP subunits, we reasoned that they should contribute to the targeting of PI4Kα1 at the plasma membrane. To test this hypothesis, we monitored the localization of PI4Kα1 in the *NPG* loss-of-function background. We performed PI4Kα1 immunolocalization on the *npg1-2^+/−^ npgr1^−/−^ npgr2-1^−/−^* and *npg1-2^+/−^ npgr1^−/−^ npgr2-2^−/−^* triple mutants in which one functional allele on *NPG1* is still expressed. In the wild-type, we observed 38 roots over four independent experiments and found 33 roots that showed a clear plasma membrane labeling and 5 roots without any labeling. In contrast in the *npg* triple sesquimutants, we observed that PI4Kα1 was highly soluble and aggregated within the cytoplasm and showed only a faint labeling at the plasma membrane, likely due to the remaining expression of *NPG1* (Figure 10A). Such cytosolic/aggregated labeling was observed for 13 roots out of 17 for the *npg1-2^+/−^ npgr1^−/−^ npgr2-1^−/−^* allelic combination (observed over three independent experiments, three roots with no cytosolic aggregates and one root without any signal) and 26 roots out of 44 for the *npg1-2^+/−^ npgr1^−/−^ npgr2-2^−/−^* allelic combination (observed over four independent experiments, nine roots with no cytosolic aggregates and nine roots without any signal). This result indicates that PI4Kα1 plasma membrane targeting requires NPG proteins.

Using a different approach, we also generated a fusion between PI4Kα1–mCITRINE and the transmembrane protein Lti6b in order to artificially target PI4Kα1 at the plasma membrane and thus bypass the role of the NPG/HYC/EFOP complex. The *PI4Kα1_pro_:PI4Kα1-mC*ITRINE*-Lti6b* construct was able to rescue the *npg1-2* as we were able to recover homozygous *npg1-2* mutants upon expression of this particular transgene (Figure 10B). This indicates that *npg1* pollen lethality is likely due to the absence of PI4Kα1 at the plasma membrane during pollen development, thereby confirming (1) the functional link between NPG proteins and PI4Kα1 and (2) that the function of NPG proteins is to target PI4Kα1 to the plasma membrane. As discussed above, tagged versions of PI4Kα1, including PI4Kα1–mCITRINE, were not functional as they did not complement the *pi4kα1* mutant (Supplemental Table S3). Similarly, we did not retrieve any complemented *pi4kα1* mutant line expressing *PI4Kα1_pro_:PI4Kα1-mCITRINE-Lti6b* (Supplemental Table S3)*.* This raised the question on how the nonfunctional PI4Kα1–mCITRINE-Lti6b chimeric construct was able to rescue the *npg1-2* mutant. One possibility is that PI4Kα1 naturally forms homodimers. In that scenario, the nonfunctional PI4Kα1–mCITRINE–Lti6b would recruit the endogenous—and functional—PI4Kα1 at the plasma membrane in the absence of *npg1*, and thus complement the *npg1* mutant but not the *pi4kα1* mutant. We currently do not know whether PI4Kα1 is able to dimerize. However, structural data from the animal field showed that the PI4KαIII complex dimerizes at the plasma membrane (Lees et al., 2017), suggesting that it could also be the case in plants.

In these plants, PI4Kα1–mCITRINE–Lti6b is located in clusters at the plasma membrane while Lti6b is known as being rather homogenously localized at the plasma membrane (Figures 7, D and 10, C). The chimeric PI4Kα1–mCITRINE–Lti6b proteins might be restricted in clusters by other factors, which indicate that the subcompartmentalization of PI4Kα1 complex might not be an intrinsic property of the complex but rather come from interactions between PI4Kα1 and other lipids or proteins. Furthermore, it might indicate that the subcompartmentalization of PI4Kα1 complex is an essential feature for the proper function of the complex.

Altogether, this study shows that PI4Kα1 forms a heterotrimeric complex with NPG and HYC proteins. EFOP proteins target the PI4Kα1 complex to the plasma membrane by a combination of lipid anchoring and electrostatic interactions (Figure 10D). When aligned with the plasma membrane, the PI4Kα1 complex displays an evolutionarily conserved surface composed of the N-terminal basic patch of EFOP, the catalytic domain of PI4Kα1 and the N-terminal part of NPG. This highly conserved surface likely represents a membrane interacting interface of the heterotetrameric PI4Kα1 complex and provides initial mechanistic insight into the mode of function of PI4Kα1 in plants (Figure 10E).

## Discussion

### The plant PI4Kα1 complex is essential for cell survival

In this study, we showed that the loss-of-function of the PI4Kα1 leads to lethality of the male gametophyte. Similarly, knockouts of HYC1 and EFOP proteins mimic this phenotype, supporting the idea that these proteins act as a complex. Surprisingly, *npg1* single mutation is pollen lethal but does not result in the same morphological defects as observed in *pi4kα1* or *hyc1* mutants. The combination of loss of function of NPG1, NPGR1, and NPGR2 gives rise to deformed and shriveled pollen grains, indicating that during pollen development the three NPGs are expressed and partially redundant. NPG1 specifically could be needed for later steps of pollen development and germination, explaining the pollen lethality of NPG1 despite the absence of morphological defect. Indeed, pollen apertures are established in distinct membrane domains enriched in PI4P and PI(4,5)P_2_ (Lee et al., 2018). Like *npg1* mutant, loss-of-function of *SEC3A*, a gene encoding a subunit of the exocyst complex, is pollen lethal (Bloch et al., 2016; Li et al., 2017). *sec3a* mutant pollen grains do not present a morphological defect yet they do not germinate. SEC3a is recruited at the pollen germination sites and its binding to the plasma membrane depends on positively charged amino acids. Thus, NPG1 could participate in the recruitment of PI4Kα1 at aperture sites, which could be necessary to landmark the germination site, and the subsequent recruitment of SEC3a or other proteins. However, we showed that NPGR2 complements the *npg1* phenotype when expressed under the *NPG1* promoter, which suggests that the difference between NPG1 and NPGR2 function is mostly at the transcriptional level.

In addition to gametophytic phenotypes, we also observed that the loss of *HYC2* induces embryo lethality while *npg* triple sesquimutants and *PI4Kα1* knockdown display severe growth phenotypes at rosette and seedling stages. This is concordant with the idea that PI4Kα1 has a critical role for the cell function, not only during gametophytic but also sporophytic development. PI4P is crucial for the plasma membrane surface charge (Simon et al., 2016; Platre et al., 2018). Thus, it is likely that the loss of the PI4Kα1 affects the membrane surface charge and leads to the mislocalization of a whole set of proteins (Barbosa et al., 2016; Simon et al., 2016; Noack and Jaillais, 2020).

The Arabidopsis genome encodes two other PI4Kinases, PI4Kβ1 and PI4Kβ2, localized at the TGN/EE and at the cell plate (Preuss et al., 2006; Kang et al., 2011; Lin et al., 2019). As the plasma membrane and the TGN/EE are intimately linked through vesicular trafficking, the interdependence of the TGN/EE and the plasma membrane pool of PI4P remained an open question (Dubois and Jaillais, 2021). *pi4kβ1 pi4kβ2* double mutant and *pi4kα1* mutant display developmental phenotypes, which indicate that these lipid kinases are not fully redundant. This is coherent with the different subcellular localization of PI4Kβ1/PI4Kβ2 and PI4Kα1 in plant cells. However, only PI4Kα1 is essential in plants, while the *pi4kβ1 pi4kβ2* double mutant harbors mild phenotypes. This contrasts with yeast, in which the loss of either *pik1* or *sst4* is lethal (Audhya et al., 2000). It is thus possible that in plants, the lack of PI4Kβs at the TGN is compensated by PI4Kα1 activity, perhaps through the endocytosis of plasma membrane PI4P. In fact, a large portion of the *pi4kβ pi4k1β2* double mutant phenotype can be attributed to its function in cytokinesis (Lin et al., 2019). Thus, PI4Kα1 activity is not able to compensate for PI4Kβs function during cell division.

Using inducible PI4Kα1 knockdown, we confirmed a role for PI4Kα1 in the production of PI4P at the plasma membrane. However, using a knockdown strategy, we cannot determine the relative contribution of PI4Kα1 in the total PI4P production. Yet, we can speculate about this point. Indeed, we know that (1) the *pi4kβ1 pi4kβ2* double mutant, in which both kinases are fully knocked-out, has no detectable diminution of total PI4P (Lee et al., 2019) and (2) the pool of PI4P at the plasma membrane is quantitatively much more abundant than in the TGN (Simon et al., 2016; Dubois and Jaillais, 2021). Together, we can thus hypothesize that PI4Kα1 is likely responsible for most of the PI4P production in plant cells. The fact that PI4Kα1, but not PI4Kβs, is essential for cell survival argues in favor of this model, which nonetheless remains to be experimentally validated. Perhaps the use of inducible or tissue-specific knockout using CRISPR-cas9 technology will enable this point to be more directly addressed in the future.

### Cell wall defect in pollen grains

From meiosis to the end of microspore development, the tapetum establishes the exine layers around the pollen grain by secreting sporopollenin, while the intine layer is synthesized by the pollen (Borg and Twell, 2011). Many male sterile mutants have been identified in Arabidopsis and many of them are involved in pollen cell wall formation. This includes defects of secretion, biosynthesis or transport in the tapetal cells or microspores or callose and cellulose deposition by the microspores (Borg and Twell, 2011). Transmission electron microscopy of *pi4kα1*, *npg* triple sesquimutants, *hyc1* and *efop3 efop4* pollens reveal that they present a thicker an irregular intine layer. Thus, it is likely that the secretion of cell wall materials by the micropores is affected. The intine layer has a composition similar to a primary cell wall with cellulose, hemicellulose and pectin. Loss-of-function mutants for subunit of the cellulose synthase complex—*cesa1* and *cesa3*—are pollen sterile with shrivelled pollen grains similar to the *pi4kα1* complex mutants (Persson et al., 2007). In *cesa* mutants, a nonhomogeneous deposition of intine is observed, revealing the importance of cellulose microfibrils in guiding intine deposition. The TPLATE complex has also been involved in pollen cell wall deposition (Van Damme et al., 2006). *Tplate* mutants are male sterile with shriveled pollens due to abnormal deposition of callose in the intine layer. This may be explained by a poor regulation of callose synthase endocytosis in the *tplate* mutant. It is possible that callose synthase endocytosis mediated by the TPLATE complex and/or cellulose is also affected in *pi4kα1* complex mutants.

The *efop2 efop3* mutant also presents pollen morphological defects. However, it was possible to obtain a double homozygous plant, indicating that invalidating *efop2 efop3* does not lead to gametophytic lethality. Therefore, it is possible that the defect observed on the pollen grains does not come from the microspores but from an incorrect regulation of the secretion of material by the tapetum. Another possibility is that the first steps of pollen development before meiosis are impaired in *efop2 efop3* mutant. Interestingly, during microspore development, tapetal cells acquire a huge ER compartment that lays beneath the plasma membrane (Borg and Twell, 2011). Mutants with defects in genes involved in ER–Golgi transport such as *sec31b*, which encodes a protein that belongs to the COPII complex, present a similar phenotype to that of the *efop2 efop3* mutant with a combination of normal, deformed and collapsed pollen grains (Zhao et al., 2016). We can thus speculate that the PI4Kα1-driven production of PI4P at the plasma membrane might directly or indirectly impact the ER structure, dynamics and/or interaction with the plasma membrane. However, this hypothesis requires further investigation (Dubois and Jaillais, 2021).

### Function of the NPG–HYC–EFOP complex

Our study also showed that PI4Kα1’s plasma membrane localization is mediated by interactions with NPG and EFOP proteins rather than by a putative PH domain. At first, this PH domain was thought to localize PI4Kα1 at the plasma membrane through interaction with anionic phospholipids (Stevenson et al., 1998; Xue et al., 1999; Stevenson-Paulik et al., 2003). However, a region corresponding to the putative Arabidopsis PH domain correspond to the helical (cradle) and catalytic domains of human PI4KIIIα (Supplemental Figure 8; Lees et al., 2017; Dornan et al., 2018). Instaed, we found that PI4Kα1’s plasma membrane localization depends on the S-acylation of the EFOP proteins. Several S-acylated peptides in addition to the one in the N-terminal region have been found in EFOP proteins (Kumar et al., 2020). As S-acylation is a reversible posttranslational modification, differential S-acylation could be a mechanism explaining the different localization observed for EFOP1 and EFOP2 in the cell.

NPGs bridge PI4Kα1 and HYC with EFOP proteins. In addition, NPGs are calmodulin (Cam)-binding protein. Indeed, NPG1 can interact with several Cam isoforms in the presence of Ca^2+^ and has been suggested to play a role in Ca^2+^-dependent pollen tube germination (Golovkin and Reddy, 2003). Ca^2+^ is also intimately connected with phosphoinositides, membrane properties, and endomembrane trafficking (Himschoot et al., 2017). Ca^2+^ can directly bind anionic phospholipids modulating membrane electrostatics locally, preventing or promoting the recruitment of lipid-binding proteins, inducing the formation of PI(4,5)P_2_ clusters and facilitating membrane fusion (McLaughlin and Murray, 2005; Li et al., 2014). As phosphoinositides can bind Ca^2+^, diffuse in the membrane, and release Ca^2+^ somewhere else, they have been suggested to buffer and modulate Ca^2+^ signaling at the subcellular level. Ca^2+^ is also known to regulate many actors of endomembrane trafficking including regulators of the cytoskeleton, TPLATE complex, ANNEXINs, and SYNAPTOTAGMINs (Carroll et al., 1998; Van Damme et al., 2006; Schapire et al., 2008; Bürstenbinder et al., 2013, 2017; Hepler, 2016). For instance, the ER–plasma membrane-tethered protein SYT1 contains C2 domains that bind Ca^2+^ and phosphoinositides (Yamazaki et al., 2008; Giordano et al., 2013; Idevall-Hagren et al., 2015; Pérez-Sancho et al., 2015; Lee et al., 2019; Ruiz-Lopez et al., 2021). The PI4Kα1 complex could be localized at ER–plasma membrane contact sites and participate in Ca^2+^ signaling at this junction through calmodulin binding.

If EFOPs anchor the complex at the membrane and NPGs bridge PI4Kα1 and EFOPs, the role of HYCCIN-CONTAING proteins in the complex is less clear. In mammals, FAM126A, which has a HYCCIN domain, is involved in the stability of the complex and TTC7 folding (Dornan et al., 2018). In humans, FAM126A mutations lead to severe cases of hypomyelination and congenital cataract formation (Miyamoto et al., 2014; Baskin et al., 2016). However, even complete knockout of FAM126A is not lethal, while loss-of-function of PI4KIIIα is (Baskin et al., 2016). The FAM126A subunit is not present in yeast, suggesting that HYCCIN-domain containing proteins may have an accessory function in the PI4Kα1 complex, rather than an essential one. In contrast, we found that in Arabidopsis both *hyc1* and *hyc2* mutations are lethal as the corresponding wild-type genes are required for male gametophyte and embryo development, respectively. Our result thus suggests that HYC is an essential subunit for the function of the PI4Kα1 complex in plants. In humans, there are additional HYCCIN-containing proteins, including FAM126B. Our results thus open the possibility that FAM126A is not the only HYCCIN-containing protein contributing to PI4KIIIα function in metazoa.

### Formation and possible function of PI4Kα1-containing nanoclusters

The PI4Kα1 complex localizes at the plasma membrane in nanodomains. In yeast, Stt4 is found in large clusters called PIK patches (Baird et al., 2008). However, the clusters of Stt4 do not correspond with clusters of PI4P that probably diffuse laterally in the membrane. Similarly in Arabidopsis, PI4P biosensors are not clustered at the plasma membrane (Vermeer et al., 2009; Simon et al., 2014, 2016). This is in accordance with in vitro data showing that PI4P inhibits the catalytic activity of PI4Kα1 (Stevenson-Paulik et al., 2003). In addition, we showed that these nanodomains are immobile in plants, despite the fluidity of the plasma membrane (Jaillais and Ott, 2020). It is possible that unknown interactions with transmembrane protein, cytoskeleton or lipid nanodomains stabilize the PI4Kα1 complex.

Do these nanodomains correspond to a functional unit? Among many possibilities, they could correspond to ER–plasma membrane contact sites. Indeed, in yeast, Stt4 resides at these contacts (Omnus et al., 2018). Another hypothesis is that they could be a zone of attachment between the plasma membrane and actin cytoskeleton. PI4Kα1 has been purified from the F-actin-rich fraction from carrots (*Daucus carota*) and associates with polymerized F-actin in vitro (Stevenson et al., 1998). Additionally, in budding yeast, Stt4p is necessary for a proper actin cytoskeleton organization (Audhya et al., 2000; Foti et al., 2001). The two hypotheses are not mutually exclusive. Indeed, the actin disorganization phenotype of *stt4* mutant is rescued in yeast by the knockout of Sac1p, a PI4P phosphatase that resides in the ER membrane (Foti et al., 2001). Stt4p and Sac1p together control the PI4P gradient at membrane contact sites (Noack and Jaillais, 2020).

PI(4,5)P_2_ is enriched in nanodomains at the plasma membrane in pollen tubes (Kost et al., 1999; Fratini et al., 2021). These nanodomains contain PIP5K2 and are involved in actin dynamics. We speculate that the PI4Kα1 complex could also be targeted to these nanodomains in order to catalyze a phosphorylation cascade from PI to PI4P to PI(4,5)P_2_. In this model, PI4Kα1 inhibition by its substrate could help to coordinate the subsequent phosphorylation of PI4P by PIP5K2. In any case, given the absolute requirement on the PI4Kα1 complex for plant cell survival, deciphering the mechanisms behind the precise spatiotemporal regulation of this complex and the associated functions will be a fascinating challenge for the future.

## Methods

### Plant material

*Arabidopsis thaliana*, ecotype Columbia (Col-0) was used in this study, except for *pi4kα1-2*, which is in the Ws background. Plants expressing *2xp35S_pro_:myrimCITRINE-mCITRINE* were obtained from Jaillais et al., 2011. The PI4P sensor lines, *UBQ10_pro_::mCITRINE-1xPH^FAPP1^* and *UBQ10_pro_:mCITRINE-P4M^SidM^*, were obtained from (Simon et al., 2014, 2016; Supplemental Table S3).

### In vitro culture conditions

Seeds were surface-sterilized by the addition of 4 mL of HCl 37% in 100 mL of bleach for 4 h before plating on Murashige and Skoog (MS, Duchefa Biochemie) media supplemented with 0.8% plant agar and containing the appropriate antibiotic or herbicide. Glufosinate, kanamycin, hygromycin, and sulfadyacin were used at 10 mg·L^−1^, 50 mg·L^−1^, 30 mg·L^−1^, and 75 mg·L^−1^, respectively. Plates were placed under continuous white light conditions (LED 150 µmol·m^−2^·s^−1^) for 7–10 days. Resistant and sensitive seedlings were counted for segregations.

### Culture condition on soil

Seeds were directly sown in soil or 7-day-old seedlings were transferred from in vitro plates to soil. Plants were grown at 21°C under long day conditions (16-h light, LED 150 µmol·m^−2^·s^−1^).

### Sequence analysis

Sequence alignments were performed using Muscle WS software and edited with Jalview 2.0 software. Clustal color code was used. Domains were identified using the SMART software. Predicted lipid modification sites were found using the GPS-Lipid software.

### Structure modeling

To model a structure of individual PI4Kα1 complex subunits, we used several different threading algorithms, namely Swiss-model (Waterhouse et al., 2018), HHPred (Zimmermann et al., 2018), and RaptorX (Källberg et al., 2012). All three threading algorithms lead to highly similar results. For subsequent protein–protein docking and analyses, we used the models calculated using the Swiss-model program as the obtained models had the best ProSA score (Wiederstein and Sippl, 2007). Next, we utilized a hybrid protein–protein docking algorithm implemented in the HDOCK program (Yan et al., 2020) to position PI4Kα1 to NPG1 and NPG1 to HYC1. To analyze amino acid conservation, we employed the Consurf server with default parameters (Ashkenazy et al., 2016). The electrostatic potential of EFOP1 was calculated by solving the nonlinear Poisson–Boltzmann equation using the APBS-PDB2PQR software suite (Baker et al., 2001; Dolinsky et al., 2004). The structures were visualized in the ChimeraX program (Pettersen et al., 2021).

### Cloning of reporter lines

The following vectors were published before: Lti6b/pDONR207 (Elsayad et al., 2016), UBQ10prom/pDONR P4-P1r (Jaillais et al., 2011), 2X35S/pDONR P4-P1r (Marquès-Bueno et al., 2016), mCITRINE/pDONR 221 (Simon et al., 2014), mCITRINE/pDONR P2R-P3 (Jaillais et al., 2011), and 2xmCHERRY-4xmyc/pDONR P2R-P3 (Simon et al., 2014; Supplemental Table S4). For other entry vectors, coding gene or genomic gene sequences and 3′- untranslated region (UTR) sequences were cloned using Gateway technology (Invitrogen). Promoter sequences, Lti6b-FRB (pDONR221) and mCITRINE-3xSAG-Lti6b (pDONR P2R-P3) were cloned by Gibson Assembly method (Biolab). The coding sequences were amplified by PCR using the high fidelity Phusion Hot Start II (Thermo Fisher Scientific) taq polymerase and the indicated primers and template (Supplemental Table S5). The polymerase chain reaction (PCR) products were purified using the NucleoSpin Gel and PCR Clean kit (Macherey-Nagel) and recombined in the indicated vector using BP Clonase^TM^ II Enzyme Mix (Invitrogen) or Gibson Assembly mix (Biolab; Supplemental Table S5). Thermocompetent DH5α *Escherichia coli* were transformed with the corresponding vectors and plated on LB (Difco LB Broth, Lennox, #214010, 20 g·L^−1^, 15% agar, Difco Bacto Agar) containing kanamycin at 50 mg·L^−1^. Plasmids were purified using the Nucleospin Plasmid kit (Macherey-Nagel) and inserts sequenced. The expression vectors containing the promoter-gene-fluorescent tag cassette or 3′-UTR were obtained using the LR clonase-based three-fragment recombination system (Invitrogen), the pB7m34GW/pH7m34GW/pK7m34GW/pS7m43GW (Karimi et al., 2007) and pLOK180_pR7m34g (gift from L. Kalmbach) destination vectors, and the corresponding entry vectors (Supplemental Tables S3 and S4). Only EFOP2-7Q was introduced in the destination vector pK7FWG2 using the LR clonase-based one-fragment recombination system (Invitrogen; Karimi et al., 2002). Thermocompetent DH5α cells were transformed and selected on LB plates (Difco LB Broth, Lennox, #214010, 20 g·L^−1^, 15% agar, Difco Bacto Agar) containing 100 mg·L^−1^ spectinomycin. Plasmids were purified using the Nucleospin Plasmid kit (Macherey-Nagel) and inserts sequenced.

### Site-directed mutagenesis

Plasmids were amplified by PCR using the high-fidelity Phusion Hot Start II (Thermo Fisher Scientific) taq polymerase and the indicated overlapping primers carrying mutations (Supplemental Table S6**)**. PCR products were digested using Dpn1 and cutsmart buffer (Biolab). Thermocompetent DH5α *E. coli* were transformed with the digested PCR product and plated on LB (Difco LB Broth, Lennox, #214010, 20 g·L^−1^, 15% agar, Difco Bacto Agar) containing the appropriate antibiotic. Plasmids were purified using the Nucleospin Plasmid kit (Macherey-Nagel) and mutations were sequenced.

### Artificial microRNA designed

Artificial microRNAs were designed using Web MicroRNA Designer (http://wmd3.weigelworld.org/cgi-bin/webapp.cgi). DNA synthesis containing AttB1-AttB2 sites and the miR319a modified with the sequence of the artificial microRNA (Supplemental Table S9) were ordered at Integrate DNA TechnologyR. The DNA fragments were recombined in the pDONR 221 (InvitrogenR) using BP ClonaseTM II Enzyme Mix (InvitrogenR).

### Agrobacterium transformation

Electrocompetent *Agrobacterium tumefaciens* (C58pmp90) were transformed by electroporation. One microliter of DNA plasmid at a concentration of 0.25–1 μg·μL^−1^ was added into 50 µL of electrocompetent agrobacterium on ice. The agrobacteria were transferred into a cold 1mm wide electroporation chamber (Eurogentec, #CE00150). A pulse of 2 kV, 335 Ω, 15 µF, for 5 ms was performed on the electroporation chamber using the MicropulserTM (Bio-Rad, #165-2100). One microliter of liquid LB media was added and the bacteria were placed into a new tube and incubated at 29°C for 2–3 h. The agrobacteria were selected on LB (Difco LB Broth, Lennox, #214010, 20 g·L^−1^, 15% agar, Difco Bacto Agar), or YEB (0.5% beef extract, 0.1% yeast extract, 0.5% peptone, 0.5% sucrose, 1.5% bactoagar, pH 7.2) plates containing the appropriate antibiotics to select the agrobacterium strain (50 mg·L^−1^ of rifampicin and 20 mg·L^−1^ of gentamycin) and the target construct (250 mg·L^−1^ of spectinomycin). Plates were incubated at 29°C for 48 h.

### Plant transformation and selection

Agrobacteria were spun and resuspended in 5% sucrose and 0.02% Silwet L-77 detergent. Arabidopsis were transformed by floral dipping.

Transformed seedlings were selected on MS (Duchefa Biochemie) media supplemented with 0.8% plant agar containing the appropriate antibiotic or herbicide. Basta, kanamycin, hygromycin, and sulfadiazin have been used at 10 mg·L^−1^, 50 mg·L^−1^, 30 mg·L^−1^, and 75 mg·L^−1^, respectively. Selection of the pLOK180_pR7m34g plasmid was done using the FastRed method (i.e. red fluorescent seeds).

### Protein extraction, IP, antibodies, and immunoblot analysis

Leaf tissue from wild-type plants and/or transgenic lines was collected, frozen in liquid nitrogen and ground to powder. The powder was resuspended in −20°C MetOH; 1% Protease inhibitor (P9599, Sigma-Aldrich) and incubated at −20°C for 5 min. Proteins were pelleted, resuspended in −20°C acetone and incubated at −20°C for 5min. Proteins were pelleted. The pellet was dried and resuspended in Protein Extraction Reagent (C0856, Sigma-Aldrich) supplemented with 1% protease inhibitor (P9599, Sigma-Aldrich). Protein extraction was then kept at −20°C.

Protein samples in 1 × SDG (Tris–HCL 0.25 M, 10% glycerol, 2% DTT, 2.5% sodium dodecyl sulphate [SDS]) supplemented with bromophenol blue were denaturated at 95°C for 5 min. Samples migrated on 7.5% (for proteins over 200 kDa) or 10% SDS–polyacrylamide gel electrophoresis polyacrylamide gel at 140 V with 1×Tris-Glycine SDS running buffer (Euromedex). Proteins were transferred to a nitrocellulose membrane (0.45 µm) in 1×Tris-Glycine (Euromedex), 20%EtOH transfer buffer at 100 V for 1 h. Membrane was blocked with 5% milk, 1X phosphate buffered saline-T (Dominique Dutscher), 2% Tween-20 for 1 h. Primary antibodies were used at 1/1,000 (anti-mCHERRY, anti-PI4Kα1) or at 1/2,000 (anti-GFP, anti-NPGR2; Supplemental Table S1) in 5% milk, 1X TBS, 0.1% Tween-20 overnight. The following antibodies are commercially available: anti-GFP (ThermoFischer, catalog # A6455) and anti-mCHERRY (Abcam, catalog # ab167453), while anti-PI4Kα1 and anti-NPGR2 were custom made by Proteogenix. For the anti-PI4Kα1, Proteogenix cloned, expressed in *E. coli* and purified a PI4Kα1 fragment corresponding to AA1-344, fused with 6xHis at its N-terminus and the antibodies were produced in Rat. For the anti-NPGR2 antibody, Proteogenix cloned, expressed in *E. coli* and purified a NPGR2 fragment corresponding to AA1-269, fused with 6xHis at its N-terminus, and the antibodies were produced in Rabbit. Secondary antibodies were used at 1/5,000 (anti-Rat IgG, HRP conjugate Merck, catalog # AP136P and anti-Rabbit IgG, HRP conjugate, Promega, catalog #W4011) in 1X TBS, 0.1% Tween-20 for 1 h. Protein revelation was done using the Clarity or Clarity Max ECL Western Blotting Substrates (Biorad) and the Chemidoc MP imaging system (Biorad).

For co-IP, leaf tissue from transgenic lines was collected, frozen in liquid nitrogen, and ground to powder. Proteins fused to mCITRINE were immunoprecipitated using the pull-down kit Miltenyi µMacs anti-GFP (Miltenyi Biotec). Crude extracts were put in 1XSDG supplemented with bromophenol blue and denatured at 95°C for 5 min, while IP samples were directly ready for migration on polyacrylamide gels.

Each co-IP experiment was repeated at least three times independently with similar outcomes.

### Mass spectrometry analysis

Mass spectrometry analysis was performed by the Protein Science Facility of SFR (Structure Fédérative de Recherche) Bioscience Lyon. Trypsine-digested samples were analyzed by liquid chromatography-tandem mass spectrometry (Orbitrap, ThermoFischer Scientific). The peptides identified were compared to those in the UniProtKb database.

### Yeast-two-hybrid

The initial screen was performed by hybrigenics services (https://www.hybrigenics-services.com/contents/our-services/interaction-discovery/ultimate-y2h-2), using the ULTImate yeast two hybrid (Y2H) screen against their Universal Arabidopsis Normalized library obtained using oligo_dT. The residues 2–1479 of PI4Kα1 were used. The screen was performed on 0.5-mM 3AT, 58.6 million interactions were analyzed, and 313 positive clones were sequenced.

AD vectors (prey) and DB vectors (bait) were transformed in the Y8800 and Y8930 yeast strains, respectively. Transformed yeasts were plated on a minimal synthetic defined (SD) medium (0.67% yeast nitrogen base, 0.5% dextrose anhydrous, 0.01% adenine, 1.8% agar) with all amino acids except tryptophan (SD-T) for AD vectors and SD-L for DB vectors for selection.

Yeast colony transformed with AD or DB vectors were grown in SD-T and SD-L liquid media, 30°C, 200 rpm for 48 h. Mating was performed at 30°C, 200 rpm, overnight using 10 µL of AD and 10-µL DB clones in 180-µL YEPD (1% yeast extract, 2% peptone, 2% dextrose anhydrous, 0.01% adenine) liquid media. Diploid yeasts were selected by addition of 100 µL of SD-LT liquid media. Yeast was grown for 48 h at 30°C, 200 rpm. Diploid yeasts were plated on different selective media: SD-LT to verify the mating; SD-LTH to select the positive interactions; SD-LTH + 3AT (3-Amino-1,2,4-Triazol at 1 mM final) to select strong interactions only: SD-LH+CHX (cycloheximide at 10 µg·mL^−1^ final) to determine the autoactivated DB clones; SD-LH+CHX + 3AT to determine which concentration of 3AT erased the autoactivation of DB clones. The experiment was repeated three times independently and similar results were obtained.

### Crispr lines

A 20-bp target sequence upstream of a 5′-NGG-3′ sequence in an exon of the gene of interest was found using the CrisPr RGEN tool (www.rgenome.net/). Primers were designed following the methods developed in Xing et al. (2014) and Wang et al. (2015; Supplemental Table S7**)**.

### T-DNA and Crispr mutant genotyping

Leaf tissue from wild-type plants, T-DNA insertion lines and Crispr lines were collected, frozen in liquid nitrogen and ground to powder. The powder was resuspended in the extraction buffer (200 mM of Tris, pH 7.5, 250 mM of NaCl, 25 mM of EDTA, 0.5% of SDS). DNA was precipitated with isopropanol and the DNA pellet was washed with 75% ethanol before resuspension in water.

Plants were genotyped by PCR using the GoTaq polymerase (Promega) and the indicated primers (Supplemental Table S8). PCR products were migrated on a 1% agarose gel or the percentage indicated (Supplemental Table S8). When sequencing was required, the bands were purified using the NucleoSpin Gel and PCR Clean kit (Macherey-Nagel) and sequenced (Sanger sequencing service, Eurofins) using the same primers as for the PCR.

### Pollen observation by SEM

Pollen grains from mutant or WT flowers were placed on tape and observed using the mini SEM Hirox 3000 at −10°C, 10 kV.

### Pollen inclusion and observation by transmission electron microscopy

For transmission electron microscopy, anthers were placed in a fixative solution of 3.7% paraformaldehyde and 2.5% glutaraldehyde, Na_2_HPO_4_ 0.1 M, NaH_2_PO_4_ 0.1 M overnight and postfixed in 1% OsO_4_, Na2HPO4 0.1 M, NaH_2_PO_4_ 0.1 M. Anthers were dehydrated through a graded ethanol series from 30% to 100% and embedded in SPURR resin. Sections were made using an ultramicrotome Leica UC7 at 70–80 nm and poststained with acetate uranyle 5% (in ethanol), lead citrate (in NaOH). Pollen was observed using a transmission electron microscope Jeol 1400 Flash. For the genotypes *pi4kα1-1, npg1-2^+/−^ npgr1^−/−^ npgr2-2^−/−^*, *hyc1*, and *efop3-1^−/−^ efop4-2^−/−^*, shriveled pollen grains were selected for imaging and compared with the wild-type pollen.

### Pollen staining

To perform Alexander staining, flowers that were about to open were dissected and anthers were put between slide and coverslip in Alexander staining solution (25% glycerol, 10% EtOH, 4% acetic acid, 0.05% acid fuchsin, 0.01% Malachite green, 0.005% phenol, 0.005% chloral hydrate, 0.005% Orange G) for 7 h at 50°C before observation under a stereomicroscope.

For DAPI staining, flowers that were about to open were dissected and their anthers were put between slide and coverslip in DAPI solution (10% DMSO, 0.1% NP-40, 50-mM PIPES, 5-mM EGTA, 0.01% DAPI) for 5 min at RT before observation with the confocal microscope. DAPI was excited with a 405 nm laser (80 mW) and fluorescence emission was filtered by a 447/60 nm BrightLine single-band bandpass filter (Semrock, http://www.semrock.com/).

### Seed clearing

Siliques were opened. The replums with the seeds attached were placed between slide and coverslip in clearing solution (87.5% chloral hydrate, 12.5% glycerol) for 1 h before observation at the microscope with differential interference contrast.

### Artificial microRNA phenotype

Surface-sterilized seeds were plated on MS (Duchefa BiochemieR) media supplemented with sucrose (10 g·L^−1^) containing 5-μM β-estradiol or the equivalent volume of DMSO. Plates were vernalized 3 days before being placed under continuous white light conditions for 11 days. Plates were scanned. Root length was measured using ImageJ software. Similar results were obtained for five independent experiments.

### Confocal imaging setup

The 7- to 10-day-old seedlings were observed with a Zeiss microscope (AxioObserver Z1, Carl Zeiss Group, http://www.zeiss.com/) with a spinning disk module (CSU-W1-T3, Yokogawa, www.yokogawa.com) and a Prime 95B camera (Photometrics, https://www.photometrics.com/) using a 63x Plan-Apochromat objective (numerical aperture 1.4, oil immersion) and the appropriate laser and bandpath filter.

GFP was excited with a 488 nm laser (150 mW) and fluorescence emission was filtered by a 525/50 nm BrightLine single-band bandpass filter (Semrock, http://www.semrock.com/). mCITRINE was excited with a 515 nm laser (60 mW) and fluorescence emission was filtered by a 578/105 nm BrightLine single-band bandpass filter (Semrock, http://www.semrock.com/). mCHERRY was excited with a 561 nm laser (80 mW) and fluorescence emission was filtered by a 609/54 nm BrightLine single-band bandpass filter (Semrock, http://www.semrock.com/). In the case of seedling expressing mCHERRY and mCITRINE markers, mCITRINE was excited and emission was filtered using the GFP settings.

For each line, several independent transformation events were used and observed in three independent experiments at minima.

TIRF microscopy used an objective based azimuthal ilas2 TIRF microscope (Roper Scientific) with 100× Apo NA 1.46 Oil objective. The exposure time used was 1s. HYC2-mCITRINE and NPGR2-mCITRINE were excited and emission was filtered using the GFP settings, while EFOP2-mCITRINE was excited and emission was filtered using the YFP settings.

### Immunolocalization

Six-day-old seedlings were fixed in PFA 4%, MTBS (50-mM PIPES, 5-mM EGTA, 5-mM MgSO_4_, pH 7) for 2 h. Roots were placed on Superfrost Plus slides (Thermo Scientific, #10149870), dried at RT for 1 h, and rehydrated using MTBS + 0.1% tritonx100. Permeabilization was done using enzymatic digestion (250 µL of 0.75% pectinase, 0.25% pectolyase, 0.5% macérozyme, 0.5% cellulase stock solution was diluted into 1-mL MTBS) for 40 min (Rozier et al., 2014). Roots were treated with 10% dimethylsulfoxide, 3% Igepal (Sigma, #CA-630) in MTSB for 1 h. Blocking was done using 5% normal goat serum (NGS Sigma #G9023) in MTSB. Roots were incubated with the primary antibody diluted at 1/100 (anti-PI4Kα1) and with secondary antibody diluted at 1/300 (anti Rat IgG, alexa Fluor 488 conjugate, InVitrogen-Molecular Probes, #A-21210) overnight at 4°C. Roots were placed between slide and coverslip in Vectashield (Vectorlabs, #H-1000-10) and observed using a confocal microscope Zeiss LSM800. Similar results were obtained in six independent experiments.

### Biochemical quantification of PIP levels in inducible PI4K knockdown.

Nine-day-old seedlings grown on 1/2 MS plates with 1% (w/v) sucrose, 1% (w/v) agar, and 5-μM β-estradiol or DMSO (control) were transferred to 2-mL Safe-lock Eppendorf tubes containing 190-µL labelling buffer (2.5-mM MES (2-[N-Morpholino]ethane sulfonic acid; pH 5.7 with KOH, 1-mM KCl) supplemented with either 5-μM β-estradiol or DMSO (control), with each tube containing three seedlings. Seedlings were metabolically labeled with radioactive phosphate by incubating them overnight for ∼16–20 h with 10 µL (5–10 µCi) carrier-free ^32^P-${\text{PO}}_{4}^{3-}$ (^32^P_i_; PerkinElmer, The Netherlands) in labeling buffer. Incubations were stopped by adding 50 µL of 50% (w/v) perchloric acid and the lipids extracted (Zarza et al., 2020). PIP was separated from the rest of the phospholipids by thin-layer chromatography (TLC) using K-oxalate-impregnated and heat-activated silica gel 60 plates (20 × 20 cm; Merck) and an alkaline TLC solvent, containing chloroform/methanol/25% ammonia/water (90:70:4:16; Munnik and Zarza, 2013). Each lane contained 1/5th of the extract. Radioactivity was visualized by autoradiography and quantified by phosphoimaging (Typhoon FLA 7000, GE Healthcare). PIP and PA levels were quantified as a percentage of total ^32^P-labeled phospholipids. Experiments were performed in quadruplicate.

### Microsomes and plasma membrane purification

Microsomes were purified as described in (Simon-Plas et al., 2002) and resuspended in phosphate buffer (5 mM, pH 7.8) supplemented with sucrose (300 mM) and KCl (3 mM). Plasma membrane was obtained after cell fractionation by partitioning twice in a buffered polymer two-phase system with polyethylene glycol 3350/dextran T-500 (6.6% each).

For PI4Kα1, proteins were precipitated using five volumes of −20°C acetone for one volume of protein extraction and incubated for 10 min at −20°C. Proteins were pelleted. This process was repeated two more times. Pellet was dried and resuspended in 1×SDG supplemented with bromophenol blue. All steps were performed at 4°C.

### Plasmolysis

The 7 to 10-day-old seedlings were placed between side and coverslip in MES 10 mM at pH 5.8 with or without 0.75-M sorbitol and observed using a confocal microscope.

### FRAP

Fluorescence in a rectangle region of interest (ROI) (5× 1.7 μm) at the plasma membrane was bleached in the root by successive scans at full laser power (150 W) using the iLas2 FRAP module (Roper scientific, http://www.biovis.com/ilas.htm). Fluorescence recovery was measured in the ROIs and in controlled ROIs (rectangle with the same dimension in unbleached area). FRAP was recorded continuously during 120 s with a delay of 1s between frames. Fluorescence intensity data were normalized as previously described (Martinière et al., 2012). The mobile fraction was calculated at *t* = 120s with the following formula: *I*(*t*)−Min(*I*)/Ictrl(*t*)/Min(*I*), where *I*(*t*) and Ictrl(*t*) are the intensity of the bleached and control region at time *t*, respectively. Data were obtained in several independent experiments.

### Nicotiana benthamiana leaf infiltration

Transformed agrobacterium were directly taken from plate with a tip and resuspended into 2 mL of infiltration media (10-mM MES, pH 5.7, 10-mM MgCl_2_, 0.15-mM acetosyringone [Sigma-Aldrich, #D134406]) by pipetting. The Optical Density at 600 nm (OD_600_) was measured using a spectrophotometer (Biophotometer, Eppendorf) and adjusted to 1 by adding infiltration media.

The infiltration was performed on the heart-shaped tobacco leaves of 2- to 3-week-old plants. Using a 1-mL syringe (Terumo, #125162229), the infiltration solution with the agrobacterium was pressed onto the abaxial side of the chosen tobacco leaf. The plants were returned to the growth chamber for 2–3 days under long day conditions.

The 5 mm^2^ regions of the leaf surrounding the place where the infiltration had been made were cut out. The pieces of leaf were mounted in water between slide and coverslip with the abaxial side of the leaf facing the coverslip. Using the appropriate wavelength, an epifluorescent microscope and the smallest objective (10×), the surface of the leaf was screened to find the transformed cells. Then, the subcellular localization of the fluorescent protein was observed using a spinning confocal microscope and 63× objective.

### PAO treatment

Seedlings (7- to 10-day old) were incubated in liquid MS with 30-µM PAO (Sigma, www.sigmaaldrich.com, PAO stock solution at 60 mM in DMSO) for 30 min before observation using a confocal microscope.

### Statistical analysis

Statistical analyses were performed using R (v. 3.6.1, (R Core Team, 2019)) and the R studio interface (Supplemental Table S10).

### Accession numbers

PI4Kα1, At1g46340; NPG1, At2g43040; NPGR1, At1g27460; NPGR2, At4g28600; HYC1, At5g21050; HYC2, At5g64090; EFOP1, At5g21080; EFOP2, At2g41830; EFOP3, At1g05960; EFOP4, At5g26850

## Supplemental data

The following materials are available in the online version of this article.

**Supplemental Figure S1.** Immunoblot validation of the custom-made anti-PI4Kα1 and anti-NPGR2 antibodies.

**Supplemental Figure S2.** Template-based modeling of PI4Kα1, NPG1, and HYC1.

**Supplemental Figure S3.** Characterization of *pi4kα1* pollen phenotype.

**Supplemental Figure S4.** Characterization of the pollen phenotype of single and multiple *npg*, *hyc*, and *efop* mutants.

**Supplemental Figure S5.** Sporophytic phenotypes and complementation of *npg* multiple mutants and *hyc2* single mutant.

**Supplemental Figure S6.** PI4Kα1, NPG, HYC, and EFOP protein localization in pollen grains.

**Supplemental Figure S7.** FRAP analysis of NPGR2/EFOP2/HYC2 fused with mCITRINE in Arabidopsis root.

**Supplemental Figure S8.** Position of the putative PH domain of PI4Kα1.

**Supplemental Table S1.** Antibodies used in this study.

**Supplemental Table S2.** Description of the single and multiple mutants analyzed in this study.

**Supplemental Table S3.** Transgenic lines.

**Supplemental Table S4.** Published vectors used in this study.

**Supplemental Table S5.** Primers used for cloning into gateway entry vectors.

**Supplemental Table S6.** Primers used for site directed mutagenesis.

**Supplemental Table S7.** Primers used for Crispr constructs.

**Supplemental Table S8.** Genotyping primers.

**Supplemental Table S9.** microRNA sequences.

**Supplemental Table S10.** Statistical analysis.

## Supplementary Material

koab135_Supplementary_Data

## Notes

L.C.N. was responsible of all the experiments described in this manuscript with the following exception: V.B. performed transmission electron microscopy experiments and helped with microscopy image acquisition and analyses; F.R. performed and imaged whole mount immunolocalization; A.M.-C. and S.M. purified plasma membranes; M.-C.C. initiated the yeast-two hybrid screen; L.A. helped with yeast-two hybrid experiments; R.P. performed structural modelling, F.D.S. and T.M. measured ^32^P PIP levels. L.C.N. prepared the figures, L.C.N. and Y.J. wrote the manuscript and all authors commented on the manuscript.

The author responsible for distribution of materials integral to the findings presented in this article in accordance with the policy described in the Instructions for Authors (https://academic.oup.com/plcell) is: Yvon Jaillais (yvon.jaillais@ens-lyon.fr).
