# Supplementary material for: A nanodomain-anchored scaffolding complex is required for the function and localization of phosphatidylinositol 4-kinase alpha in plants
Source: Plant Cell. 2021 May 19;34(1):302–32. doi: 10.1093/plcell/koab135 (PMC8774046; doi:10.1093/plcell/koab135)
Supplement: koab135_Supplementary_Data [file koab135_supplementary_data.zip › tpc.01032.2020-s01.pdf]

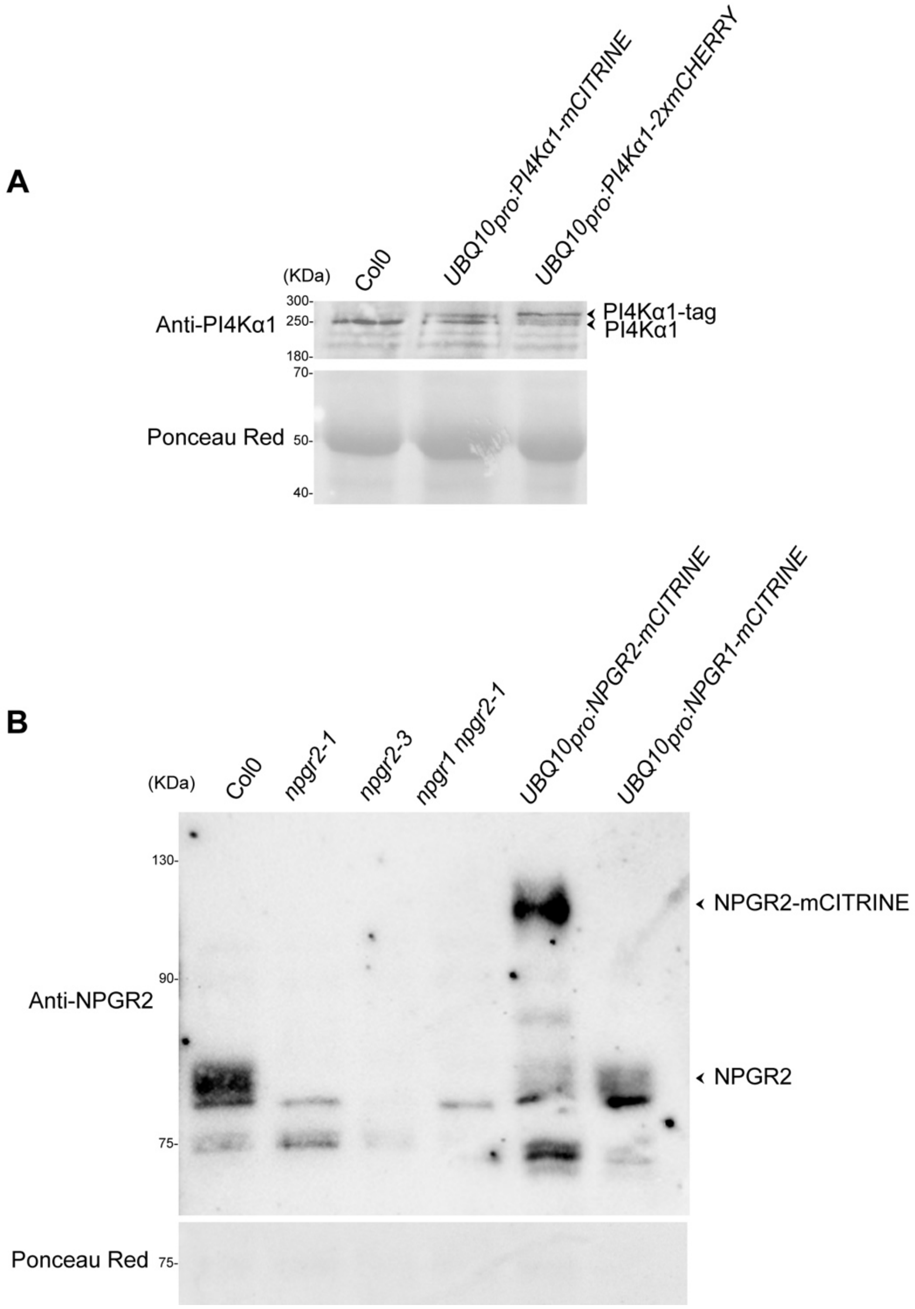

Supplemental Figure S1. Immunoblot validation of the custom-made anti-PI4Kα1 and anti-NPGR2 antibodies. (A) Immunoblot using anti-PI4Kα1 antibodies (Rat) on total protein extracts of Col-0 seedlings, seedling expressing *UBQ10$_{pro}$:PI4Kα1-mCITRINE* and seedling expressing *UBQ10$_{pro}$:PI4Kα1-2xmCHERRY*. Red ponceau shows similar protein loading in every well. **(B)** Western blot using anti-NPGR2 antibodies (Rabbit) on total protein extracts of Col0*, npgr2-1, npgr2-3, npgr1 npgr2-1* seedlings, seedling expressing *UBQ10$_{pro}$:NPGR2-mCITRINE* and seedling expressing *UBQ10$_{pro}$:NPGR1-mCITRINE*. Ponceau red shows similar protein loading in every well. Supports Figure 1.

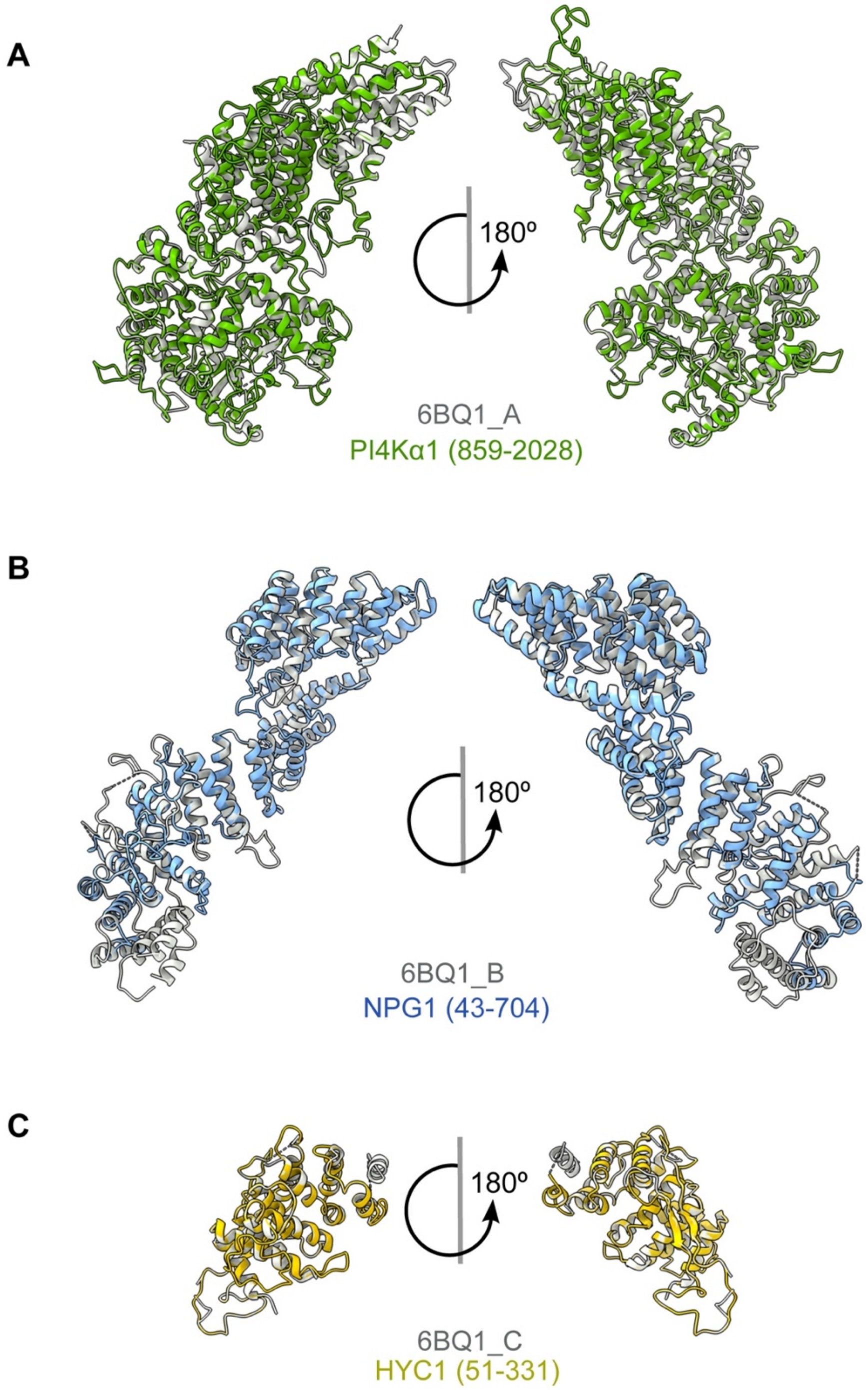

Supplemental Figure S2. Template-based modelling of PI4Kα1, NPG1 and HYC1.
(A) Superimposition of the template-based structure of Arabidopsis PI4Kα1 (green, amino acid region 859-2028) with the template structure of human PI4KIIIα (PDB code 6BQ1, chain A, in grey). **(B)** Superimposition of the template-based structure of Arabidopsis NPG1 (blue, amino acid region 43-704) with the template structure of human TTC7 (PDB code 6BQ1, chain B, in grey). **(C)** Superimposition of the template-based structure of Arabidopsis HYC1 (yellow, amino acid region 51-331) with the template structure of human FAM126A (PDB code 6BQ1, chain C, in grey). Supports Figure 2.

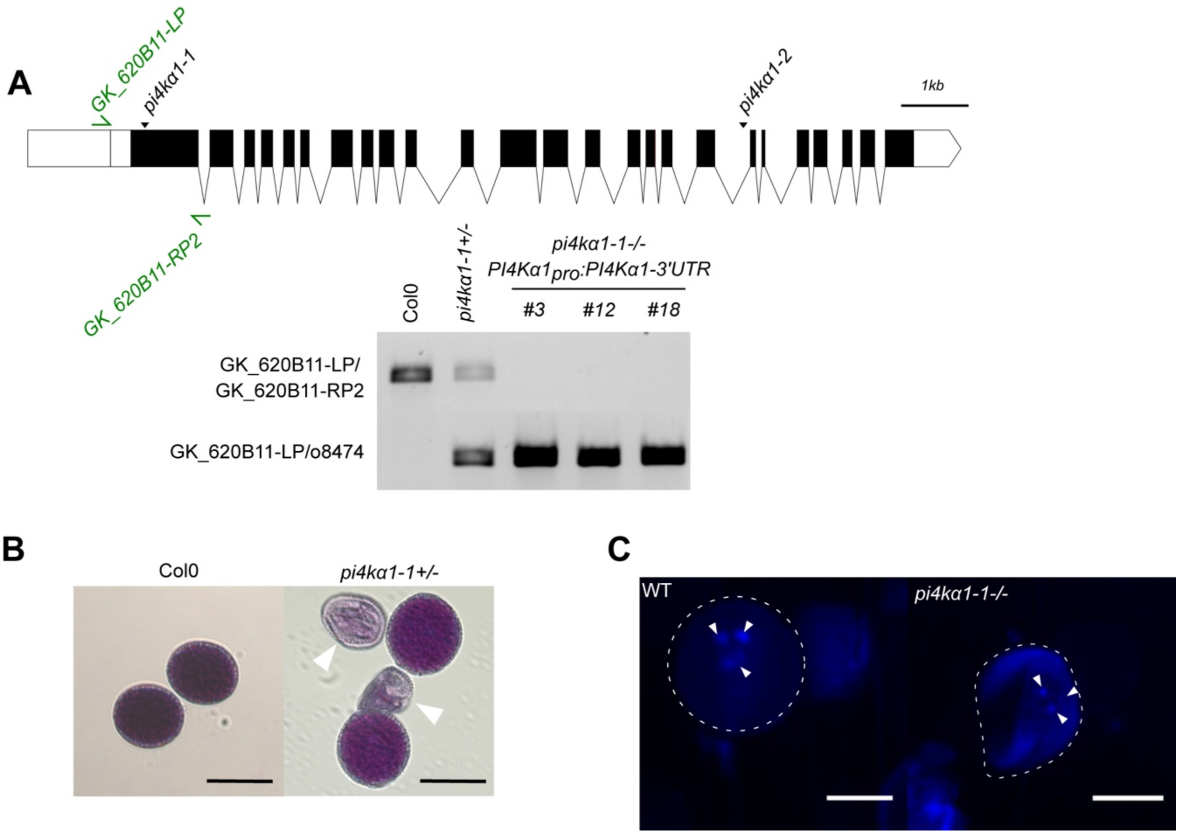

Supplemental Figure S3. Characterization of *pi4kα1* pollen phenotype. (A) Genotyping of Col-0, *pi4kα1-1* heterozygous plants, and *pi4kα1-1* homozygous plants expressing *PI4Kα1$_{pro}$:PI4Kα1-3'UTR* (insertion n°3, 12 and 18). Upper panel shows the amplification of gene sequence. Lower panel shows amplification of T-DNA border. **(B)** Alexander staining of pollen grains from Col-0 and self-fertilized *pi4kα1-1* heterozygous plants. Shrivelled pollen grains are indicated by white arrowheads. Scale bars: 20 µm. **(C)** DAPI staining of pollen grains from self-fertilized *pi4kα1-1* heterozygous plants with normal (WT, left) and shrivelled (*pi4kα1-1-/-,* right) shape. Nuclei are indicated with white arrowheads, dashed lines indicated the pollen grain contour. Scale bars: 10 µm. Supports Figure 3.

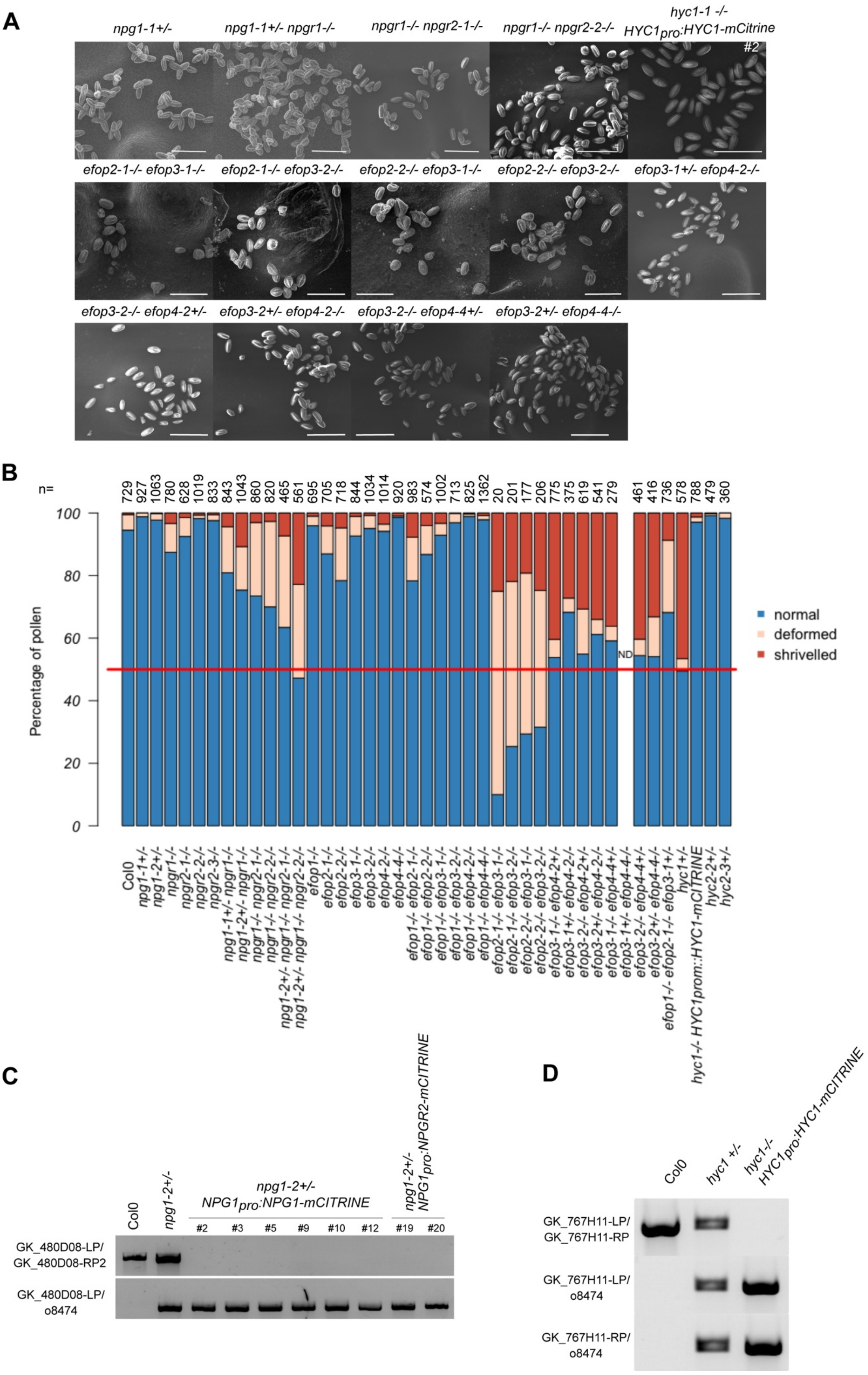
A
npg1-1+/-
npg1-1+/- npgr1-/-
npgr1-/- npgr2-1-/-
npgr1-/- npgr2-2-/-
hyc1-1 -/- HYC1pro:HYC1-mCitrine
#2
efop2-1-/- efop3-1-/-
efop2-1-/- efop3-2-/-
efop2-2-/- efop3-1-/-
efop2-2-/- efop3-2-/-
efop3-1+/- efop4-2-/-
efop3-2-/- efop4-2+/-
efop3-2+/- efop4-2-/-
efop3-2-/- efop4-4+/-
efop3-2+/- efop4-4-/-
B
n=
729 927 1063 780 628 1019 833 843 1043 860 820 465 561 695 705 718 844 1034 1014 920 983 574 1002 713 825 1362 20 201 177 206 775 375 619 541 279 461 416 736 578 788 479 360
Percentage of pollen
100 80 60 40 20 0
ND
normal
deformed
shrivelled
Col0
npg1-1+/-
npg1-2+/-
npgr1-/-
npgr2-1-/-
npgr2-2-/-
npgr2-3-/-
npg1-1+/- npgr1-/-
npg1-2+/- npgr1-/-
npgr1-/- npgr2-1-/-
npgr1-/- npgr2-2-/-
npg1-2+/- npgr1-/- npgr2-1-/-
npg1-2+/- npgr1-/- npgr2-2-/-
efop1-/-
efop2-1-/-
efop2-2-/-
efop3-1-/-
efop3-2-/-
efop4-2-/-
efop4-4-/-
efop1-/- efop2-1-/-
efop1-/- efop2-2-/-
efop1-/- efop3-1-/-
efop1-/- efop3-2-/-
efop1-/- efop4-2-/-
efop1-/- efop4-4-/-
efop2-1-/- efop3-1-/-
efop2-1-/- efop3-2-/-
efop2-2-/- efop3-1-/-
efop2-2-/- efop3-2-/-
efop3-1-/- efop4-2+/-
efop3-1+/- efop4-2-/-
efop3-2-/- efop4-2+/-
efop3-2+/- efop4-2-/-
efop3-2-/- efop4-4+/-
efop3-1+/- efop4-4-/-
efop3-1-/- efop4-4-/-
efop3-2-/- efop4-4+/-
efop3-2+/- efop4-4-/-
efop1-/- efop2-1-/- efop3-1+/-
hyc1+/-
hyc1-/- HYC1prom::HYC1-mCITRINE
hyc2-2+/-
hyc2-3+/-
C
Col0
npg1-2+/-
npg1-2+/- NPG1pro:NPG1-mCITRINE
npg1-2+/- NPG1pro:NPGR2-mCITRINE
#2 #3 #5 #9 #10 #12 #19 #20
GK_480D08-LP/ GK_480D08-RP2
GK_480D08-LP/ o8474
D
Col0
hyc1 +/-
hyc1-/- HYC1pro:HYC1-mCITRINE
GK_767H11-LP/ GK_767H11-RP
GK_767H11-LP/ o8474
GK_767H11-RP/ o8474

Supplemental Figure S4. Characterization of the pollen phenotype of single and multiple *npg*, *hyc* and *efop* mutants. (A) Pollen grains observed using a scanning electronic microscope of self-fertilized *npg1-1+/-, npg1-1+/- npgr1-/-, npgr1-1-/- npgr2-1-/-, npgr1-1-/- npgr2-2-/-, hyc1-/-* expressing *$HYC1_{pro}$:HYC1-mCITR*INE*, efop2-1-/- efop3-1-/-, efop2-1-/- efop3-2-/-, efop2-2-/- efop3-1-/-, efop2-2-/- efop3-2-/-, efop3-1+/- efop4-2-/-, efop3-2-/- efop4-2+/-, efop3-2+/- efop4-2-/-, efop3-2-/- efop4-4+/-,* and *efop3-2+/- efop4-4-/-* plants. Scale bars: 50 µm **(B)** Quantification of the % of normal (blue), deformed (orange) and shrivelled (red) pollen grains of all indicated genotypes. ND is indicated when no quantification of this genotype is available. n indicates the number of pollen grains counted. **(C)** Genotyping of Col-0, *npg1-2* heterozygous plants and *npg1-2* homozygous plants complemented with *$NPG1_{pro}$:NPG1-mCITRINE* (insertion n° 2, 3, 5, 9, 10 and 12) and *$NPG1_{pro}$:NPGR2-mCITRINE* (insertion n° 19 and 20). Upper panel shows amplification of the gene sequence. Lower panel shows amplification of T-DNA border. **(D)** Genotyping of Col-0, *hyc1* heterozygous plants and *hyc1* homozygous plants complemented with *$HYC1_{pro}$:HYC1-mCITRINE* (insertion n° 2). Upper panel shows amplification of gene sequence. Lower panel shows amplification of T-DNA border. Supports Figure 4.

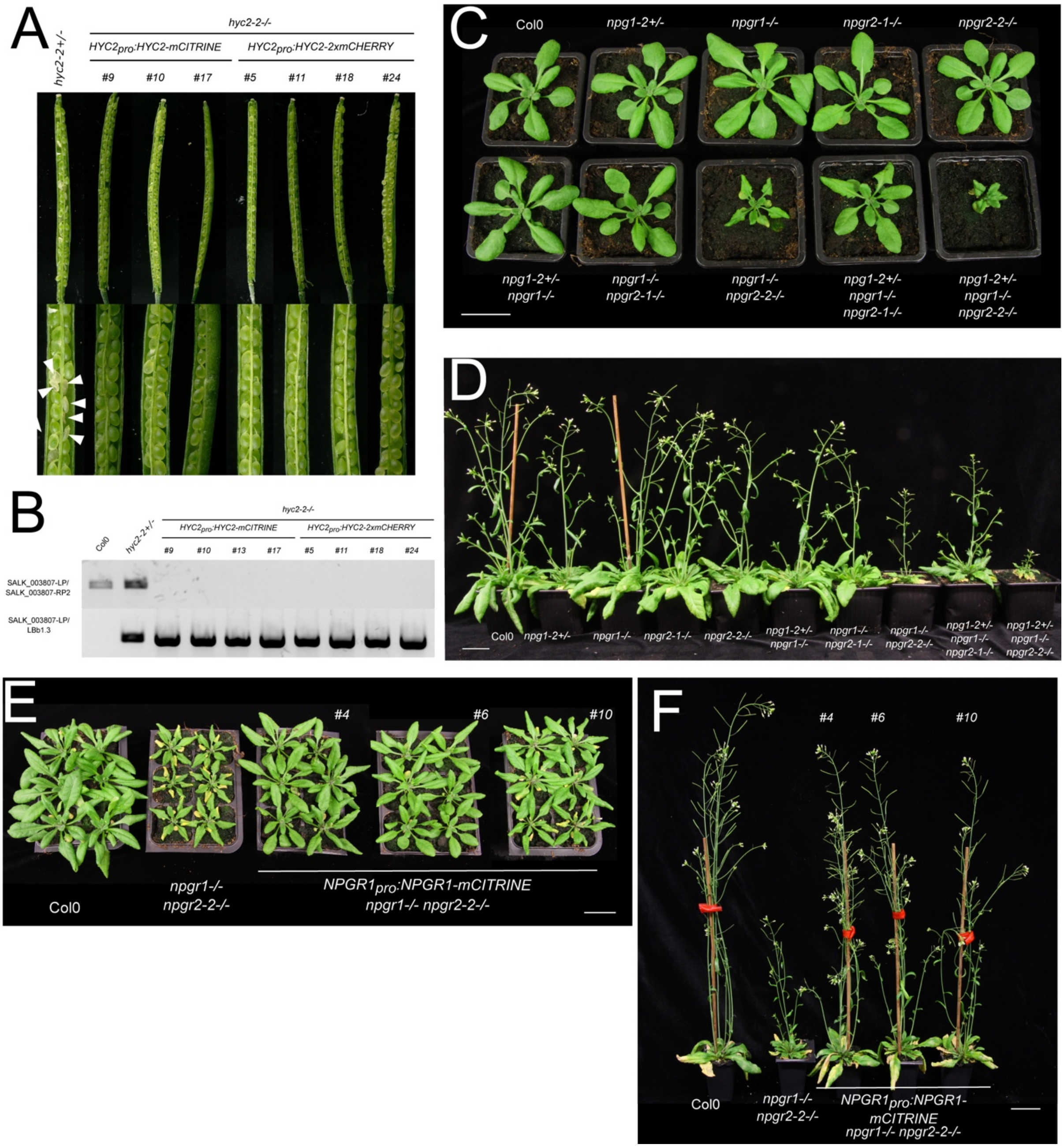

Supplemental Figure S5. Sporophytic phenotypes and complementation of *npg* multiple mutants and *hyc2* single mutant. (A) Opened siliques of self-fertilized *hyc2-2* heterozygous mutant plants and self-fertilized hyc2-2 homozygous plants complemented by the expression of *HYC2*$_{pro}$*:HYC2-mCITRINE* (insertion n°9, 10 and 17) and *HYC2*$_{pro}$*:HYC2-2xmCHERRY* (insertion n°11, 18 and 24). White arrowheads indicate aborted seeds. **(B)** Genotyping of Col-0, *hyc2-2* heterozygous plants and *hyc2-2* homozygous plants complemented with *HYC2*$_{pro}$*:HYC2-mCITRINE* (insertion n°9, 10, 13 and 17) and *HYC2prom:HYC2-2xmCHERRY* (insertion n° 11, 18 and 24). Upper panel shows amplification of gene sequence. Lower panel shows amplification of T-DNA border. **(C)** Twenty-seven-day-old Col-0*, npg1-2+/-, npgr1-/-, npgr2-2-/-, npg1-2+/- npgr1-/-, npgr1-/- npgr2-2-/-* and *npg1-2+/- npgr1-/- npgr2-2-/-* plants. Scale bar: 2 cm **(D)** Forty-one-day-old Col-0, *npg1-2+/-, npgr1-/-, npgr2-2-/-, npg1-2+/- npgr1-/- , npgr1-/- npgr2-2-/-* and *npg1-2+/- npgr1-/- npgr2-2-/-* plants. Scale bar: 2 cm **(E)** Twenty-seven days-old Col-0, *npgr1-/- npgr2-2-/-* and *npgr1-/- npgr2-2-/-* expressing *NPGR1*$_{pro}$*:NPGR1-mCITRINE*. Several independent insertions are shown. Scale bar: 2 cm **(F)** Forty days-old Col-0, *npgr1-/- npgr2-2-/-* and *npgr1-/- npgr2-2-/-* expressing *NPGR1*$_{pro}$*:NPGR1-mCITRINE*. Several independent insertions are shown. Scale bar: 2 cm**.** Supports Figure 5.

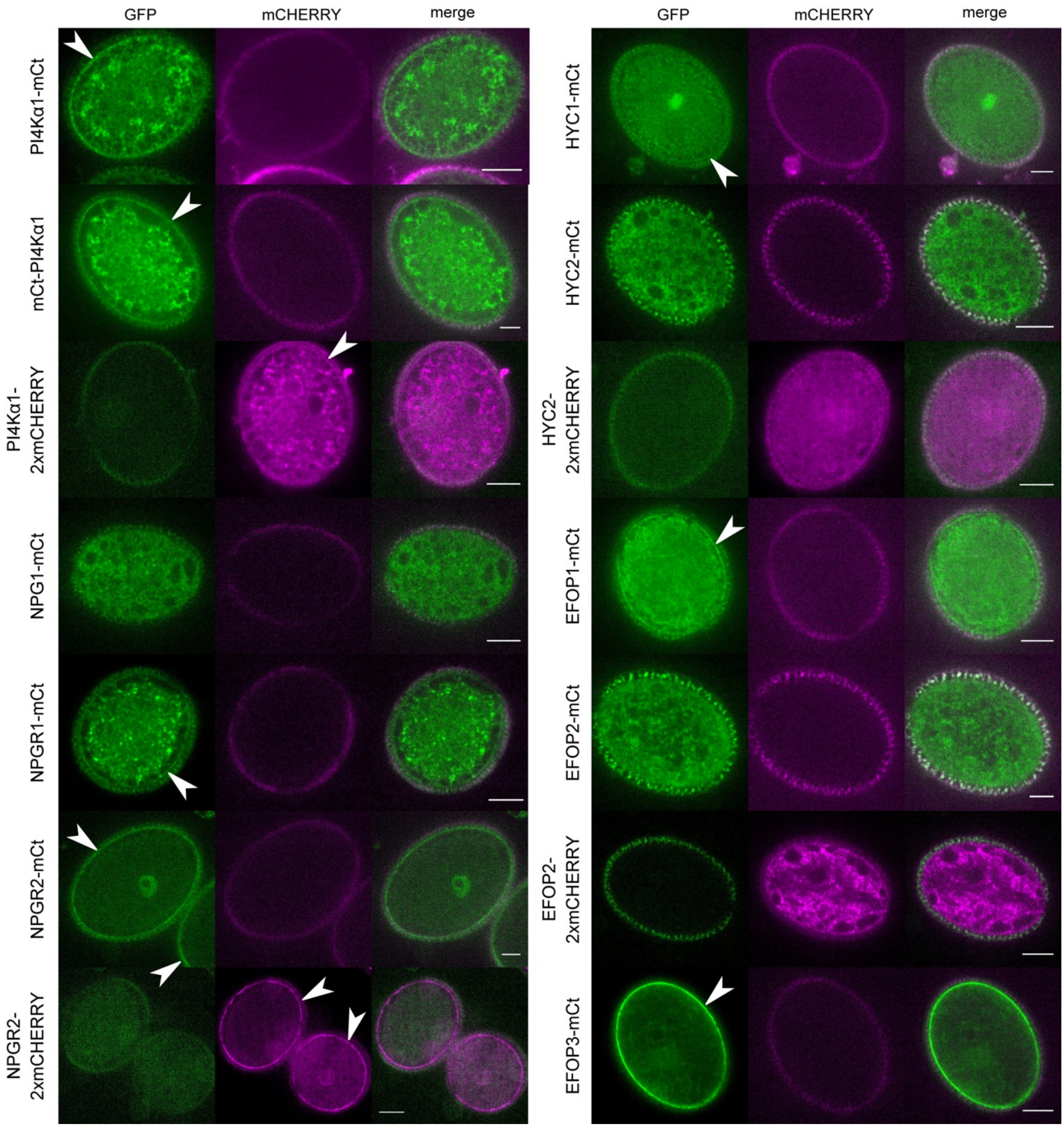

Supplemental Figure S6. PI4Kα1, NPG, HYC and EFOP protein localization in pollen grains. Confocal images of PI4Kα1, NPG1, NPGR1, NPGR2, HYC1, HYC2, EFOP1, EFOP2 and EFOP3 fused to mCITRINE (mCt) or mCHERRY under control of *UBQ10* promoter in pollen grains. Both GFP and mCHERRY fluorescence emission channels are shown to visualise the autofluorescence from the pollen cell wall. White arrowheads indicate plasma membrane labelling. Scale bars: 10 µm. Supports Figure 7.

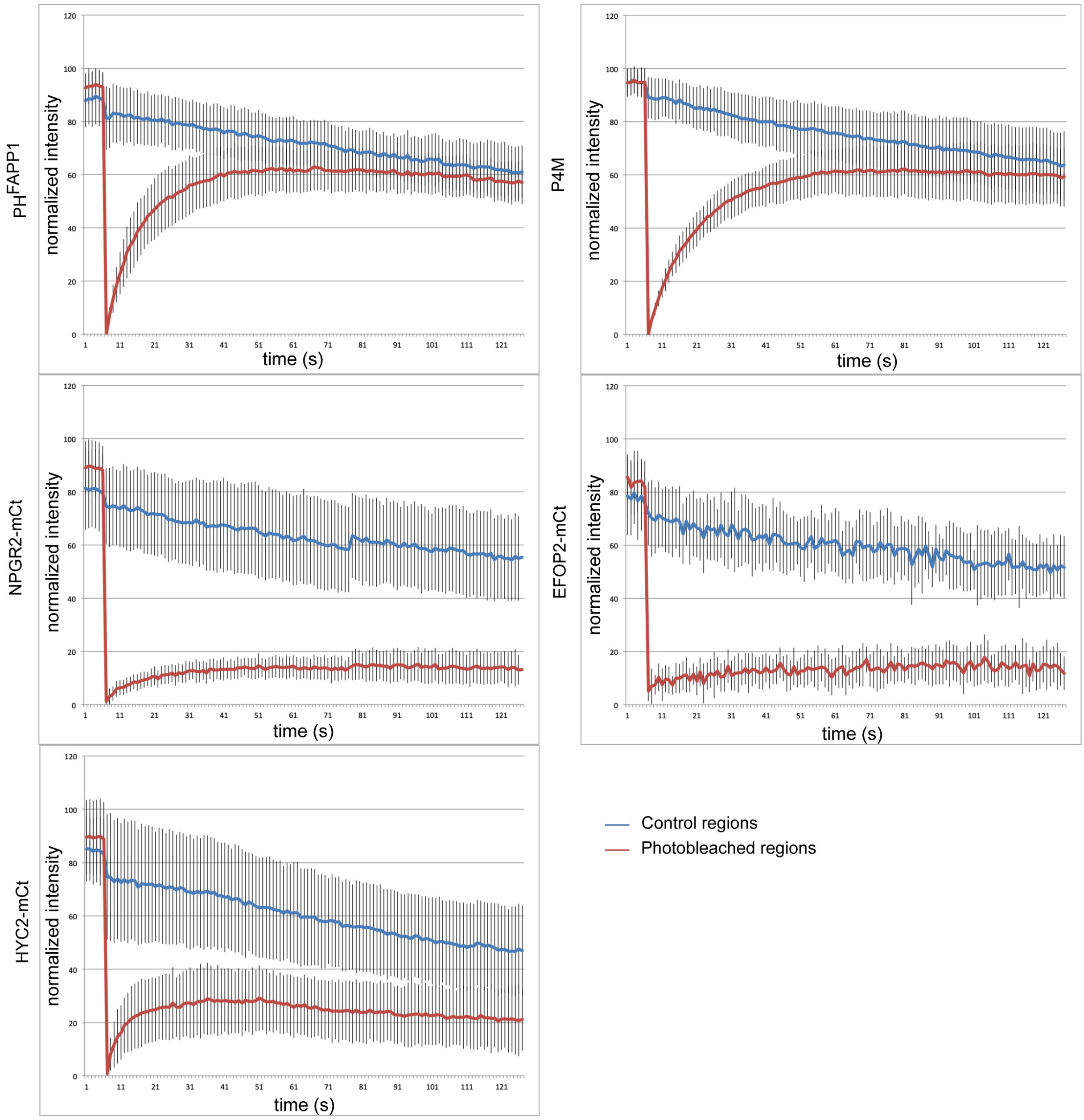

Supplemental Figure S7. FRAP analysis of NPGR2/EFOP2/HYC2 fused with mCITRINE in Arabidopsis root. Graphics presenting signal intensity over time for photobleached (red) and control regions (blue). Standard deviations are shown. The number of zones measured is 37, 32, 30, 13 and 29 for P4M, PH^{FAPP1}, NPGR2-mCt (mCITRINE), EFOP2-mCt and HYC2-mCt, respectively. Supports Figure 8.

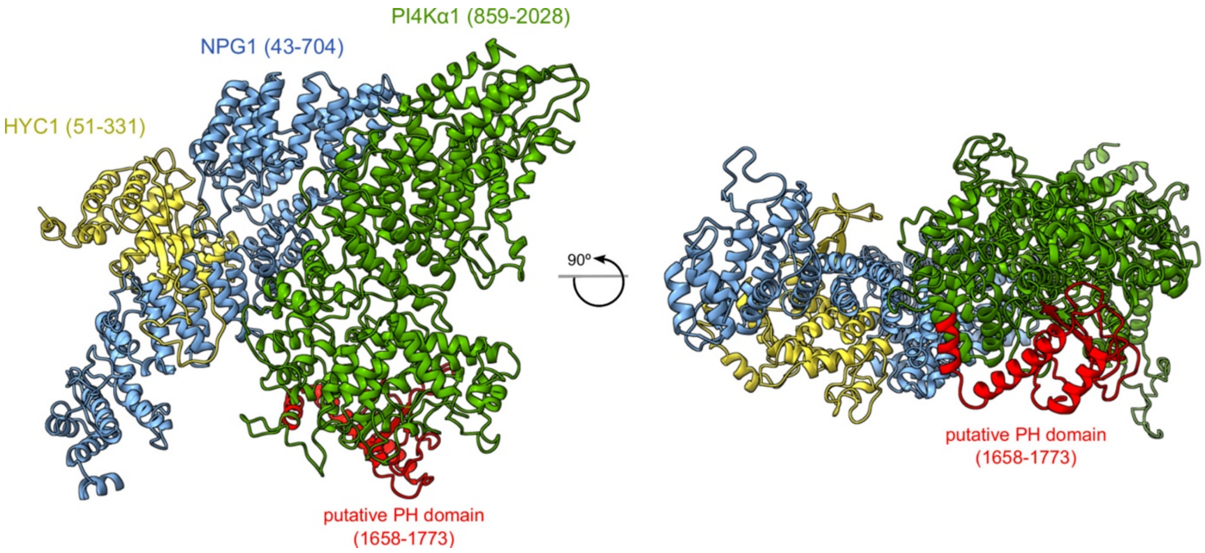

Supplemental Figure S8. Position of the putative PH domain of PI4Kα1. Mapping of the region corresponding to the putative PH domain of PI4Kα1 described by Stevenson et al., 1998; Stevenson-Paulik et al., 2003; Xue et al., 1999. on the heterotrimeric PI4Kα1 complex structure obtained by template-based modelling and protein-protein docking. The putative PH domain overlaps with the helical (cradle) and catalytic domain of PI4Kα1. Supports Figure 10.

	Antibody	References/ catalog #	Company	Host	Concentration used
Primary	anti-GFP	A-6455	ThermoFisher	Rabbit	1/2000
	anti-mCherry	ab167453	Abcam	Rabbit	1/1000
	anti-PI4Ka1	A03 rat #1	Proteogenix	Rat	1/1000
	anti-NPGR2	9094-A01 rabbit #2	Proteogenix	Rabbit	1/2000
Secondary	anti-Rat IgG, HRP conjugate	AP136P	Merck	Goat	1/5000
	anti-Rabbit IgG, HRP conjugate	W4011	Promega	Goat	1/5000

Supplemental Table S1: Antibodies used in this study

	Gene	Locus	Name	T-DNA or CrisPr line	Zygosity	Phenotype observed	Detail
Single Mutant	PI4Kα1	At1g49340	*pi4kα1-1*	GK502_D11	Heterozygous	Male sterility	Insertion in the 1st exon (208bp from ATG) of 26bp followed by two T-DNA in inverted tandem
			pi4kα1-2	FLAG_275H12	Heterozygous	Male sterility	Insertion in the 22nd intron (9087bp from ATG) of 28bp followed by one T-DNA
	NPG1	At2g43040	*npg1-1*	SAIL_262_A01	Heterozygous	Male sterility	Insertion in the 3rd exon (734bp from ATG) of 22bp followed by two T-DNA in inverted tandem
			npg1-2	GK_480D08	Heterozygous	Male sterility	Insertion in the 3rd exon (1585bp from ATG) of 2bp followed by two T-DNA in inverted tandem and 16bp
	NPGR1	At1g27460	*npgr1*	SALK_090514	Homozygous	None	Insertion in the 3rd exon (1152bp from ATG) of 13bp followed by two T-DNA in inverted tandem and 49bp
	NPGR2	At4g28600	*npgr2-1*	CrisPr	Homozygous	None	Insertion of 1 T in the first exon at the 236 pb after the ATG leading a early codon stop and a truncated protein of 82aa
			npgr2-2		Homozygous	None	Deletion of 17 bp leading a early codon stop and a truncated protein of 76aa
	HYC1	At5g21050	*hyc1*	GK_767H11	Heterozygous	Male sterility	Deletion of 10bp and insertion of two T-DNA ininverted tandem at 786bp from ATG
	HYC2	At5g64090	*hyc2-2*	SALK_003807	Heterozygous	Embryo lethality	Insertion of 9bp followed by one T-DNA at 611bp from ATG
			hyc2-3	SALK_040977	Heterozygous	Embryo lethality	Deletion of 30bp and insertion of two T-DNA ininverted tandem at 705bp from ATG
	EFOP1	At5g21080	*efop1*	GK_620B11	Homozygous	None	Insertion in the 9th exon (2118bp from ATG) of 11bp followed by two T-DNA in inverted tandem and 213bp
	EFOP2	At2g41830	*efop2-1*	SALK_128017	Homozygous	None	Insertion in the 6th intron (1976bp from ATG) of one T-DNA
			efop2-2	GK_387B07	Homozygous	None	Insertion in the 11th intron (3829bp from ATG) of 10bp followed by two T-DNA in inverted tandem and 78bp
	EFOP3	At1g05960	*efop3-1*	SALK_121262	Homozygous	None	Insertion in the 1st intron (136bp from ATG) of one T-DNA
			efop3-2	SALK_133976	Homozygous	None	Insertion in the 10th intron (2488bp from ATG) of 58bp followed by two T-DNA in inverted tandem and 28bp
	EFOP4	At5g26850	*efop4-2*	CrisPr	Homozygous	None	Insertion of 1A in position 34 leading a early codon stop and a truncated protein of 42aa
			efop4-4		Homozygous	None	Deletion of 57bp leading to deletion of 19 aa including palmytoylation site and positive charges

Supplemental Table S2: Description of the single and multiple mutants analysed in this study (continued on the next page)

	Gene	Locus	Name	T-DNA or CrisPr line	Zygosity	Phenotype observed
Double Mutant	NPG1<br>NPGR1	At2g43040<br>At1g27460	*npg1-1 npgr1*	SAIL_262_A01 / SALK_090514	NPG1 Heterozygous / NPGR1 Homozygous	About 10% of shrivelled pollen
			npg1-2 npgr1	GK_480D08 / SALK_090514		
	NPGR1<br>NPGR2	At1g27460<br>At4g28600	*npgr1 npgr2-1*	SALK_090514 / CrisPr	Double Homozygous	About 10% of shrivelled pollen
			npgr1 npgr2-2	SALK_090514 / CrisPr		Weak pollen phenotype / Growth phenotype
	EFOP1<br>EFOP2	At5g21080<br>At2g41830	*efop1 efop2-1*	GK_620B11 / SALK_128017	Double Homozygous	None
			efop1 efop2-2	GK_620B11 / GK_387B07		
	EFOP1<br>EFOP3	At5g21080<br>At1g05960	*efop1 efop3-1*	GK_620B11 / SALK_121262	Double Homozygous	None
			efop1 efop3-2	GK_620B11 / SALK_133976		
	EFOP1<br>EFOP4	At5g21080<br>At5g26850	*efop1 efop4-2*	GK_620B11 / CrisPr	Double Homozygous	None
			efop1 efop4-4	GK_620B11 / CrisPr		
	EFOP2<br>EFOP3	At2g41830<br>At1g05960	*efop2-1 efop3-1*	SALK_128017 / SALK_121262	Double Homozygous	From 70 to 90% of deformed or shrivelled pollen grains
			efop2-1 efop3-2	SALK_128017 / SALK_133976		
			efop2-2 efop3-1	GK_387B07 / SALK_121262		
			efop2-2 efop3-2	GK_387B07 / SALK_133976		
	EFOP2<br>EFOP4	At2g41830<br>At5g26850	*efop2-1 efop4-1*	SALK_128017 / SAIL_642H01	Double Homozygous	None
			efop2-2 efop4-1	GK_387B07 / SAIL_642H01		
	EFOP3<br>EFOP4	At1g05960<br>At5g26850	*efop3-1 efop4-2*	SALK_121262 / CrisPr		About 50% of deformed or shrivelled pollen grains
			efop3-1 efop4-4	SALK_121262 / CrisPr		
			efop3-2 efop4-1	SALK_133976 / SAIL_642H01		About 50% of shrivelled pollens when efop3-2 is homozygous
			efop3-2 efop4-2	SALK_133976 / CrisPr		About 50% of deformed or shrivelled pollen grains
			efop3-2 efop4-4	SALK_133976 / CrisPr		
Triple Mutant	NPG1<br>NPGR1<br>NPGR2	At2g43040<br>At1g27460<br>At4g28600	*npg1-2 npgr1 npgr2-1*	SAIL_262_A01 / SALK_090514 / CrisPr	NPG1 Heterozygous / NPGR1 and NPGR2	Dwarfism
			npg1-2 npgr1 npgr2-2	SAIL_262_A01 / SALK_090514 / CrisPr		
	EFOP1<br>EFOP2<br>EFOP3	At5g21080<br>At2g41830<br>At1g05960	*efop1 efop2-1 efop3-1*	GK-620B11 / SALK_128017 / SALK_121262	Triple homozygous	None

Supplemental Table S2: Description of the single and multiple mutants analysed in this study

Name	Full name	Background	Entry vectors			Destination vector	Use	Number of independant transformation events analyzed	Number of characterized lines (T3 homozygous - single insertion)	Figure	Sources
			pDONR P4-P1R	pDONR221	pDONR P2R-P3						
**Lti6b-mCITRINE / Lti6b-mCt**	2x35Sprom:Lti6b-FRB-CITRINE	Col0	2X35Spro	Lti6b-FRB/pDONR 207	mCITRINE	pB7m34GW	Coimmunoprecipitation	16	3	1	This study
**NPGR2-mCITRINE / NPGR2-mCt**	UBQ10prom:NPGR2-mCITRINE	Col0	UBQ10pro	NPGR2(CDS)	mCITRINE	pB7m34GW	Coimmunoprecipitation, mass spectrometry, antibody test, Confocal microscopy, FRAP, PAO treatment	22	2	1, 7, 8, 9, S1, S6, S7	This study
**mCITRINE-PI4Kα1 / mCt-PI4Kα1**	UBQ10prom:mCITRINE-PI4Kα1	Col0	UBQ10pro	mCITRINE	PI4Kα1(CDS)	pB7m34GW	mass spectrometry, Confocal microscopy	15	4	1, 7, S6	This study
**mCITRINE-NES-mCITRINE**	2x35Sp::mCITRINE-NES-mCITRINE	Col0	2X35Spro	mCITRINE	NES-mCITRINE	pB7m34GW	mass spectrometry	20	3	1	This study
**myristoylation-2XmCITRINE**	2xp35S::myrimCITRINE-mCITRINE	Col0	2X35Spro	myri-mCITRINE	mCITRINE	pB7m34GW	mass spectrometry			1	Jaillais et al., 2011
**HYC2-mCITRINE / HYC2-mCt**	UBQ10prom:HYC2-mCITRINE	Col0	UBQ10pro	HYC2(CDS)	mCITRINE	pB7m34GW	Coimmunoprecipitation, Confocal microscopy, FRAP	23	2	1, 7, 8, S6, S7	This study
**EFOP2-mCITRINE / EFOP2-mCt**	UBQ10prom:EFOP2-mCITRINE	Col0	UBQ10pro	EFOP2(CDS)	mCITRINE	pB7m34GW	Coimmunoprecipitation, Confocal microscopy, FRAP, PAO treatment	24	4	1, 7, 8, 9, S6, S7	This study
**PI4Kα1-mCITRINE / PI4Kα1-mCt**	UBQ10prom:PI4Kα1-mCITRINE	Col0	UBQ10pro	PI4Kα1(CDS)	mCITRINE	pB7m34GW	Antibody test, Confocal microscopy	24	3	7, S1, S6	This study
**PI4Kα1-2XmCHERRY**	UBQ10prom:PI4Kα1-2xmCHERRY-4xmyc	Col0	UBQ10pro	PI4Kα1(CDS)	2xmCHERRY-4xmyc	pH7m34GW	Antibody test, Confocal microscopy	22	3	7, S1, S6	This study
**NPGR1-mCITRINE / NPGR1-mCt**	UBQ10prom:NPGR1-mCITRINE	Col0	UBQ10pro	NPGR1(CDS)	mCITRINE	pLOK180_pR7 m34g	Antibody test, Confocal microscopy	22	3	7, S1, S6	This study
**HYC2-2XmCHERRY**	UBQ10prom:HYC2-2xmCHERRY-4xmyc	Col0	UBQ10pro	HYC2(CDS)	2xmCHERRY-4xmyc	pH7m34GW	Confocal microscopy	24	5	S6	This study
**EFOP2-2XmCHERRY**	UBQ10prom:EFOP2-2xmCHERRY-4xmyc	Col0	UBQ10pro	EFOP2(CDS)	2xmCHERRY-4xmyc	pH7m34GW	Confocal microscopy	23	3	S6	This study
**PI4Kα1pro:PI4Kα1-3'UTR**		*pi4ka1-1* GK502D11	PI4Kα1pro	PI4Kα1(CDS)	PI4Kα1-3'UTR	pB7m34GW	Complementation	19	5 complementing lines analyzed in T3	3, S3	This study
**PI4Kα1pro:PI4Kα1-mCITRINE**		*pi4ka1-1* GK502D11	PI4Kα1pro	PI4Kα1(CDS)	mCITRINE	pB7m34GW	Complementation	24	0 complementing line	na	This study
**PI4Kα1pro:mCITRINE-PI4Kα1**		*pi4ka1-1* GK502D11	PI4Kα1pro	mCITRINE	PI4Kα1(CDS)	pB7m34GW	Complementation	27	0 complementing line	na	This study
**PI4Kα1pro:PI4Kα1-2xmCHERRY**	PI4Kα1prom:PI4Kα1-2xmCHERRY-4xMyc	*pi4ka1-1* GK502D11	PI4Kα1pro	PI4Kα1(CDS)	2xmCHERRY-4xmyc	pH7m34GW	Complementation	22	0 complementing line	na	This study
**PI4Kα1pro:PI4Kα1-6xHA**		*pi4ka1-1* GK502D11	PI4Kα1pro	PI4Kα1(CDS)	6xHA	pK7m34GW	Complementation	18	0 complementing line	na	This study
**PI4Kα1pro:Flag-PI4Kα1**		*pi4ka1-1* GK502D11	PI4Kα1pro	Flag-Gslinker	PI4Kα1(CDS)	pB7m34GW	Complementation	22	0 complementing line	na	This study
**NPG1pro:NPG1-mCITRINE**		*npg1-2 +/-* GK_480D08	NPG1pro	NPG1(CDS)	mCITRINE	pB7m34GW	Complementation	18	6 complementing lines analyzed in T3	S4	This study
**NPG1pro:NPGR2-mCITRINE**		*npg1-2 +/-* GK_480D08	NPG1pro	NPGR2(CDS)	mCITRINE	pB7m34GW	Complementation	24	6 complementing lines analyzed in T3	S4	This study
**NPGR1pro:NPGR1-mCITRINE**		*npgr1 npgr2-2* SALK_090514/CrisPR	NPGR1pro	NPGR1(CDS)	mCITRINE	pB7m34GW	Complementation	21	5 complementing lines analyzed in T3	S5	This study
**HYC1pro:HYC1-mCITRINE**		*hyc1* GK_767H11	HYC1pro	HYC1(CDS)	mCITRINE	pB7m34GW	Complementation	12	1 complementing lines analyzed in T3	S4	This study
**HYC2pro:HYC2-mCITRINE**		*hyc2-2* SALK_003807	HYC2pro	HYC2(CDS)	mCITRINE	pB7m34GW	Complementation	25	4 complementing lines analyzed in T3	5, S5	This study
**HYC2pro:HYC2-2xmCHERRY**	HYC2pro:HYC2-2xmCHERRY-4xmyc	*hyc2-2* SALK_003807	HYC2pro	HYC2(CDS)	2xmCHERRY-4xmyc	pH7m34GW	Complementation	24	4 complementing lines analyzed in T3	5, S5	This study
**HYC1-mCITRINE / HYC1-mCt**	UBQ10pro:HYC1-mCITRINE	Col0	UBQ10pro	HYC1(CDS)	mCITRINE	pB7m34GW	Confocal microscopy	23	4	6, S6	This study
**NPG1-mCITRINE / NPG1-mCt**	UBQ10pro:NPG1-mCITRINE	Col0	UBQ10pro	NPG1(CDS)	mCITRINE	pB7m34GW	Confocal microscopy	24	4	6, S6	This study
**EFOP1-mCITRINE / EFOP1-mCt**	UBQ10pro:EFOP1-mCITRINE	Col0	UBQ10pro	EFOP1genomic	mCITRINE	pB7m34GW	Confocal microscopy	23	4	6, S6	This study
**EFOP3-mCITRINE / EFOP3-mCt**	UBQ10pro:EFOP3-mCITRINE	Col0	UBQ10pro	EFOP3(CDS)	mCITRINE	pB7m34GW	Confocal microscopy	23	4	6, S6	This study
**Lti6b-2XmCHERRY**	UBQ10pro:Lti6b-2xmCHERRY-4xmyc	Col0	UBQ10pro	Lti6b/pDONR207	2xmCHERRY-4xmyc	pH7m34GW	Confocal microscopy			6	Platre et al., 2018
**NPGR2-2XmCHERRY**	UBQ10pro:NPGR2-2xmCHERRY-4xmyc	Col0	UBQ10pro	NPGR2(CDS)	2xmCHERRY-4xmyc	pH7m34GW	Confocal microscopy	9	4	6, S6	This study
**PHFAPP1 / P5Y**	UBQ10pro:mCITRINE-1xPHFAPP1 (P5Y)	Col0	UBQ10pro	mCITRINE	PH(FAPP1)	pB7m34GW	FRAP, PAO treatment, microRNA			7, 6, 9, S7	Simon et al., 2014 NASC# N210560
**PHFAPP1-E50A**	UBQ10pro:mCITRINE-1xPHFAPP1 E50A	Col0	UBQ10pro	mCITRINE	PH(FAPP1-E50A)	pB7m34GW	microRNA			6	Simon et al., 2016
**P4M**	UBQ10pro:mCITRINE-P4MSidM	Col0	UBQ10pro	mCITRINE	P4M	pB7m34GW	FRAP, microRNA			7, 6, S7	Simon et al., 2016 NASC# N210734
**EFOP2-7Q**	UBQ10pro:EFOP2-7Q-GFP	Col0		EFOP2-7Q		pK7FWG2	Confocal microscopy	14	14	9	This study
**EFOP2-CC**	UBQ10pro:EFOP2C20SC23S-mCITRINE	Col0	UBQ10pro	EFOP2C20SC23S	mCITRINE	pB7m34GW	Confocal microscopy	22	3	9	This study
**PI4Kα1pro:PI4Kα1-mCITRINE-Lti6b**	PI4Kα1pro:PI4Kα1-mCITRINE-3xSAG-Lti6b	*pi4ka1-1* GK502D11	PI4Kα1pro	PI4Kα1(CDS)	mCITRINE-3xSAG-Lti6b	pB7m34GW	Complementation	19	0 complementing lines	10	This study
**PI4Kα1pro:PI4Kα1-mCITRINE-Lti6b**	PI4Kα1pro:PI4Kα1-mCITRINE-3xSAG-Lti6b	*npg1-2 +/-* GK_480D08	PI4Kα1pro	PI4Kα1(CDS)	mCITRINE-3xSAG-Lti6b	pB7m34GW	Complementation	19	1 complementing line analyzed in T3	10	This study
**UBQ10pro:PI4Kα1microRNA2**		Col0	UBQ10pro	PI4Kα1microRNA2	mock	pH7m34GW	Artificial micro RNA	10	10	5	This study
**UBQ10pro:PI4Kα1microRNA3**		Col0	UBQ10pro	PI4Kα1microRNA3	mock	pH7m34GW	Artificial micro RNA	20	6	5	This study
**UBQ10pro:XVE>>PI4Kα1microRNA2**		Col0	UBQ10pro:XVE	PI4Kα1microRNA2	mock	pH7m34GW	Artificial micro RNA	23	4	6	This study

Supplemental Table S3: Transgenic lines

Name	Source	
Empty gateway entry vector: pDONR221	thermofisher	cat# 12536017
Empty gateway entry vector: pDONR P4P1R	thermofisher	cat# 12537023
Empty gateway entry vector: pDONR P2RP3	thermofisher	cat# 12537023
Empty gateway destnation vector: pB7m34GW	Karimi et al., 2007	N/A
Empty gateway destnation vector: pH7m34GW	Karimi et al., 2007	N/A
Empty gateway destnation vector: pLOK180_pR7m34g	L. Kalmbach and M. Barberon	N/A
Empty gateway destnation vector:pK7FWG2	Karimi et al., 2002	N/A
Entry vector: UBQ10prom/pDONR P4-P1r	Jaillais et al., 2011	NASC N2106315
Entry vector: 2X35S/pDONR P4-P1r	Mar Marquès-Bueno, M. et al. 2016	NASC N2106316
Entry vector: mCITRINE/pDONR 221	Simon et al., 2014	NASC N2106287
Entry vector: mCITRINE/pDONR P2R-P3	Jaillais et al., 2011	NASC N2106288
Entry vector: 6xHA/pDONR P2R-P3	Jaillais et al., 2011	N/A
Entry vector: 2xmCHERRY-4xmyc/pDONR P2R-P3	Simon et al., 2014	NASC N2106292
Lti6b/pDONR207	Elsayad et al., 2016	N/A
pEGFP-FRB (Gift of Klaus Hahn)	Gift of Klaus Hahn	plasmid addgene #25919
UBQ10prom-XVE/pDONR P4-P1r	Siligato et al., 2016	N/A

Supplemental Table S4: Published vectors used in this study

	Name	Sequence	Primer Sequence (5'-3'; forward then reverse)	Modifications	Template	Destination vector
Genes / reporters	PI4Kα1	Coding sequence from ATG to Stop	GGGGACAAGTTTGTACAAAAAAGCAGGCTTAACCATGGAGGCACTGACGGAGCTTTGTG	B1-Kozak sequence	cDNA from seedlings	pDONR 221
			GGGGACCACTTTGTACAAGAAAGCTGGGTACTTCTCGATGCCTTGTTGCAAATACTG	B2-stop mutated		
			GGGGACAAGTTTGTACAAAAAAGCAGGCTTAACCATGGAGGCACTGACGGAGCTTTGTG	B1-Kozak sequence		pDONR P2R-P3
			GGGGACCACTTTGTACAAGAAAGCTGGGTATTACTTCTCGATGCCTTGTTGCAAATACTG	B2-STOP		
			GGGGACAGCTTTCTTGTACAAAGTGGCTATGGAGGCACTGACGGAGCTTTGTGAC	B2R		
			GGGGACAACTTTGTATAATAAAGTTGCTTACTTCTCGATGCCTTGTTGCAAATACTG	B3		
	NPG1	Coding sequence from ATG to Stop	GGGGACAAGTTTGTACAAAAAAGCAGGCTTAACCATGCTCGGGAATCAATCCG	B1-Kozak sequence	cDNA from seedlings	pDONR221
			GGGGACCACTTTGTACAAGAAAGCTGGGTATTAAAGAATGGTTGAGAAGCTTTCAA	B2-stop mutated		
	NPGR1	Coding sequence from ATG to Stop	GGGGACAAGTTTGTACAAAAAAGCAGGCTTAACCATGTTGTGTGCTTGTTCAGGC	B1-Kozak sequence	cDNA from seedlings	pDONR 221
			GGGGACCACTTTGTACAAGAAAGCTGGGTATCAAATGAAACTCTGTACCGGAGC	B2-stop mutated		
	NPGR2	Coding sequence from ATG to Stop	GGGGACAAGTTTGTACAAAAAAGCAGGCTTAACCATGAAGAACAGCGAGATTAGGCC	B1-Kozak sequence	cDNA from seedlings	pDONR 221
			GGGGACCACTTTGTACAAGAAAGCTGGGTATCATCTGAATGGCTCCACTGG	B2-stop mutated		
	HYC1	Coding sequence/genomic sequence from ATG to Stop	GGGGACAAGTTTGTACAAAAAAGCAGGCTTAACCATGTCTGATTCTTCCTCAAGCCATG	B1-Kozak sequence	genomic DNA	pDONR 221
			GGGGACCACTTTGTACAAGAAAGCTGGGTAAACGGAGAGAACGGTGTCGTTTGATAGCTC	B2-stop mutated		
	HYC2	Coding sequence/genomic sequence from ATG to Stop	GGGGACAAGTTTGTACAAAAAAGCAGGCTTAACCATGGACTTCTCCGTCAAACCTTCCGG	B1-Kozak sequence	genomic DNA	pDONR 221
			GGGGACCACTTTGTACAAGAAAGCTGGGTACTTTGATGCCAGAAGAATCTCAGG	B2-stop mutated		
	ELP1	Genomic sequence from ATG to Stop	GGGGACAAGTTTGTACAAAAAAGCAGGCTTAACCATGGGGGTTGTTTCAAGAACC	B1-Kozak sequence	genomic DNA	pDONR 221
			GGGGACCACTTTGTACAAGAAAGCTGGGTACTAATCAAGAGGAACCAACGGC	B2-STOP		
	ELP2	Coding sequence from ATG to Stop	GGGGACAAGTTTGTACAAAAAAGCAGGCTTAACCATGAGCGCAGTCTCGGGTG	B1-Kozak sequence	cDNA from seedlings	pDONR 221
			GGGGACCACTTTGTACAAGAAAGCTGGGTAACAGCCAGCAGCTTTAAGGAAG	B2-stop mutated		
	ELP3	Coding sequence from ATG to Stop	GGGGACAAGTTTGTACAAAAAAGCAGGCTTAACCATGGGGGTTATGTCCAGACG	B1-Kozak sequence	cDNA from seedlings	pDONR 221
			GGGGACCACTTTGTACAAGAAAGCTGGGTATTAACACCCGGCTGCTTTC	B2-STOP		
	NES-mCitrine	Addition of NES sequence uptream of mCitrine on mCITRINE/pDONR P2RP3 entry vector	/5PHOS/GTTAGAGGAGCTGGAGCTAGACGAACAGCAAGGAGGAATGGTGAGCAAGGGCGAGGAGC	5'Phosphorylated	mCITRINE/pDONR P2RP3 entry vector from Jaillais et al., 2011	pDONR P2R-P3
			/5phos/TTCTTTTGAAGGTCAACCAAATTCGGAGCCACTTTGTACAAGAAAGTTGAACGAGAAACG	5'Phosphorylated		
	mCITRINE-3xSAG-Lti6b	Lti6b with homology to mCITRINE and include the 3xSAG linker sequence	AGTGCTGGTAGTGCTGGTAGTGCTGGTAGTACAGCCACTTTCGTAGAGATTATTCTTGC		Lti6b/pDONR207 from Elsayad et al., 2016	pDONR P2R-P3
			GTATAATAAAGTTGCTTACTTGGTGATGATATAAAGAGCGTAAAGGATTCCGGGAAG			
		mCITRINE with homology to Lti6b and include 3xSAG linker sequence	CTTTATATCATCACCAAGTAAGCAACTTTATTATACAAAGTTGGCA		mCITRINE/pDONR P2RP3 entry vector from Jaillais et al., 2011	
			ACTACCAGCACTACCAGCACTACCAGCACTCTTGTACAGCTCGTCCATGCCGAGAGTG			
	FRB-Lti6b	Lti6b with homology to FRB linker and pDONR207	TACCCAGCTTTCTTGTACAAAGTG		Lti6b/pDONR207 from Elsayad et al., 2016	pDONR 207
			CTTGGTGATGATATAAAGAGCG			
		FRB linker with homology to Lti6b and pDONR207	CTTTATATCATCACCAAGGCGGGAGCAGATAGTGCT		pEGFP-FRB (plasmid addgene #25919 Gift of Klaus Hahn)	
			TACAAGAAAGCTGGGTAACTAGTCTTTGAGATTCGTCGG			
Promoters	PI4Kα1prom	1316bp upstream ATG with homology to pDONR P4-P1r	TTTGTATAGAAAAGTTGCTGTTAGGTTGATTCTCCTATCCTGGGTTTG	B4	genomic DNA	pDONR P4-P1r
			AACTTTTTTGTACAAACTTGCCTCCGATTAAACAAAAAAAACAACGTTACG	B1		
		pDONR P4-P1r with homology to pPI4Kα1	TTTTGTTTAATCGGAGGCAAGTTTGTACAAAAAAGTTGAACGAG	B4	pDONR P4-P1r	
			AGGATAGGAGAATCAACCTAACAGCAACTTTTCTATACAAAGTTGGC	B1		
	NPG1prom	3983bp uspstream ATG with homology to pDONR P4-P1r	ACTTTGTATAGAAAAGTTGCTTATAGTTATGAAACTTAATGATTAGCGAGTTG	B4	genomic DNA	pDONR P4-P1r
			TTCAACTTTTTTGTACAAACTTGCTGTTCTTCACCTTTTAGACTAAACCC	B1		
		pDONR P4-P1r with homology to pNPG1	AGTCTAAAAGGTGAAGAACAGCAAGTTTGTACAAAAAAGTTGAACGAGAAACG	B4	pDONR P4-P1r	
			TCATTAAGTTTCATAACTATAAGCAACTTTTCTATACAAAGTTGGCATTATAAAAAAGC	B1		
	NPGR1prom	1226bp upstream ATG with homology to pDONR P4-P1r	ACTTTGTATAGAAAAGTTGCTGGAAAGGAGAAACTAGGAGAGACGTCGCTATTTG	B4	genomic DNA	pDONR P4-P1r
			CGTTCAACTTTTTTGTACAAACTTGCCTTCTCCTCTTTCTCCTCACAAACGCGGAAATGC	B1		
		pDONR P4-P1r with homology to pNPGR1	GTGAGGAGAAAGAGGAGAAGGCAAGTTTGTACAAAAAAGTTGAACGAGAAACG	B4	pDONR P4-P1r	
			CTCTCCTAGTTTCTCCTTTCCAGCAACTTTTCTATACAAAGTTGGC	B1		
	HYC1prom	1212bp upstream ATG with homology to pDONR P4-P1r	GTATAGAAAAGTTGCTGAAAAGCCAGTCAGCGTCCGATGTAGCCCG	B4	genomic DNA	pDONR P4-P1r
			TTTGTACAAACTTGCTTTGGGGAAGATGATGATCGCTTCTGAG	B1		
		pDONR P4-P1r with homology to pHYC1	GCTGACTGGCTTTTCAGCAACTTTTCTATACAAAGTTGGC	B4	pDONR P4-P1r	
			CATCATCTTCCCCAAAGCAAGTTTGTACAAAAAAGTTGAACGAG	B1		
	HYC2prom	1430bp upstream ATG with homology to pDONR P4-P1r	GTATAGAAAAGTTGCTTGTGTGGGATTTTGATTCTTCTTCTC	B4	genomic DNA	pDONR P4-P1r
			TTTGTACAAACTTGCGGGAATTGAGTCTGACTTGAGAGAC	B1		
		pDONR P4-P1r with homology to pHYC2	AGTCAGACTCAATTCCCGCAAGTTTGTACAAAAAAGTTGAACGAG	B4	pDONR P4-P1r	
			TCAAAATCCCACACAAGCAACTTTTCTATACAAAGTTGGC	B1		
	ELP1prom	2594bp upstream ATG with homology to pDONR P4-P1r	TGTATAGAAAAGTTGCTAATCCAATGACTAGAAAAGTCTACAC	B4	genomic DNA	pDONR P4-P1r
			TACGTTTCTCGTTCAACTTTTTTGTACAAACTTGCCGATCTATCTCTCTTCC	B1		
		pDONR P4-P1r with homology to pELP1	CGAATATTCGCGGAAGAGAGATAGATCGGCAAGTTTGTACAAAAAAGTTG	B4	pDONR P4-P1r	
			GACTTTTCTAGTCATTGGATTAGCAACTTTTCTATACAAAGTTGGC	B1		
	ELP3prom	2411bp upstream ATG with homology to pDONR P4-P1r	TATAGAAAAGTTGCTTGCCCACACGCAGGATCGAACTACGGACCTTC	B4	genomic DNA	pDONR P4-P1r
			CAACTTTTTTGTACAAACTTGCTTTCTTCTCCGCCTAATATCC	B1		
		pDONR P4-P1r with homology to pELP3	TAGGCGGAGAAGAAAGCAAGTTTGTACAAAAAAGTTGAACGAGAAACG	B4	pDONR P4-P1r	
			CGATCCTGCGTGTGGGCAAGCAACTTTTCTATACAAAGTTGGC	B1		
3'UTR	PI4Kα1 3'UTR	395bp dowstream STOP codon	GGGGACAGCTTTCTTGTACAAAGTGGCTGAAGAAGAAGAGGGAACTTAATGATATCCG	B2R	genomic DNA	pDONR P2R-*P3*
			GGGGACAACTTTGTATAATAAAGTTGCGAATACAAATACCTCTACATGTTAATAG	B3		

Supplemental Table S5: Primers used for cloning into gateway entry vectors

Directed mutagenesis

Name	Mutation	Primer Sequence (5'-3'; forward then reverse)
ELP1noSTOP/pDONR 221	Revertion of the STOP codon (TAG-->TTG leu) in ELP1gwSTOP/p221	cctcttgattTgtacccagctttcttgtacaaagtt
		agctgggtacAaatcaagaggaaccaacggcttggc
ELP3noSTOP/pDONR 221	Deletion of the stop (TAT)	gcagccgggTGTtacccagctttcttgtacaaagttgg
		gaaagctgggtaACAcccggctgctttcaagaatttatc
ELP2-CC/pDONR 221	Site directed mutagenesis C20 and C23 to S (TGT->AGT and TGC->AGC) to mutate the palmytoylation site	tgtggtagccttAgtattcttAgccctgcgcttcgtgcaaggtctagacag
		acgaagcgcagggcTaagaatacTaaggctaccacaaacaggcaaaacttgcctc
ELP2-7Q/pDONR 221	site directed mutagenesis R29Q (AGG-->CAA) on ELP2	attctttgccctgcgctt**CGT**gca**CAG**tct**AGA**cagcctgtgaagaggtac
		cttgtacctcttcacaggctgTCTagaCTGtgcACGaagcgcagggcaaag
	site directed mutagenesis R27Q (CGT-->CAA)and R31Q (AGA-->CAA) on ELP2 to use on ELP2-R29Q	attctttgccctgcgcttCAAgcaCAGtctCAAcagcctgtgaagaggtac
		cttgtacctcttcacaggctg**TTG**aga**CTG**tgc**TTG**aagcgcagggcaaag
	site directed mutagenesis K35Q (AAG-->CAG)and K38Q (AAG-->CAG) on ELP2 to use on ELP2-3Q (R27QR29QR31Q)	CAGtctCAAcagcctgtgCAGAGGtacCAGAAGctcattgctgagatttttcc
		aaaaatctcagcaatgagCTTCTGgtaCCTCTGcacaggctgTTGagaCTGtgc
	site directed mutagenesis R36Q (AAG-->CAG) and K39Q (AAG-->CAG) on ELP2 to use on ELP2-5Q (R27QR29QR31QK35QK38Q)	CAGtctCAAcagcctgtgCAGCAGtacCAGCAGctcattgctgagatttttcc
		aaaaatctcagcaatgagCTGCTGgtaCTGCTGcacaggctgTTGagaCTGtgc

Supplemental Table S6: Primers used for site directed mutagenesis

Gene mutated	Primer Name	Description	Primer Sequence (5'-3'; forward then reverse)
NPGR2	NPGR2-BsF	To obtain new Crispr lines using the egg especific promoter or 35S promoter vectors from Wang et al. Genome biology 2015. Cloning using Golden Gate reaction. Forward primer to clone a Single gRNA1 NPGR2 using pCBC-DT1T2 vector as a template. 4 primers PCR with primers NPGR2-F0 and NPGR2-R0 and NPGR2-BsR. Amplicon size around 620 bp. This primer contains de gRNA sequence and the BSAI cutting site. Original primer name in the protocol: DT1-BsF.	ATATATGGTCTCGATTGTTACGTAAATGCAACTTCTCGTT
	NPGR2-F0	To obtain new Crispr lines using the egg especific promoter or 35S promoter vectors from Wang et al. Genome biology 2015. Cloning using Golden Gate reaction. Forward primer to clone a Single gRNA1 NPGR2 using pCBC-DT1T2 vector as a template. 4 primers PCR with primers NPGR2-BsF and NPGR2-R0 and NPGR2-BsR. Amplicon size around 620 bp. This primer contains de gRNA sequence. Original primer name in the protocol: DT1-F0.	TGTTACGTAAATGCAACTTCTCGTTTTAGAGCTAGAAATAGC
	NPGR2-R0	To obtain new Crispr lines using the egg especific promoter or 35S promoter vectors from Wang et al. Genome biology 2015. Cloning using Golden Gate reaction. Reverse primer to clone a Single gRNA2 NPGR2 using pCBC-DT1T2 vector as a template. 4 primers PCR with primers NPGR2-BsF and NPGR2-F0 and NPGR2-BsR. Amplicon size around 620 bp. This primer contains de gRNA sequence. Original primer name in the protocol: DT2-R0.	AACagttatctctgcgtgagacgCAATCTCTTAGTCGACTCTAC
	NPGR2-BsR	To obtain new Crispr lines using the egg especific promoter or 35S promoter vectors from Wang et al. Genome biology 2015. Cloning using Golden Gate reaction. Reverse primer to clone a Single gRNA2 NPGR2 using pCBC-DT1T2 vector as a template. 4 primers PCR with primers NPGR2-BsF and NPGR2-F0 and NPGR2-R0. Amplicon size around 620 bp. This primer contains de gRNA sequence and the BSAI cutting site. Original primer name in the protocol: DT2-BsR.	ATTATTGGTCTCGAAACagttatctctgcgtgagacgCAA
EFOP4	EFOP4-BsFR	To obtain new Crispr lines using the egg especific promoter or 35S promoter vectors from Wang et al. Genome biology 2015. Cloning using Golden Gate reaction. Forward primer to clone a Single gRNA1 EFOP4 using pCBC-DT1T2 vector as a template. 4 primers PCR with primers EFOP4-F0 and EFOP4-R0 and EFOP4-BsR. Amplicon size around 620 bp. This primer contains de gRNA sequence and the BSAI cutting site. Original primer name in the protocol: DT1-BsF.	ATATATGGTCTCGATTGATGCACATACTCTCGCATGCGTT
	EFOP4-F0	To obtain new Crispr lines using the egg especific promoter or 35S promoter vectors from Wang et al. Genome biology 2015. Cloning using Golden Gate reaction. Forward primer to clone a Single gRNA1 EFOP4 using pCBC-DT1T2 vector as a template. 4 primers PCR with primers EFOP4-BsF and EFOP4-R0 and EFOP4-BsR. Amplicon size around 620 bp. This primer contains de gRNA sequence. Original primer name in the protocol: DT1-F0.	TGATGCACATACTCTCGCATGCGTTTTAGAGCTAGAAATAGC
	EFOP4-R0	To obtain new Crispr lines using the egg especific promoter or 35S promoter vectors from Wang et al. Genome biology 2015. Cloning using Golden Gate reaction. Reverse primer to clone a Single gRNA2 EFOP4 using pCBC-DT1T2 vector as a template. 4 primers PCR with primers EFOP4-BsF and EFOP4-F0 and EFOP4-BsR. Amplicon size around 620 bp. This primer contains de gRNA sequence. Original primer name in the protocol: DT2-R0.	AACgtcaagcgttacaagaaattCAATCTCTTAGTCGACTCTAC
	EFOP4-BsR	To obtain new Crispr lines using the egg especific promoter or 35S promoter vectors from Wang et al. Genome biology 2015. Cloning using Golden Gate reaction. Reverse primer to clone a Single gRNA2 EFOP4 using pCBC-DT1T2 vector as a template. 4 primers PCR with primers EFOP4-BsF and EFOP4-F0 and EFOP4-R0. Amplicon size around 620 bp. This primer contains de gRNA sequence and the BSAI cutting site. Original primer name in the protocol: DT2-BsR.	ATTATTGGTCTCGAAACgtcaagcgttacaagaaattCAA

Supplemental Table S7: Primers used for Cispr constructs

Gene	Locus	Mutant Name	T-DNA or CrisPr line	Primer Name	Primer Sequence (5'-3'; forward then reverse)	Additional information for genotyping
PI4Kα1	At1g49340	*pi4kα1-1*	GK502_D11	GK502_D11-LP	ACCGGTTATACCGATTTGGTC	
				GK502_D11-RP2	TTGTTTCAGCAAGATATATCG	
		pi4kα1-2	FLAG_275H12	FLAG_275H12-RP	AGTCCTACCCTCCTGAACGAG	
				FLAG_275H12-LP	TCACGACATCTTTTGTCCAAAC	
NPG1	At2g43040	*npg1-1*	SAIL_262_A01	SAIL_262_A01-LP	AGTGAGAAGGGGGAAGATGAG	
				SAIL_262_A01-RP	AGTGTTCCAACGCTCAATACG	
		npg1-2	GK_480D08	GK_480D08-LP	AAGAAGTAATGCCTGGTGTGTTTA	
				GK_480D08-RP2	TGTGTTATTGTACAAGTTCTTC	
NPGR1	At1g27460	*npgr1*	SALK_090514	SALK_090514-LP	CCCTGGAATTGCTTCCTTTAC	
				SALK_090514-RP	TTTCTCTTGCGCTCGAATTAG	
NPGR2	At4g28600	*npgr2-1*	CrisPr	NPGR2-CPR-1	CTGTTCTTTCTCGAAATATG	Sequencing
				NPGR2-CPR-2	CCTTTAGACCGTCTTCTATG	
		npgr2-2		NPGR2-CPR-1	CTGTTCTTTCTCGAAATATG	Migration on 4% agarose gel
				NPGR2-CPR-3	CCTTTAGACCGTCTTCTATG	
HYC1	At5g21050	*hyc1*	GK_767H11	GABI_767H11-LP	CTTCCTCAAGCCATGACTCAC	
				GABI_767H11-RP	ACATCATCGACTTGCAGGATC	
HYC2	At5g64090	*hyc2-2*	SALK_003807	SALK_003807-LP	TGCCCCATTAGAAACAAACTG	
				SALK_003807-RP	ACTCAACTATACATCATAACC	
		hyc2-3	SALK_040977	SALK_040977-LP	TCAGACTCAATTCCCATGGAC	
				SALK_040977-RP	GAGAGGCTCTTGCGTACAATG	
EFOP1	At5g21080	*efop1*	GK_620B11	GABI_620B11_RP	GAGGATGCTAAAAACCCCAAG	
				GABI_620B11_LP	TTCGTCGTCAAAACTTTCACC	
EFOP2	At2g41830	*efop2-1*	SALK_128017	SALK_128017_RP	TGTGCCAATTAGGACTGGAAG	
				SALK_128017_LP	TTAAAACAGGGAATGCAATCG	
		efop2-2	GK_387B07	GABI387B07ch2_LP	TTCAAGCTATGGTCCATCCAG	
				GABI387B07ch2_RP	TTCAAACATAGCTCTCCCTCG	
EFOP3	At1g05960	*efop3-1*	SALK_121262	SALK_121262_RP	TTCTGGGTTTTCAGGGTTCTC	
				SALK_121262_LP	TCGACAAGAGTATTGCAACCC	
		efop3-2	SALK133976	SALK_133976_LP	AGGGCCAAGAAGATACCAAAG	
				SALK_133976_RP	CATGGGACTCATAACCACTGG	
EFOP4	At5g26850	*efop4-2*	CrisPr	EFOP4-CPR-1	GTATAGATGAGGTCTGGTTTC	Msl1 digestion and migration on 4% agarose gel
				EFOP4-CPR-2	GCAGGACAGCAAATGCACATACaCTCGCAT	
		efop4-4		EFOP4-CPR-1	GTATAGATGAGGTCTGGTTTC	Migration on 2% agarose gel
				EFOP4-CPR-3	TTAGGGATACGGATTGGGTTC	

Supplemental Table S8: Genotyping primers

Name	Sequence ordered
microRNA 2	ACAAGTTTGTACAAAAAAGCAGGCTCaaacacacgctcggacgcatattacacatgttcatacacttaatactcgctgttttgaattgatgttttaggaatatatatgt**aga**TAATAGGTTGTGCCCCAACGT**tcacaggtcgtgatatgattcaattagcttccgactcattcatccaaataccgagtcgccaaaattcaaactagactcgttaaatgaatgaatgatgcggtagacaaattggatcattgattctctttga**ACGTTGGGGCACAACCTATTA**tct**ctcttttgtattccaattttcttgattaatctttcctgcacaaaaacatgcttgatccactaagtgacatatatgctgccttcgtatatatagttctggtaaaattaacattttgggtttatctttatttaaggcatcgccatgACCCAGCTTTCTTGTACAAAGTGGT
microRNA 3	ACAAGTTTGTACAAAAAAGCAGGCTCaaacacacgctcggacgcatattacacatgttcatacacttaatactcgctgttttgaattgatgttttaggaatatatatgt**aga**TAAAAGTAATGCAAGCGTCGC**tcacaggtcgtgatatgattcaattagcttccgactcattcatccaaataccgagtcgccaaaattcaaactagactcgttaaatgaatgaatgatgcggtagacaaattggatcattgattctctttga**GCGACGCTTGCATTACTTTTA**tct**ctcttttgtattccaattttcttgattaatctttcctgcacaaaaacatgcttgatccactaagtgacatatatgctgccttcgtatatatagttctggtaaaattaacattttgggtttatctttatttaaggcatcgccatgACCCAGCTTTCTTGTACAAAGTGGT

in red: AttB1 and AttB; in black: artificial microRNA; in blue, the stuctural part of the microRNA with the hairpin sequence in [illegible]

Supplemental Table S9: microRNA sequences

Percentage of pollen

Test	Pair	p-value	χ^2	df
Chi-square	Col0 / *pi4kα1-1*	< 2.2e-16	533.26	1
	Col0 / *pi4kα1-2*	< 2.2e-16	504.91	1
	pi4kα1-1 / *pi4kα1-2*	0.3129	1.0184	1
	pi4kα1-1 / *pi4kα1-1 PI4Kα1prom::PI4Kα1-3'UTR*	< 2.2e-16	205.05	1
	Col0 / *pi4kα1-1 PI4Kα1prom::PI4Kα1-3'UTR*	1.633e-11	45.368	1
	Col0 / *npg1-2*	0.001401	13.141	2
	Col0 / *npg1-2 npgr1*	< 2.2e-16	120.72	2
	Col0 / *npg1-2 npgr1 npgr2-2*	< 2.2e-16	374.82	2
	Col0 / *hyc1*	< 2.2e-16	414.76	2
	Col0 / *efop3-1 efop4-2*	< 2.2e-16	368.03	2
	Col0 / *efop3-1 efop4-4*	< 2.2e-16	276.05	2
	npg1-2 npgr1 / *npg1-2 npgr1 npgr2-2*	< 2.2e-16	127.72	2
	efop3-1 efop4-2 / *efop3-1 efop4-4*	0.2919	2.4625	2

Percentage of seeds

Test	Pair	p-value	χ^2	df
Chi-square	Col0 / *hyc2-2*	< 2.2e-16	72.092	1
	Col0 / *hyc2-3*	< 2.2e-16	100.43	1
	Col0 / *hyc2-2 HYC2prom::HYC2-mCITRINE*	0.9901	0.00015379	1
	Col0 / *hyc2-2 HYC2prom::HYC2-2xmCHERRY*	0.3797	0.77178	1
	hyc2-2 / *hyc2-2 HYC2prom::HYC2-mCITRINE*	< 2.2e-16	77.067	1
	hyc2-2 / *hyc2-2 HYC2prom::HYC2-2xmCHERRY*	9.994e-16	64.432	1

microRNA root length

Test	Pair	p-value
Wilcoxon	Col0 DMSO / Col0 Estradiol #3	0.0005085
	microRNA2 DMSO / microRNA2 estradiol #3	1.008e-08
	Col0 DMSO / Col0 Estradiol #6	0.0009169
	microRNA2 DMSO / microRNA2 estradiol #6	1.503e-08

Cytosol/PM ratio

Test	Pair	p-value
Wilcoxon	1xPH^{FAPP1} DMSO / estradiol	0.001118
	1xPH^{FAPP1} E50A DMSO / estradiol	0.2996
	P4M DMSO / estradiol	0.09646

Supplemental Table S10: Statistical analysis

Supplemental references

Elsayad, K., Werner, S., Gallemi, M., Kong, J., Sanchez Guajardo, E.R., Zhang, L., Jaillais, Y., Greb, T., and Belkhadir, Y. (2016). Mapping the subcellular mechanical properties of live cells in tissues with fluorescence emission-Brillouin imaging. Science signaling **9,** rs5.

Jaillais, Y., Hothorn, M., Belkhadir, Y., Dabi, T., Nimchuk, Z.L., Meyerowitz, E.M., and Chory, J. (2011). Tyrosine phosphorylation controls brassinosteroid receptor activation by triggering membrane release of its kinase inhibitor. Genes & development **25,** 232-237.

Karimi, M., Inze, D., and Depicker, A. (2002). GATEWAY vectors for Agrobacterium-mediated plant transformation. Trends in plant science **7,** 193-195.

Karimi, M., Bleys, A., Vanderhaeghen, R., and Hilson, P. (2007). Building blocks for plant gene assembly. Plant physiology **145,** 1183-1191.

Marques-Bueno, M.M., Morao, A.K., Cayrel, A., Platre, M.P., Barberon, M., Caillieux, E., Colot, V., Jaillais, Y., Roudier, F., and Vert, G. (2016). A versatile Multisite Gateway-compatible promoter and transgenic line collection for cell type-specific functional genomics in Arabidopsis. The Plant journal : for cell and molecular biology **85,** 320-333.

Platre, M.P., Noack, L.C., Doumane, M., Bayle, V., Simon, M.L.A., Maneta-Peyret, L., Fouillen, L., Stanislas, T., Armengot, L., Pejchar, P., Caillaud, M.C., Potocky, M., Copic, A., Moreau, P., and Jaillais, Y. (2018). A Combinatorial Lipid Code Shapes the Electrostatic Landscape of Plant Endomembranes. Developmental cell **45,** 465-480 e411.

Siligato, R., Wang, X., Yadav, S.R., Lehesranta, S., Ma, G., Ursache, R., Sevilem, I., Zhang, J., Gorte, M., Prasad, K., Wrzaczek, M., Heidstra, R., Murphy, A., Scheres, B., and Mahonen, A.P. (2016). MultiSite Gateway-Compatible Cell Type-Specific Gene-Inducible System for Plants. Plant physiology **170,** 627-641.

Simon, M.L., Platre, M.P., Marques-Bueno, M.M., Armengot, L., Stanislas, T., Bayle, V., Caillaud, M.C., and Jaillais, Y. (2016). A PtdIns(4)P-driven electrostatic field controls cell membrane identity and signalling in plants. Nat Plants **2,** 16089.

Simon, M.L., Platre, M.P., Assil, S., van Wijk, R., Chen, W.Y., Chory, J., Dreux, M., Munnik, T., and Jaillais, Y. (2014). A multi-colour/multi-affinity marker set to visualize phosphoinositide dynamics in Arabidopsis. The Plant journal : for cell and molecular biology **77,** 322-337.

Stevenson, J.M., Perera, I.Y., and Boss, W.F. (1998). A phosphatidylinositol 4-kinase pleckstrin homology domain that binds phosphatidylinositol 4-monophosphate. The Journal of biological chemistry **273,** 22761-22767.

Stevenson-Paulik, J., Love, J., and Boss, W.F. (2003). Differential regulation of two Arabidopsis type III phosphatidylinositol 4-kinase isoforms. A regulatory role for the pleckstrin homology domain. Plant physiology **132,** 1053-1064.

Xue, H.W., Pical, C., Brearley, C., Elge, S., and Muller-Rober, B. (1999). A plant 126-kDa phosphatidylinositol 4-kinase with a novel repeat structure. Cloning and functional expression in baculovirus-infected insect cells. The Journal of biological chemistry **274,** 5738-5745.
