# Supplementary material for: A nanodomain-anchored scaffolding complex is required for the function and localization of phosphatidylinositol 4-kinase alpha in plants
Source: Plant Cell. 2021 May 19;34(1):302–32. doi: 10.1093/plcell/koab135 (PMC8774046; doi:10.1093/plcell/koab135)
Supplement: koab135_Supplementary_Data [file koab135_supplementary_data.zip › tpc.01032.2020-s02.pdf]

THE PLANT CELL

Author Checklist – *post-acceptance* **Ms #01032**

The following items are required prior to final acceptance. Acceptance may be delayed if these elements are not clearly presented or are found to be out of compliance with journal standards. Please review this form carefully. **If a box is checked, the item needs your attention!**

ORCID REQUIREMENT

☒ The journal requires all authors of accepted manuscripts to provide an ORCID digital identifier. An ORCID unequivocally distinguishes you from every other researcher and ensures that your work is recognized. See https://orcid.org/ for more details and to register. Registration takes only 30 seconds. Authors who do not have an ORCID associated with their journal account will receive an email with a link and must complete this process before the manuscript can be published. Journal staff are unable to make changes to authors' individual accounts.

TAIR

☒ The Arabidopsis Information Resource (TAIR) collects functional annotation data about Arabidopsis genes. This includes information about a gene's molecular function (e.g., kinase activity, ATP synthetase activity), the biological process/es it is involved in (e.g., endosperm development, threonine biosynthesis), its subcellular location (e.g., nucleus, ER), anatomical or developmental expression pattern (e.g., leaf, ovule, flower stage 10, seedling stage), or its partner in a protein-protein interaction (e.g., AT1G01010 interacts with AT1G01020). If your paper contains results falling into one or more of these categories for Arabidopsis genes, we request that you submit these to TAIR by filling in the form provided at the following URL: http://www.arabidopsis.org/doc/submit/functional_annotation/123. For more information, please contact curator@arabidopsis.org.

MATERIALS DISTRIBUTION

☐ Manuscript needs a materials distribution statement. All manuscripts must include the following statement as an unnumbered footnote: "The author(s) responsible for distribution of materials integral to the findings presented in this article in accordance with the policy described in the Instructions for Authors (www.plantcell.org) is (are): John D. Author (author@college.edu)."

AUTHOR CONTRIBUTIONS

☐ Each article must include an Author Contributions section (after Acknowledgments) to detail how each author contributed to the research or writing of the manuscript. Note which of the following tasks each author performed: designed the research; performed research; contributed new analytic/computational/etc. tools; analyzed data; or wrote the paper. Optional: include which author(s) contributed to each figure, table, or data set in this section.

DATA AVAILABILITY

☒ Accession numbers must be provided for all genes reported and other major genes discussed. All large-scale data (e.g., genome sequences, annotations, genetic maps, transcript profiles, other sequencing data, proteomic data sets, metabolic profiles) that are integral to the manuscript must be submitted to a permanent public repository with open access and must be made publicly available immediately upon publication. Accession codes, unique identifiers, or web links for publicly available data sets must be provided in an Accession numbers section at the end of the Methods.

NOMENCLATURE

☒ Nomenclature must conform to accepted community standards for the species studied. Arabidopsis nomenclature rules should not be used for certain other species (including maize and rice) and vice versa. Please see the Instructions for Authors for details and links.

☐ All gene and protein symbols used must have priority in the literature. New gene symbols must be approved by the editors and should be compliant with the naming conventions of the relevant research community. New Arabidopsis gene names should be registered with TAIR.

A name change can be proposed, but authors should first consider names that have priority to avoid creating confusion in the literature. If a gene name is being changed, the original genomic name should 1) appear in the manuscript title, 2) appear prominently together with the new name in several places in the beginning of the manuscript, and 3) appear first wherever the two names appear together.

THE PLANT CELL

DATA NOT SHOWN

☐ Citation of "data not shown" or "unpublished results" is strongly discouraged, and is not permitted for important conclusions; critical data must be available, or should not be cited.

☐ Citation of "personal communication", where permitted, must be accompanied by written permission from all persons cited who are not co-authors on the manuscript. Permissions should be emailed to Production Manager Susan Entwistle at susan@aspb.org.

METHODS REPORTING

☒ Plant growth conditions (soil, amendments, media, cell culture, etc.) must be adequately described, including details of light quality—spectrum or bulb type—as well as intensity.

☐ PCR primers (RT-qPCR, cloning, etc.): all PCR primers must be provided in the Methods section or a supplemental table.

☐ Cloning information: cloning and DNA constructs must be fully described (use a supplemental table if necessary).

☒ Antibodies: provide the source (and dilutions used) for all commercial antibodies, including catalog/lot #, where applicable, or a complete description of non-commercial antibodies in the Methods.

☐ Statistical analyses, the nature of replicates, and error bars must be adequately described in the Methods and/or figure legends. *Note that "biological replicate" alone is insufficient; this must be precisely defined for your experiments.*

☐ Please provide ANOVA and/or T-test results in supplemental tables or a supplemental data set (i.e., showing variables, parameters, degrees of freedom, and test statistics).

☐ Phylogenetic analysis: please ensure that phylogenetic analysis meets these criteria:

Methods used for sequence analysis must be reported in full with citations and software and parameter values (even if only default values were used) in a separate section of Methods entitled "Phylogenetic Analysis". Please note that CLUSTAL does not produce an acceptable phylogeny; use a true phylogenetic analysis program (e.g., MEGA, RAxML, IQ-TREE, RevBayes, BEAST). Alignments used to produce phylogenies should be produced with an appropriate alignment program (e.g., MAFFT, T-Coffee). Statistical support for nodes in any phylogenetic tree figures must be reported (i.e., posterior probabilities or bootstrap values with MCMC search sample or replicate numbers reported in text), and tree branch lengths (e.g. time, substitutions per site, coalescence units) properly described in tree figure captions. If phylogenetic trees are depicted or interpreted as rooted, the criterion used for rooting (e.g., midpoint, outgroup) must be provided, and if outgroup rooting is used, the basis for the choice of outgroup should be explained. Sequence alignments (e.g. FASTA, PHYLIP, Nexus format) and machine-readable tree files (e.g. Newick, Nexus, NeXML format) must be deposited in a persistent database (e.g. Dryad, TreeBASE) or provided as Supplemental Files.

☐ Bimolecular fluorescence complementation (BiFC): please ensure that BiFC assays meet these criteria:

Expressing unfused YFP fragments is not a sufficient control for BiFC experiments (see the Commentary from Bock and Kudla https://doi.org/10.1105/tpc.16.00043). Ideally, negative controls should include a mutated version of one of the interacting proteins carrying a defect in the interaction domain or a related non-interacting protein from the same protein family. If neither a mutated protein version nor a suitable closely related protein are available as negative controls, an unrelated protein (but, ideally, structurally similar and expressed in same subcellular compartment) can be used. It is essential that exactly the same orientations are used for negative controls as for the positive interaction. It is also desirable to show results as quantitative data (for example, from 10 randomly chosen regions of interest of infiltrated leaves) and not merely one or two "representative images" (many papers show only a single image). Any representative image should display a complete cell, with the nucleus and the center of the cell in the focal plane.

☐ Electrophoretic Mobility Shift Assay (EMSA): please ensure that EMSA experiments meet these criteria:

EMSA is used to show binding of proteins to nucleic acids. Often, it is of interest to know if the binding of the protein shows sequence specificity for the nucleic acid. Competition experiments may be used to demonstrate sequence-specific binding, by adding e.g. 50-100 molar excess of unlabeled competitor DNA to the reaction mix with the labeled probe DNA. Unlabeled wild-type DNA is often used as a positive control in these experiments. However, it is not meaningful to show competition with unlabeled wild-type DNA *unless the competition with a mutated or scrambled site is included*. Competition with wild-type alone (without the competition using mutated or scrambled sites), merely indicates that binding is not specific for the label (e.g. 32P or biotin). If your experiments show only unlabeled wild-type as positive controls, please ensure that you make no claims about "sequence specificity" of the binding, and do not show results of competition with several concentrations of excess in this case, as it is not meaningful. See the Instructions for Authors for more information on appropriate controls.

DATA PRESENTATION

Tables

☐ Tables in the main manuscript or supplemental materials must be no more than 2 pages in length; any table longer than 2 pages should be provided in Excel format, labeled as a “Supplemental Data Set”.

☐ Tables in the main manuscript must be included in the manuscript file (Word document) after the Methods section, using the "insert table" feature in Word (do not insert images or colors, shading, etc. into tables in the manuscript file).

Figures

Figures should be prepared as professionally as possible and should have a consistent appearance (i.e. as if made by the same person).

☐ All text and elements of each figure should be clearly visible at printed size. Avoid tiny text and a lot of white space.

☐ Color in figures should be used consistently, as much as possible, across multiple figures (i.e. the same colors used for the same genotypes/treatments across multiple figures). Avoid overuse of hatching and patterns.

☒ Many readers of the Journal (1 in 12, on average) have some form of color-deficient vision. To ensure that all readers will be able to comprehend your data, please do not use the combination of red and green; use magenta and green instead.

☒ Make consistent use of the same, sans-serif font (e.g. Arial) for all text elements in all figures. Use the same (or similar) size font for panel letters (A, B, C, etc.) in all figures.

☐ Bars in bar graphs should be the same width (or close) for multiple bar graphs in different panels of a single figure (*this means spaces between bars may be different, but bars should be the same width*).

☒ Use proper symbols for +/− (instead of a dash or hyphen **-** for minus).

☐ Numerical values on all graph axes should have consistent number of significant digits (e.g. 5.0, 10.0, 15.0 **or** 5, 10, 15 etc.); however, the origin should be "0", not "0.0".

☐ Images made from screen shots should be adjusted to high resolution where necessary and illegible or overlapping text or other elements omitted or re-drawn using a suitable graphics program to be legible at printed size.

☐ Figure legends must be clear and adequately describe the data shown. Replicates must be defined precisely: the term "biological replicate" should be defined explicitly in the context of each experiment in the legend (or Methods). All elements of figures must be defined accurately, e.g., the axes, abbreviations, symbols, how values were obtained, scale bars, and error bars.

Figure Files

☒ For final production, two sets of **main manuscript** figure files are needed:

1) Provide main manuscript figures as **high resolution** image files, one file for each figure, **without legends**. A high resolution or vectorized pdf file is the best file type. Tiff, eps, ai, or pdf files may be submitted (Word, ppt, or jpg files are not recommended). There is no limit to file size.

2) For advance publication of the raw pdf, please provide an additional file of all figures combined, **with legends.** To create this file, save a copy of each figure without the legend as a jpg at 300 dpi. Then, using e.g. PowerPoint or Word, insert one jpg figure per page in portrait format, size figures to fit each one on a single page (8.5x11 or A4 page size), and add the legend underneath, *on the same page as the figure*, using single-spaced Arial 9 or 10 pt font. Save and submit the “combined figures” file in PDF format.

Each figure together with its legend should be on a single page in 8.5x11 or A4 size in portrait format. Reduce font size of the legends (use Arial 9 or 10 pt size) and make single-spaced legends that are full page width to make the best use of space. The legend may be on a second page *if absolutely necessary*. **Be sure to merge layers and/or flatten the image before saving copies of image files**; if layers are not merged this can greatly increase file size. *Please minimize the size of this file (aim for <20 MB), while maintaining image resolution as much as possible; check that all elements of figures are clear and legible; this will be the advance publication version of your figures.*

Authors using Adobe Illustrator can add legends in Illustrator and save a copy of the file as a pdf; be sure to adjust page sizes to 8.5X11 or A4 and **uncheck** "preserve illustrator editing capabilities", "create layers", and "embed thumbnail images" to minimize file size.

☒ TOC Icon. A graphic icon is used to represent each paper in the Table of Contents (TOC). **The icon must be exactly 96x96 pixels**, in gif, tif, jpg, or pdf format, and should illustrate a concept of the paper (we cannot accept animated gif files). Select an appropriate figure (in the manuscript or not) and reduce it to its strongest, most important graphic elements, removing unessential text. **When you upload the file, please choose "TOC Icon" as the file type.** Let the science editor know if you need assistance creating or submitting the icon.

Supplemental Data

☐ Multiple supplemental figures and tables should be combined and submitted as a single .pdf file named “Supplemental Data”, no larger than 50 MB. Files to be merged into the single supplemental data file include supplemental figures, followed by supplemental tables. Supplemental tables in this file are restricted to 1-2 pages in length.

☐ Any supplemental table longer than 2 pages must be labeled as a “Supplemental Data Set” and provided in Excel format.

☐ Supplemental data sets should include complete legends (on a separate sheet in the Excel file if necessary).

☐ Please adjust numerical values in data sets for significant figures (do not simply use default Excel settings), and make use of freeze panes and shading to help the reader navigate large files. If convenient, multiple Supplemental Data Sets may be submitted on separate sheets in a single Excel file.

☐ Each supplemental figure must provide direct support for a figure in the main manuscript, and a supporting statement should be included in each supplemental figure legend, immediately after the title e.g. “(Supports Figure 1).”

☒ Arial font (or similar sans serif font, e.g. Helvetica) should be used in all Supplemental Figures, Supplemental Tables, and Supplemental Data Sets and their corresponding legends (please use single spacing). The only exception is for nucleotide sequences (primers, etc.) where a mono-spaced font such as Courier should be used.

☐ All supplemental movie legend(s) should be included in a single Word or PDF document.

☒ Add the following header using Arial 10 pt font to each page of your Supplemental Data pdf and all other supplemental files: Supplemental Data. Author et al. (2020). Plant Cell.

☒ File size: each supplemental file ideally should be less than 10 MB and is limited to 50 MB maximum size.

OPTIONAL ITEMS

Figure/Table/Data Set Attributions. We encourage identifying the author(s) responsible for creating each figure, table, or data set, to give proper attribution to authors for their individual contributions to a manuscript. If desired, please either include the information in the author contributions section, or add a statement to each legend, e.g. “J.D., A.S., and B.W. contributed the data and created the figures for panels A, B, and C, respectively”, or “J.D., A.S., and B.W. contributed the data for panels A, B, and C, respectively, and J.D. created the figure.”

Nutshell Summary. To help promote your work to a wide audience, you may choose to publish a plain-language "In a Nutshell" summary of your work. The Nutshell (text only) will be published in the journal with the final version of your paper, *provided the form is returned before the science editor issues final acceptance on your manuscript*. In addition, the Nutshell will be published in blog form with a title and image at https://plantae.org/research/the-plant-cell/#in-a-nutshell. A form for completing the Nutshell was sent to you on provisional acceptance of your manuscript. If you have not already emailed your completed Nutshell form to the science editor, you may upload it to the system with your final files: upload as “Supplemental Material” with the file name “Nutshell”.

Author Profile(s). We recognize the hard work that goes into being first author of a Plant Cell paper by publishing a “First Author Profile” for each first author alongside your paper. If the first author(s) would like to submit a profile, please send an email to Suzanne Cholwek (scholwek@aspb.org) with “First Author Profile” in the subject line and an attached Word document for each first author that includes a jpg photo and the following information:

Name:
Current Position:
Education:
Non-scientific Interests:
Brief bio:

Note: if you would like your profile posted to The Plant Cell WeChat account, please include a Chinese translation below the English version.

THE PLANT CELL

MANUSCRIPT FORMAT

Title and abstract. The title and abstract are critical for engaging readers and enticing them to read the paper. The title should be concise: use active voice, spell out genes names (or otherwise define), avoid acronyms. The species or organism under study does not need to be in the title, but this information should be in the abstract (i.e. brief description of experimental system(s) used). See: http://genestogenomes.org/how-to-write-titles-that-tempt/.

The abstract should be no more than ~200 words. Include only major results and implications and put general background and methods in the text.

One-sentence summary. Please include a one-sentence summary of the work on the title page that provides a non-technical summary of the broad significance of the research findings in plain language (for a general audience) that does not merely repeat what is already in the abstract. The use of complex scientific terms and acronyms should be avoided.

References: Upon final submission, in-text citations should be formatted using an author-date format; ***not numbered***, with references **in alphabetical order** in the Reference list. Any author-date style is acceptable.

Species names: Please note that organisms should be referred to by their common name at first use (if a standard common name applies), and the Latin name should be given in parentheses. Subsequent references to organisms can be either Latin or common names but should be consistent throughout the manuscript.

Title page: Please follow the format shown on the next page. Use single spacing of abstract and other information if necessary to fit the title, author info, and abstract on a single page as shown. Use line numbers on subsequent pages; not the title page.

Sample title page

LARGE-SCALE BIOLOGY ARTICLE [or e.g. RESEARCH ARTICLE, BREAKTHROUGH REPORT]

Spatial transcriptional signatures define margin morphogenesis along the proximal-distal and medio-lateral axes in tomato (*Solanum lycopersicum*) leaves

Ciera C. Martinez[1,2,3], Siyu Li[3], Margaret R. Woodhouse[3], Keiko Sugimoto[4], and Neelima R. Sinha[3*]

[1]Department of Molecular and Cellular Biology, University of California at Berkeley, Berkeley, CA 94709, USA

[2]Berkeley Institute of Data Science, University of California at Berkeley, Berkeley, CA 94709, USA

[3]Department of Plant Biology, University of California at Davis, Davis, CA 95616, USA

[4]RIKEN Center for Sustainable Resource Science, Tsurumi, Yokohama, 15 230-0045 Japan

*Author for correspondence nrsinha@ucdavis.edu

Short title: Transcriptional signatures during leaf development

One-sentence summary: Rigorous structural characterization, laser capture microdissection, and transcriptomic sequencing reveal how gene expression patterns regulate early morphogenesis of the compound tomato leaf.

The author responsible for distribution of materials integral to the findings presented in this article in accordance with the policy described in the Instructions for Authors (www.plantcell.org) is Ciera C. Martinez (ccmartinez@berkeley.edu).

ABSTRACT

Leaf morphogenesis involves cell division, expansion, and differentiation in the developing leaf, which take place at different rates and at different positions along the medio-lateral and proximal-distal leaf axes. The gene expression changes that control cell fate along these axes remain elusive due to difficulties in precisely isolating tissues. Here, we combined rigorous early leaf characterization, laser capture microdissection, and transcriptomic sequencing to ask how gene expression patterns regulate early leaf morphogenesis in wild-type tomato (*Solanum lycopersicum*) and the leaf morphogenesis mutant *trifoliate*. We observed transcriptional regulation of cell differentiation along the proximal-distal axis and identified molecular signatures delineating the classically defined marginal meristem/blastozone region during early leaf development. We describe the role of endoreduplication during leaf development, when and where leaf cells first achieve photosynthetic competency, and the regulation of auxin transport and signaling along the leaf axes. Knockout mutants of *BLADE-ON-PETIOLE2* exhibited ectopic shoot apical meristem formation on leaves, highlighting the role of this gene in regulating margin tissue identity. We mapped gene expression signatures in specific leaf domains and evaluated the role of each domain in conferring indeterminacy and permitting blade outgrowth. Finally, we generated a global gene expression atlas of the early developing compound leaf.
