# Supplementary material for: A nanodomain-anchored scaffolding complex is required for the function and localization of phosphatidylinositol 4-kinase alpha in plants
Source: Plant Cell. 2021 May 19;34(1):302–32. doi: 10.1093/plcell/koab135 (PMC8774046; doi:10.1093/plcell/koab135)
Supplement: koab135_Supplementary_Data [file koab135_supplementary_data.zip › tpc.01032.2020-s03.pdf]

A nanodomain-anchored scaffolding complex is required for the function and localization of the phosphatidylinositol 4-kinase alpha in plants

Noack LC, Bayle V, Armengot L, Rozier F, Mamode-Cassim A, Stevens FD, Caillaud MC[1], Munnik T, Mongrand S, Pleskot R, Jaillais Y

Corresponding author: Jaillais Y (yvon.jaillais@ens-lyon.fr)

Review timeline:

**TPC2020-RA-01032**	Submission received:	December 10, 2020
	1st Decision:	January 11, 2021 *revision requested*
**TPC2020-RA-01032R1**	1st Revision received:	April 14, 2021
	2nd Decision:	April 26, 2021 *acceptance pending, sent to science editor*
	Final acceptance:	May 10, 2021

REPORT: (The report shows the major requests for revision and author responses. Minor comments for revision and miscellaneous correspondence are not included. The original format may not be reflected in this compilation, but the reviewer comments and author responses are not edited, except to correct minor typographical or spelling errors that could be a source of ambiguity.)

TPC2020-RA-01032 1st Editorial decision – *revision requested* **January 11, 2021**

We have received reviews of your manuscript entitled "A nanodomain anchored-scaffolding complex is required for PI4Kα function and localization in plants." Thank you for submitting your best work to The Plant Cell. The editorial board agrees that the work you describe is substantive, falls within the scope of the journal, and may become acceptable for publication, pending revision and potential re-review.

We ask you to pay attention to the following points in preparing your revision:

Both reviewers were quite positive about the results presented in this manuscript, although Reviewer 2 raised a number of important issues. Please pay particular attention to the following points.

1) It is important to address the questions raised by Reviewer 1, in particular to assess the relative impact of loss of PI4Ka1 on PI4P accumulation, and to address the confusion regarding the relative functionality of PI4Ka1fusion proteins.

2) Reviewer 2 raises an issue that likely is important to the interpretation of the data presented in the manuscript. Namely, whether the tetrameric complex of PI4Ka1, NPGR2, EFOP2, and HYC is stable or not. This is particularly important to interpretation of some of the double, and triple mutant phenotypes described.

3) Please ensure that you respond to all comments from the reviewers.

We should stress that we are reluctant to see manuscripts undergoing multiple rounds of revision and would be unlikely to offer you more than one chance to satisfy the reviewers. --- Reviewer comments:

TPC2020-RA-01032R1 1st Revision received **April 14, 2021**

Reviewer comments and **author responses**:

Reviewer #1:

In the current manuscript, Jaillais and co-workers have extensively characterised the role of PI4Ka1 during Arabidopsis development and have identified that it forms a functional (active) complex with NPH/HYC/EFOP

proteins. This is an important study, since it has been reported for quite some time that that PI4Ka1 is essential and important for PI4P production at the plasma membrane, but never really shown in a convincing manner. The manuscript is well-written and the authors have performed an impressive amount of experiments that leave little room for criticism. They have performed all the proper controls and analysed the data to the highest standards. I have only two minor comments that I would like to see addressed by the authors:. Paragraphs single spaced with 6 pt before and after. Put 12 pt spacing before and after the revision headings (sections with top and bottom borders).

Point 1. Do the authors have an estimation or maybe data that could tell us how much PI4Ka1 is contributing to the total PI4P pool? Did they attempt to use their biosensor in some of the mutants of the PI4Ka1 interactors that still made embryo's or dwarfed plants? It is not essential to provide this data, but I think it would be a nice extra piece of information.

RESPONSE: This is a very valid point that we have tried to address extensively through many different approaches. However, this has proven to be very tricky to address, mainly because every time we fully knock out the family of one of the subunit of the PI4Kα1-complex we observed very strong lethality early during development (either on pollen or embryo). We tried to localize our PI4P sensors in shriveled pollens of the pi4kα1 mutant, but they are very autofluorescent preventing meaningful interpretations. We also crossed our PI4P sensors in the hyc2 mutant, which is embryo lethal. But the dissection procedure led to a very variable localization pattern, which added to the fact that only 25% of the embryos are mutants in hyc2+/- siliques, made it difficult to conclude.

Aiming to address this point, we have now raised inducible artificial microRNA lines against PI4Kα1 (new Figure 6). We do detect an impact on the localization of the PH-FAPP1 PI4P sensor at the plasma membrane following PI4Kα1 knockdown. However, this is a rather weak effect, likely due to the remaining PI4Kα1 expression (as detected in western blot experiments). This new set of experiments confirmed a role for PI4Kα1 in the production of PI4P at the plasma membrane. However, using a knockdown strategy, we cannot conclude about the relative contribution of PI4Kα1 in the total PI4P production. Yet, we can speculate about this point. Indeed, we know that 1) the pi4kβpi4k1β2 double mutant, in which both kinases are fully knocked-out, has no detectable diminution of total PI4P (Lee et al., 2019 EMBO Journal), and 2) the pool of PI4P at the plasma membrane is quantitatively much more abundant than in the TGN (Simon et al., 2016 Nature plants). Together, we can thus hypothesize that PI4Kα1 is likely responsible for most of the PI4P production in plant cells. The fact that PI4Kα1, but not PI4Kβs, is absolutely essential for cell survival argues in favor of this model, which nonetheless remains to be experimentally validated. We are now busy building inducible and tissue-specific knock-out of the PI4Kα1 complex using the CRISPR-cas9 technology, which we hope will enable us to address this point more directly. However, this is a long-term experiment that we believe extend beyond the scope of this manuscript. We are now discussing this point in the new version of the manuscript (line 908 to 919 in the manuscript with highlighted changes)..

Point 2. I am confused about the passage 661-675. The authors first state that fusions to PI4Ka1 are not functional. Nonetheless, the PI4Ka1::PI4Ka1:mCitrine-Lti6b fusion complements the npg1-2 mutant. Does this mean that pollen does not require an active PI4Ka1 at the plasma membrane or that the chimeric PI4Ka1 could still recruit/interact with native PI4ka1 proteins? Please explain this further.

RESPONSE: We have clarified this point by performing an additional complementation experiment and asking whether the PI4Ka1::PI4Ka1:mCitrine-Lti6b construct can rescue the pi4kα1 mutant. This is now discussed lines 806 to 825 of the revised manuscript: "As discussed above, tagged version of PI4Kα1, including PI4Kα1-mCITRINE, were not functional as they did not complement the pi4kα1 mutant (Table S3). Similarly, we did not retrieve any complemented pi4kα1 mutant line expressing PI4Kα1prom:PI4Kα1-mCITRINE-Lti6b (Table S3). This raised the question on how the non-functional PI4Kα1-mCITRINE-Lti6b chimeric construct was able to rescue the npg1-2 mutant. One possibility is that PI4Kα1 naturally forms homodimer. In that scenario, the non-functional PI4Kα1-mCITRINE-Lti6b would recruit the endogenous –and functional– PI4Kα1 at the plasma membrane in the absence of npg1, and thus complement the npg1 mutant but not the pi4kα1 mutant. We currently do not know whether PI4Kα1 is able to dimerize. However, structural data from the animal field showed that the PI4KαIII complex dimerizes at the plasma membrane (Lees et al., 2017b), suggesting that it could also be the case in plants."

Reviewer #2:

In the presented manuscript, the authors identify novel interaction partners of the PI4-Kinase isoform PI4K1, which form a tetramer complex essential for PI4K1 subcellular localization at the plasma membrane. Besides PI4K1 itself,

the complex contains proteins from the NO-POLLEN-GERMINATION (NPG), EFR3-OF-PLANTS (EFOP), and HYCCIN (HYC) families. According to the authors, the main function of NPG's is to bridge PI4K and HYC with EFOP, a protein whose acetylation is essential for PI4K plasma membrane localization. While PI4P diffuses laterally at the PM, PI4K complex localizes in stables nanodomains at the PM. Yet, none of the complex members seems to be required for the establishment of these nanodomains. Knockout of pi4kα1 results in severe pollen abnormality and male sterility, a phenotype that can be observed to a similar extent in different mutant alleles and multiple mutants of the PI4Kα1 complex partners. Implementing a broad range of techniques, reaching from proteomic to genetic approaches as well as various imaging methods, Noack et al. provide novel insights into the mechanism underlying PI4Kα1 subcellular localization and proof the biological relevance of this process. Altogether, the submitted data give a complex yet cohesive picture of the novel mechanism and the implemented experiments build nicely on one another, contributing to an overall convincing outline. However, some statements seem far-fetched and additional biochemical/genetic evidence is needed to support these claims.

Point 1. The authors convincingly show by several methods that PI4Kα1 interacts with NPGR2. The interaction of PI4K and HYC2 and EFOP2 seems to be less clear based on the weaker bands observed in Figure 1. While the interaction between HYC family members with the NPG interactors is clear, biochemical evidence of the tetrameric complex formation of HYC-PI4K-NPG-EFOP is missing. Purification of the whole complex in native conditions should be performed to claim that four proteins act together in a scaffolding complex. The authors have several transgenic lines harboring EFOP2, NPGR2 and HYC proteins tagged with different epitopes as well as an antibody against PI4K, therefore the detection of the complex by chromatography purification should be doable.

RESPONSE: We extensively tried to purify the complex in native conditions using blue native PAGE experiments. However, we were not able to find adequate detergents to solubilize the complex. Findings the right conditions for native purification of multi-subunit complexes is very empirical and may not be straightforward. We thus opted for an alternative strategy. Because the complex identified in our study is likely functionally and structurally equivalent to the PI4Kα1 complex found in metazoan, whose structure is known (see Lees et al., 2017 PNAS), we reasoned that we could use structural modeling to address the overall structural conservation of 1) individual subunits, 2) their specific binding interface and 3) the overall structure of the complex. This approach confirmed that PI4Kα1/HYC1/NPG1 very likely form a stable trimeric complex. Briefly, we modeled individual subunit based on the resolved structure of their human counterpart (Figure S2). We then predicted their respective binding interfaces using protein-protein docking algorithms (Figure 2). Using this approach, we found 1) that the predicted binding interface closely matched those found in metazoans, 2) that these interfaces are made of highly conserved residues and are thus likely structurally/functionally relevant and 3) when put together through this approach, the overall structure of the predicted Arabidopsis complex matches almost perfectly the structure of the PI4KIIIa1/TTC7/FAM126 trimer in human. This analysis is now presented in a new Figure 2 (and sup Figure 2) and further support that these three proteins act together in a complex. In addition, the existence of the complex in plants is not only supported by biochemical data (including coIP and yeast two hybrids), but also by in depth genetic analyses (i.e. similar pollen phenotype upon depletion of each subunit), colocalization (at the plasma membrane and in nanodomains), and dynamics at the membrane (not only they reside in the same nanodomains but they have similar dynamics also arguing that these proteins are together). In addition, for the EFOP subunit, we show that it can delocalize two additional subunits of the complex when mistargeted to intracellular bodies. Together, we concluded that PI4Kα1/HYC1/NPG1 form a trimeric complex that is targeted to the plasma membrane by binding to a fourth subunit, EFOP, which is anchored at the plasma membrane via S-acylation.

Point 2. Analysis of pi4kα1mutants by electron microscopy revealed the presence of shriveled pollen grains with thick cell walls, a phenotype never observed in wild type plants. Based on these results and genetic analyses, the authors claim that PI4Kα1 is essential for pollen development. However, it is not clear to this reviewer whether the function of EFOP2-NPG-HYCC scaffold complex in targeting PI4K to the plasma membrane is responsible for this phenotype. Although high order mutants of hycc, epof and npgr families exhibit similar percentages of shriveled pollen grains, analysis by EM revealed important differences at the subcellular level. To this reviewer, it seems that only when EPOF2 activity is abolished, a thick cell wall can be observed in pollen grains, a phenotype resembling pi4kα1. Thus, it seems that the plasma membrane localization of PI4K is essential for pollen development, however further experiments are required to show the importance of PI4Kα1interaction with HYCC and NPG in this developmental context. Alternatively, the authors could make milder statements with respect to the importance of the complex in

pollen development. Analysis by EM of npg1 npgr1 npgr2 mutants should also be shown in figure 3. It would be also desirable to show the pollen localization of each subunit of the tetrameric complex in the pollen of Arabidopsis plants.

> **RESPONSE: We have now made new transmission electron microscopy observations in all the relevant allelic combinations showing shriveled pollen grains. The new micrographs are improved in quality and clearly show the presence of a thicker cell wall (intine) in pi4ka1 mutant, npg1npgr1npgr2 triple mutant, hyc1 single mutant, and efop3efop4 double mutant. These new results are presented in Figure 4. We are also showing the localization of each subunit in the pollen. We found that most isoforms could be detected at the plasma membrane in mature pollen (which is not always an exclusive localization). These results are presented in a new sup Figure 6.**

Point 3. The authors claim that they cannot study the developmental importance of PI4Kα1 function due to the pollen lethality observed in pi4kα1 mutants. However, the generation of inducible artificial microRNA against this gene should circumvent this problem. While it is clear that npg1 npgr1 npgr2 and hycc mutants show growth defects, it cannot be assumed that pi4k will have the same phenotypes. It may be possible that these proteins interact with other factors important to control these developmental processes. On those lines, the developmental defects described in Figure 4 should be included in the supplemental figure 4 and perhaps the statements about the importance of PI4K complex in general plant development should be re-phrased.

> **RESPONSE: We agree that it was not possible to conclude about the function of PI4Kα1 in the sporophyte in our previous analyses. We have now included a new figure (Figure 6) describing the phenotype of inducible artificial microRNA against PI4Kα1, which fully support the idea that the PI4K complex is not only required for gametophytic development but also for the development of the sporophyte. These new data are described 503 to 545 as the revised manuscript.**

Point 4. The authors hypothesize that the function of NPG's is to bridge EFOP2 with PI4Kα1. Is the subcellular localization of the latter affected in a npg npgr1/2 mutant background? What about HYCC localization? Is the activity of NPGs also necessary to target HYCC to the plasma membrane? The cogency of the manuscript would greatly profit from an additional experiment showing the subcellular localization of PI4K[illegible]1 and HYCC in npg npgr1/2 mutant background. Moreover, a schematic representation of the tetrameric complex and its subcellular localization/functions will facilitate the reader's comprehension of the manuscript.

> **RESPONSE: This is an excellent suggestion. We have now studied the localization of PI4Kα1 in the npg1+/-npgr1-/-npgr2-/- triple mutant using immunolocalization and our native PI4Kα1 antibody (see new Figure 10A). This new experiment shows that PI4Kα1 is largely soluble and aggregated inside the cell in the presence of a single functional copy of the NPG subunit. It thus further supports our model. These new experiment are now describe line 792 to 805 of the revised manuscript (in the manuscript with highlighted changes).**
>
> **We could not investigate the localization of HYC2 in the triple mutant because we don't have an antibody raised against this subunit. Crossing the mCITRINE-HYC2 line in the triple mutant would take a long time and we believe such data would not be as important as the localization of PI4Kα1, which is the enzymatic subunit in the complex.**
>
> **We have also included a model at the end of the manuscript (new Figure 10D and E), which recapitulates the formation of the tetrameric complex as we envision it and its possible interaction with the plasma membrane.**

TPC2020-RA-01032R1 2nd Editorial decision – *acceptance pending* **April 26, 2021**

We are pleased to inform you that your paper entitled "A nanodomain anchored-scaffolding complex is required for PI4Kα function and localization in plants" has been accepted for publication in The Plant Cell, pending a final minor editorial review by journal staff. At this stage, your manuscript will be evaluated by a Science Editor with respect to its presentation of scientific content, compliance with journal policies, and presentation for a broad readership.

Final acceptance from Science Editor **May 10, 2021**
